# Characterization of aberrant splicing in pediatric central nervous system tumors reveals *CLK1* as a candidate oncogenic dependency

**DOI:** 10.1101/2024.08.03.606419

**Published:** 2024-10-04

**Authors:** Ammar S. Naqvi, Ryan J. Corbett, Priyanka Seghal, Karina L. Conkrite, Komal S. Rathi, Brian M. Ennis, Katharina E Hayer, Bo Zhang, Miguel A. Brown, Daniel P. Miller, Adam A. Kraya, Joseph M. Dybas, Zhuangzhuang Geng, Christopher Blackden, Shehbeel Arif, Antonia Chroni, Aditya Lahiri, Madison L. Hollawell, Phillip B. Storm, Jessica B. Foster, Mateusz Koptyra, Peter J. Madsen, Sharon J. Diskin, Andrei Thomas-Tikhonenko, Adam C. Resnick, Jo Lynne Rokita

**Affiliations:** 1 Center for Data-Driven Discovery in Biomedicine, Children’s Hospital of Philadelphia, Philadelphia, PA, USA; 2 Division of Neurosurgery, Children’s Hospital of Philadelphia, Philadelphia, PA, USA; 3 Division of Oncology and Center for Childhood Cancer Research, Children’s Hospital of Philadelphia, Philadelphia, PA; 4 Division of Cancer Pathobiology, Children’s Hospital of Philadelphia, Philadelphia, PA, USA; 5 Department of Biomedical and Health Informatics, Children’s Hospital of Philadelphia, Philadelphia, PA, USA; 6 Department of Pediatrics, University of Pennsylvania, Philadelphia, PA, USA; 7 Department of Pathology and Laboratory Medicine, University of Pennsylvania, Philadelphia, PA, USA

## Abstract

Pediatric brain cancer is the leading cause of disease-related mortality in children, and many aggressive tumors still lack effective treatment strategies. We characterized aberrant alternative splicing across pediatric brain tumors, identifying pediatric high-grade gliomas (HGGs) among the most heterogeneous. Annotating these events with UniProt, we identified 11,940 splice events in 5,368 genes leading to potential protein function changes. We discovered *CDC-like kinase 1* (*CLK1*) is aberrantly spliced to include exon 4, resulting in a gain of two phosphorylation sites and subsequent activation. Inhibition of *CLK1* with Cirtuvivint significantly decreased both cell viability and proliferation in the pediatric HGG KNS-42 cell line. Morpholino-mediated depletion of *CLK1* exon 4 splicing reduced RNA expression, protein abundance, and cell viability with concurrent differential expression of 78 cancer genes and differential splicing at functional sites in 193 cancer genes. Our findings highlight a dependency of pediatric HGGs on *CLK1* and represent a promising therapeutic strategy.

## Introduction

Pediatric brain cancer is the number one cause of disease-related death in children^1^. Furthermore, pediatric high-grade gliomas (HGGs) are largely resistant to chemotherapy and are often surgically unresectable, making them exceptionally challenging^2^. Despite decades of clinical trials in pediatrics, patients with certain tumor types such as diffuse intrinsic pontine glioma (DIPG) or diffuse midline glioma (DMG) will succumb to their disease, with a median overall survival of 9.73 months in patients undergoing surgical biopsy^3^. Even with optimal multimodal therapy, median overall survival for non-DMG patients with a HGG is 14–20 months^4^. Within the last decade, surgical biopsies and post-mortem tissue collection has enabled genomic profiling and somatic characterization of pediatric HGGs. While somatic drivers such as mutations, fusions, and copy number amplifications and/or deletions have been well-characterized, the splicing landscape of pediatric HGGs remains underexplored. Two reports have shown rare, but mutually exclusive, alterations in spliceosome-related factors such as *SF3B1* and *SF3B2* in pediatric HGGs. These alterations dysregulated additional cellular processes involved in maintenance of DNA replication, genome integrity, or transcriptional fidelity^5,6^. More recently, Siddaway, et. al. describe alternative aberrant splicing as a novel mechanism of oncogenic pathway activation in pediatric HGGs. In this study, cancer drivers, including members of the RAS/MAPK pathway, were differentially spliced leading to the activation of the RAS/MAPK pathway and worse glioblastoma patient survival^7^. Alternative splicing plays critical roles in numerous fundamental biological processes, for example, increasing proteomic diversity, stabilizing or destabilizing mRNA transcripts, lowering protein steady-state levels, and influencing critical protein functionality such as enzymatic activity, protein folding, and localization^8,9^. Splicing plays a vital role in generating tissue-specific transcriptomes. The mammalian brain expresses the most complex and conserved alternative splicing programs in relation to other tissues and their disruption can result in a variety of neurological diseases and disorders^10,11^. The splicing process is regulated by a balance of multiple trans-acting RNA-binding proteins (RBP), such as the Serinerich Splicing Factors (SRSFs), that bind to splicing regulatory elements across preliminary mRNA transcripts. Disturbing that balance can be detrimental to cellular functionality, and has been associated with oncogenic transformation^12,13^.

Here, we perform a large-scale analysis of genome-wide splicing across pediatric CNS tumors and show widespread dysregulation of pre-mRNA alternative splicing across pediatric CNS tumors. Our findings reveal that alternative splicing can result in the removal or addition of known functional sites predicted to have significant downstream effects on various cellular functions potentially leading to cell viability and cell-cycle dysregulation. We identify and experimentally characterize *CLK1* as a candidate oncogenic dependency in pediatric HGGs and propose that targeting of aberrant splicing and/or its resulting downstream proteins may offer additional therapeutic avenues for precision cancer therapy.

## Results

### Pediatric brain tumors display heterogeneous global patterns of aberrant splicing

**Figure 1:**
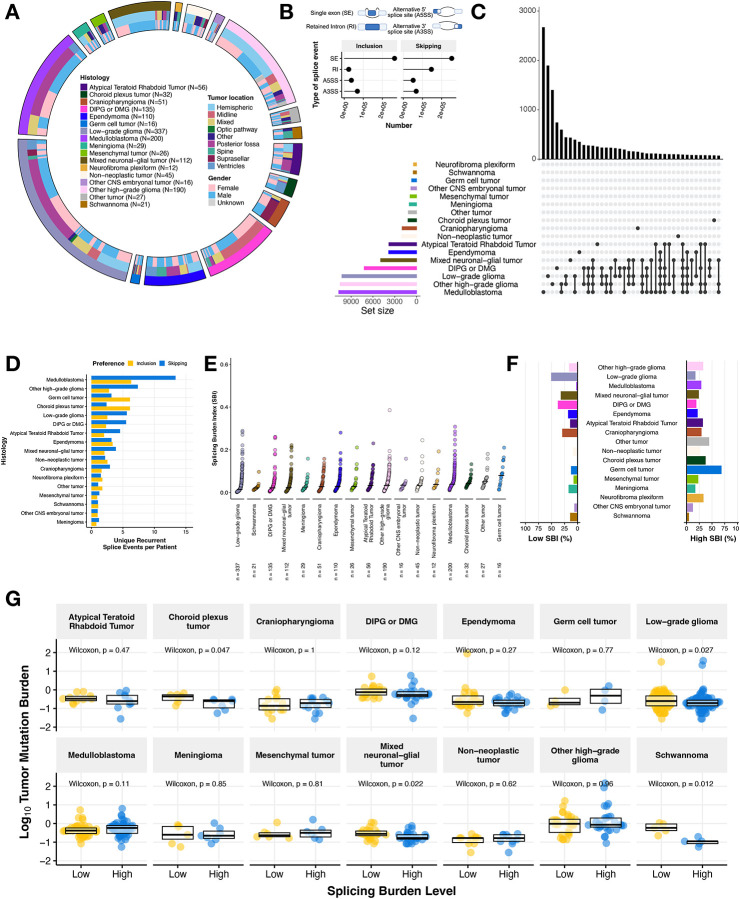
Pediatric brain tumors display heterogeneous global patterns of aberrant splicing **(A)** Circos plot of CNS tumors used in this study, categorized by histology, tumor location, and reported gender. Non-neoplastic tumors consist of benign tumors and/or cysts. **(B)** Lollipop plot illustrating the total number of splicing events across the cohort, classified by splicing type (SE: single exon, RI: retain intron, A3SS: alternative 3’ splice site, A5SS: alternative 5’ splice site). **(C)** UpsetR plot of recurrent differential splicing events that prefer exon skipping (N ≥ 2 of samples within a histology). **(D)** Barplots of the number of histology-specific recurrent events per patient. Histologies are reverse ordered by total number of unique events (skipping + inclusion). **(E)** Cumulative distribution plots of splicing burden index (SBI) by histology. **(F)** Barplots displaying percent of tumors with high (≥ third quantile) and low (≤ first quantile) SBI in each histology. **(G)** Boxplots of tumor mutation burden (TMB, log_10_) stratified by high or low SBI by histology. Withinhistology Wilcoxon p-values are shown. All boxplots represent the 25th and 75th percentile and the bar represents the median.

We utilized replicate Multivariate Analysis of Transcript splicing (rMATs)^14,15^ to quantify transcriptome-wide alternative splicing events across primary pediatric brain tumors (N = 1,415) from the Open Pediatric Cancer (OpenPedCan) project^16^. These broad tumor types shown in Figure 1A include Atypical Teratoid Rhabdoid Tumor (ATRT, N = 56), choroid plexus tumor (CPT, N = 32), craniopharyngioma (CPG, N = 51), diffuse intrinsic pontine glioma or diffuse midline glioma (DIPG or DMG, N = 135), ependymoma (N = 110), germ cell tumor (N = 16), low-grade glioma (LGG, N = 337), medulloblastoma (MB, N = 200), meningioma (N = 29), mesenchymal tumor (N = 26), mixed neuronal-glial tumor (GNT, N = 112), neurofibroma plexiform (N = 12), non-neoplastic tumor (N = 45), other CNS embryonic tumor (N = 16), other high-grade glioma (other HGG, N = 190), schwannoma (N = 21), and other rare brain tumors (N = 27). Associated demographic and clinical data for each patient and tumor in this study are available in Table S1. We examined four types of splicing events (single exon: SE, alternative 5’ splice site: A5SS, alternative 3’ splice site: A3SS, and retained intron: RI) and observed that SE splicing is overwhelmingly the most frequently observed (Figure 1B) for both increased inclusion and skipping events, consistent with a previous report in pediatric HGGs^7^. Importantly, we did not observe an effect of RNA library on percent spliced in (PSI) predictions and found that the sample aliquots sequenced with a poly-A or stranded library had strong positive correlations for genome-wide PSI values (Pearson’s R = 0.98, p-value < 2.2e-16, Figure S1A–B).

Due to the diverse biological drivers of these CNS cancers and their molecular subtypes, we hypothesized that we might observe histology- or subtype-specific splicing patterns. Indeed, our assessment of recurrent exon skipping (ES) or exon inclusion (EI) events (N ≥ 2) revealed histology-specific splicing events in some tumor types, shared events across some histologies, as well as lack of recurrent events in other histologies when focusing on the top 40 sets that can comprise one histology or multiple histologies (Fig 1C, ES events and Figure S1C, EI events). Within these histologies, we found that MB, LGG, and HGG tumors exhibited the highest number of recurrent, unique skipping events (MB = 2,644, LGG = 1,860, other HGG = 1,610, DMG = 687) and inclusion events (MB = 1,235, LGG = 814, other HGG = 677). To ensure this observation was not skewed by the number of patient tumors per histology grouping, we normalized these events by the number of patients. We observed that MB and HGG tumors had the highest total number of unique, recurrent single exon events per patient (Fig 1D). We plotted the top 40 sets with a complete list of unique events per histology reported in Table S2A–B. Taken together, these findings suggest that there are both tumor type-specific and shared splicing patterns in pediatric brain tumors.

We further investigated single exon-associated events and devised a metric called the splicing burden index (SBI) to compute the proportion of differential alternative splicing (AS) events in each sample compared to all other tumor samples (Figure 1E, see Methods). This metric allows for transcriptome-wide assessment of differential AS within a tumor sample. The median SBI across this cohort was 0.0296 (or 2.96%). LGG tumors had the lowest median SBI (0.0150, 1.50%), while germ cell tumors had the highest (0.0802, 8.02%). Tumors with a low splicing burden variance (< 1st quantile variance across all tumors) include other tumors, other CNS embryonal tumors, meningiomas, choroid plexus tumors and schwannomas, while more heterogeneous tumors (splicing burden variance > 0.04641) include DIPG or DMG, MB, mesenchymal tumors, neurofibroma plexiform, and germ cell tumors. We performed a similar analysis on the other splicing cases (A5SS, A3SS, and RI) and observed that LGGs and schwannomas continued to exhibit the lowest median SBI across splice types, while other tumors and germ cell tumors (GCTs) maintained the highest median SBI. However, the ordering of tumors shifted in some instances, suggesting that certain types of splice events may be more prevalent in some histologies compared to others. For example, DIPG or DMG tumors changed from having a low median SBI for SE events to a higher median SBI for other event types, suggesting an increased splicing burden for RI, A5SS, and A3SS splicing changes (Figures S1D–F). We next assessed the proportion of low and high SBI tumors in the cohort by histology. The majority (> 50.7%, N = 171/337) of LGGs consisted of tumors with low SBI, while the majority of germ cell tumors (N = 11/16) had a high SBI (Figure 1F). Moreover, greater than 25% of tumors within the following histologies had a high SBI: Other high-grade glioma, medulloblastoma, ATRT, craniopharyngioma (CPG), other rare tumors, CPT, germ cell tumor, and neurofibroma plexiform, while greater than 25% of tumors within the following histologies had a low SBI: LGG, mixed GNT, DIPG or DMG, and CPG. These results further highlight the heterogeneous splicing landscape across pediatric CNS tumors.

We next hypothesized that tumors with a low tumor mutation burden (TMB) might have a higher splicing burden index as an alternate mechanism driving tumorigenesis. Interestingly, we did not find an overall correlative relationship between TMB and SBI when analyzing the full CNS tumor cohort together (all tumors: Pearson’s R = 0.03, p-value = 0.3 and with hyper-mutant tumors removed: R = 0.012, p-value = 0.68, Figure S1G–H), but when assessing the relationship by histology (Figure S1I), we found significant negative correlations between TMB and SBI in LGG (Pearson’s R = −0.15, p-value = 0.0059), DIPG or DMG (Pearson’s R = −0.24, p-value = 0.02), and Schwannomas (Pearson’s R = −0.77, p-value = 0.00018). As an alternate approach, we asked whether TMB distribution was different between tumors with high and low SBI within histologies (Figure 1F). We found a significant inverse relationship between SBI and TMB in only CPTs, LGGs, GNTs, and schwannomas (Wilcoxon p-value < 0.05), indicating that aberrant splicing may serve as an alternative reservoir for uncovering oncogenic mechanisms in mutationally silent tumors.

### Splicing drives novel biological clusters and splicing burden differentiates key splicing factors in pediatric high-grade gliomas

**Figure 2:**
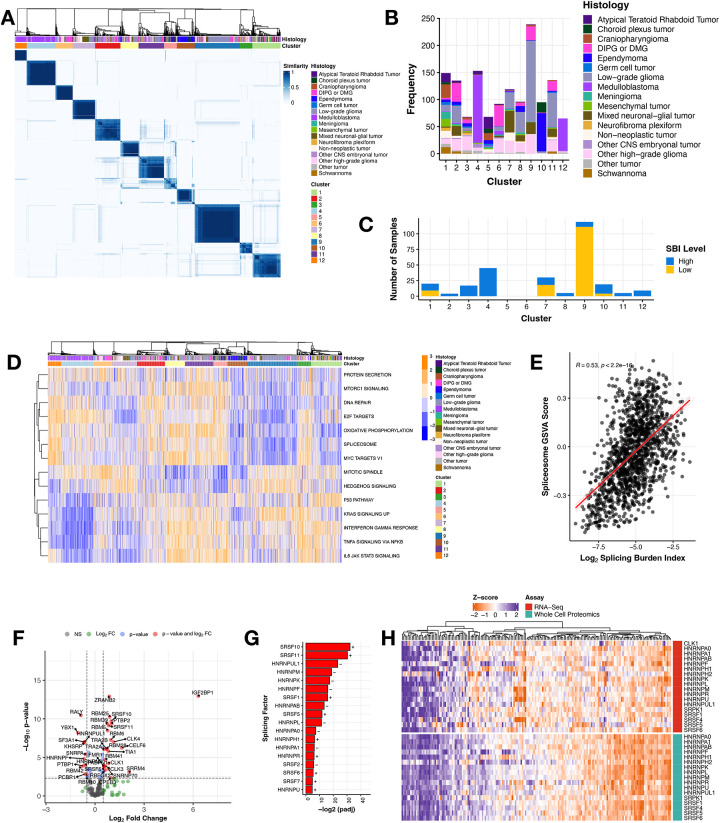
Splicing drives novel biological clusters and splicing burden differentiates key splicing factors in pediatric high-grade gliomas **(A)** Consensus clustering heatmap of PSI values for all expressed genes (junction read counts ≥ 10) with a multi-modal distribution across tumors (see Methods). **(B)** Stacked barplot showing histology sample membership in each cluster. **(C)** Stacked barplot of the number of tumors with high or low SBI within each cluster. **(D)** Heatmap of top cancer-related enriched pathways by cluster (GSVA scores represented by blue/orange color). **(E)** Pearson’s correlation scatterplot of log_2_ SBI and KEGG Spliceosome GSVA score (R = 0.53, p-value < 2.2e^−16^). **(F)** Volcano plot illustrating the expression direction of splicing factor genes in HGGs with high SBI compared to those with low SBI (NS = not significant, FC = fold change, colored dots represent log_2_FC > |.5| and/or Benjamini and Hochberg adjusted p-value < 0.05). **(G)** Barplot presenting members of the hnRNP and SRSF families of primary splicing factors that are differentially expressed (Benjamini and Hochberg adjusted p-value < 0.05) with directionality (+ or −). **(H)** Heatmap displaying these splicing factors and known regulators *CLK1* and *SRPK1* for all pediatric brain tumors with available RNA-seq and whole-cell proteomics data from the CPTAC portal.

To determine whether CNS tumors share transcriptional splicing biology, we performed consensus clustering of PSI values across all primary CNS tumors using the Partition Around Medoids (PAM) algorithm with binary distance metric, which revealed 12 clusters spanning histologies (Figure 2A). A full list of clustering parameters and statistics tested are detailed in Table S2C. Clusters 4 and 12 were predominantly composed of MB (Figure 2B) with both containing all four MB subtypes: SHH, WNT, Group 3, and Group 4 (Figure S2C), suggesting unique splice-driven biological underpinnings even within molecularly-defined subtypes. Although Cluster 11 was dominated by EPNs (Figure 2B), it comprised all EPN molecular subtypes (Figure S2C). While Cluster 4 contained the majority of LGGs in the cohort, LGGs spanned nine of 12 clusters (Figure S2C) and clusters were not subtype-specific. Notably, HGGs, including DIPG or DMG, exhibited the highest degree of splicing heterogeneity, spanning across all 12 clusters (Figure 2B and S2C). Taken together, the transcriptional mechanisms underlying these clusters’ formation are not solely driven by molecular subtype. A full list of samples with associated cluster membership information is outlined in Table S2D. We further assessed cluster membership of tumors with high or low SBI and found that tumors with high SBI were present in ten clusters, while the low SBI tumors grouped almost entirely with Cluster 9, followed by Clusters 1, 7, and 10 (Figure 2C). We aimed to understand the biology driving cluster formation and used gene set variation analysis (GSVA) to identify enriched cancer-associated signaling pathways represented by splice events in each of the 12 clusters. Figure 2D displays the top pathways differentially-regulated between pairs of clusters (Bonferroni-adjusted p-value < 0.05). Strikingly, the spliceosome pathway was significantly up- or down-regulated in most clusters when performing pairwise comparisons to all other groups (Bonferroni-adjusted p-value < 5.38^e−68^ - 1.96^e−2^). Clusters 4 and 6 had samples with heterogeneous spliceosome scores. Further, we show a significant positive correlation (Figure 2E, Pearson’s R = 0.53, p-value < 2.2e-16) between GSVA spliceosome pathway scores and SBI, both validating the use of the SBI metric to measure splicing activity and suggesting that splicing factors themselves are mis-spliced, likely contributing to the transcriptome-wide differential splicing we are observing within pediatric brain tumors. Other pathways that were enriched for certain clusters included DNA repair, mitotic spindle, and KRAS signaling. For example, Cluster 4 was dominated by MB tumors, including subtype Group 4 (Figure S2C) which also has known dysregulation of the KRAS signaling pathway, corroborating early characterization of these tumor types^17^. These results reveal that although we observe transcriptome-wide splicing variation in all tumors, distinct pathways and genes are targeted in each cluster. Taken together, we show that each cluster may have unique transcriptional underpinnings influencing distinct pathways which may, in turn, contribute to tumorigenesis.

Given their cluster and SBI heterogeneity and high number of unique, recurrent splice events, we narrowed our focus to all pediatric HGGs. Since we sought to understand the mechanisms underlying the widespread and pervasive splicing, we first assessed somatic alterations in splicing factor-encoding genes or splicing regulators in our cohort. Interestingly, a recent splicing study found that 34% of pediatric HGGs had a somatic alteration, mutation and/or CNV, in a gene in the HUGO spliceosome complex^7^. Since few of these genes overlap with the splicing factors and their canonical regulators assessed in our study, we combined our gene lists and investigated somatic alterations in these genes. We removed hypermutant tumors (≥ 10 Mut/Mb), filtered for putative deleterious mutations (defined by SIFT and/or PolyPhen), and found that 118 HGG tumors from 186 patients (63.44%) harbored at least one somatic SNVs/InDel, fusion, or CNV in a gene in the spliceosome complex or that regulates alternative splicing (Table S3A, Supplemental Figure S2D). However, the mutation frequencies in each gene only ranged from 1–8%, and there was no significant enrichment in any gene based on SBI status. It has been previously shown that in the absence of splicing factor gene mutations, RNA expression changes in these genes can cause downstream splicing changes to promote tumor formation^18–20^. Thus, we performed differential gene expression (DE) analysis between high vs low SBI HGGs for known splicing factors and related genes^21^ (Figure 2F) and found 44.3% (N = 77/174) to be significantly differentially expressed (adjusted p-value < 0.05 and log_2_-fold change > |2|, Table S2). Specifically, 64% (18/28) genes encoding the serine/arginine-rich splicing factor (SRSF) and heterogeneous nuclear ribonucleoproteins (hnRNP) families of trans-acting splicing factors known to directly influence exon-associated splicing were significantly DE between high vs low SBI HGGs (Figure 2G). Since changes in gene expression do not always result in similar changes at the protein level, this finding prompted us to investigate protein expression of these splicing factors. We integrated gene expression and proteogenomic (N = 188) data from pediatric brain tumors obtained from the Clinical Proteomic Tumor Analysis Consortium (CPTAC)^22^. We observed that mRNA and protein expression for these splicing factors are tightly coordinated across pediatric CNS tumors (Figure 2H), supporting previous reports that differentially-expressed mRNAs are better correlated to protein levels than non-differentially expressed mRNAs^23^. As such, mRNA levels of these splicing factor genes can be used as surrogate measurements for protein abundance and function.

### Recurrent splicing aberrations alter known proteomic functional sites, including the gain of phosphorylation binding sites in splicing regulator *CLK1*

**Figure 3:**
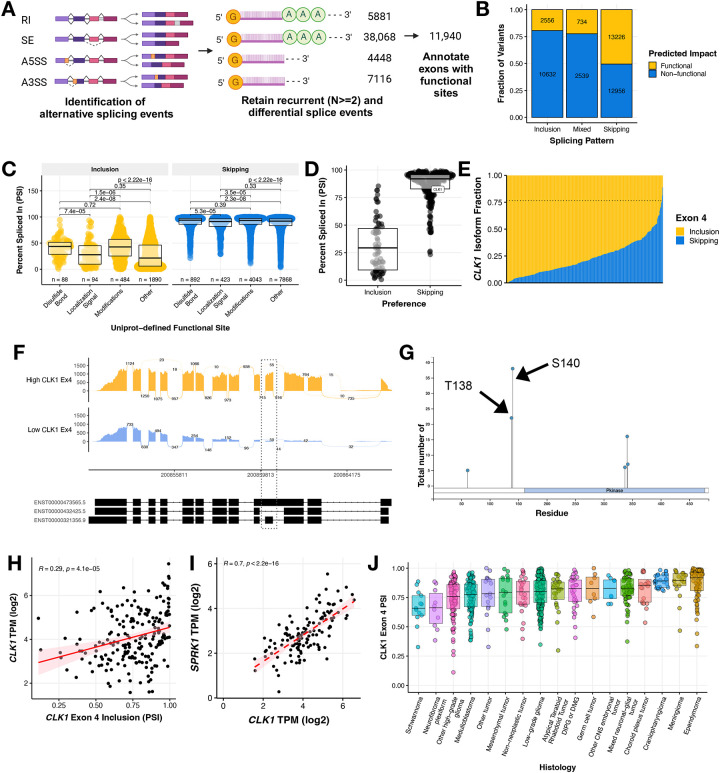
Recurrent splicing aberrations alter known proteomic functional sites in pediatric high-grade gliomas, including phosphorylation sites in splicing regulator protein kinase *CLK1* **(A)** Workflow to identify 11,940 differential exon-level splicing events that alter UniProt-defined functional sites in HGGs. **(B)** Stacked bar plots showing the fraction of exon inclusion, skipping, or mixed splicing events categorized by predicted impact. **(C)** Boxplots of splice events resulting from gain or loss of functional sites categorized by UniProt annotation. Wilcoxon between-group p-values are shown. **(D)** Boxplots of predicted functional splice events affecting known kinases with *CLK1* highlighted. **(E)** Stacked barplot of *CLK1* exon 4 inclusion and skipping isoform fraction in HGGs. Dotted line represents the mean PSI of 0.7653. **(F)** Sashimi plot of two representative tumor samples with either high (BS_HRJ9145M) or low (BS_XM1AHBDJ) *CLK1* exon 4 inclusion. **(G)** PhosphositePlus^46^ CLK1 protein visual highlighting the two phosphorylation binding sites in exon 4. **(H)** Pearson’s correlation scatter plot of *CLK1* exon 4 PSI and RNA expression in HGG tumors (R = 0.29, p = 4.1e^−5^). **(I)** Pearson’s correlation scatter plot of *CLK1* exon 4 PSI and *SRPK1* RNA expression in HGG tumors (R = 0.7, p = 2.2e^−16^). **(J)** Boxplot of CLK1 Exon 4 PSI levels across all primary pediatric brain tumors. All boxplots represent the 25th and 75th percentile and the bar represents the median.

In order to further elucidate the aberrant splicing landscape of pediatric HGGs (N = 325), we developed a robust and adaptable workflow to prioritize recurrent single exon events with predicted functional impact (Figure 3A). We first applied a threshold of ≥ 10 junction read counts to identify expressed splice events. Next, we prioritized histology-specific recurrent (N ≥ 2) events that were differentially spliced (ΔPSI z-score > |2|) in a sample compared to the whole cohort. We identified a total of 38,068 recurrent differential splicing events in HGGs. Subsequently, we annotated these events for overlap with known Uniprot functional sites and prioritized those leading to gain or loss of a functional site affecting disulfide bonding, localization signaling, amino acid modifications, or others. The Uniprot annotated “other” category is user-defined, but includes sites for ion-binding, calcium binding, PDZ-binding-motif and more^24^. This reduced the number of prioritized splice events to 11,940 events in 5,368 genes with a putative functional effect. Among these predicted exon splicing variants targeting functional sites illustrated in Figure 3B, the majority favored increased exon skipping (N = 13,226), with smaller subsets favoring increased exon inclusion (N = 2,556) or displaying a mixed pattern (N = 734). Shown in Figure 3C, these functional sites included changes to disulfide bonding (N_EI_ = 88, N_ES_ = 892), localization signaling (N_EI_ = 94, N_ES_ = 423), amino acid modification (N_EI_ = 484, N_ES_ = 4,043), and other functional sites (N_EI_ = 1,890, N_ES_ = 7,868). Each event is listed in Table S4. The remaining splicing events were associated with un-annotated sites, untranslated, and/or non-coding regions. To identify potentially targetable events, we selected functional splice events in kinases and performed over-representation pathway analysis which revealed MAPK, ERBB, and PI3K-AKT MTOR as the top cancer-related pathways significantly over-represented (Bonferroni-adjusted p-value < 0.05, Figure S3B–C).

The gene encoding protein kinase *CDC Like Kinase 1 (CLK1)*, an oncogenic factor and known master modulator of alternative splicing^12^, was amongst this subset of differentially spliced kinase genes in HGGs (Figure 3D). The majority of tumors showed very high levels of *CLK1* exon 4 inclusion (Figure 3E) with mean PSI of 0.7653 (or 76.53%) and thus the inclusion event was not differential in most HGGs. In contrast, 12 tumors demonstrated significant skipping (decreased inclusion) of exon 4 (ΔPSI <= −2 z-scores of mean PSI), thus driving the differential splicing observed for *CLK1* exon 4 (Figure 3D). Additionally, we observed *CLK1* exon 4 associated transcript expression heterogeneity across non-tumor brain controls from the Genotype Tissue Expression Project (Figure S3C). *CLK1* regulates the SR (Serine aRginine) family of splicing factor proteins through hyper-phosphorylation of the SR-rich peptide regions of SR proteins to induce cooperative RNA binding and increased activity^25–27^. Moreover, the differential splicing of *CLK1* resulted in differences of exon usage across HGGs (Figure 3E–F). *CLK1* exon 4 contains two catalytic sites, Thr138 and Ser140 (Figure 3G), and these have been described previously to be associated with increased protein abundance^26^. *CLK1* exon 4 inclusion was significantly positively correlated with expression of total *CLK1* mRNA (Pearson’s R = 0.29, p-value = 4.1e-5, Figure 3H), supporting the hypothesis that inclusion of these phosphorylation sites in exon 4 increases canonical *CLK1* expression. Further, *CLK1* expression is significantly positively correlated with expression of *Serine/Arginine-rich protein-specific kinase 1* (*SRPK1*), a kinase that cooperates with SR-bound *CLK1* to facilitate SR phosphorylation, U1 exchange of *CLK1*, and subsequent splicing^28^ (Figure 3I, Pearson’s R = 0.7, p-value < 2.2e-16). High *CLK1* exon 4 inclusion was not unique to HGGs though they were the most heterogeneous. Indeed, we observed widespread high median *CLK1* exon 4 inclusion levels across pediatric brain tumors, suggesting these tumors contain active *CLK1* (Figure 3J).

### *CLK1* is an oncogenic dependency in pediatric HGGs

**Figure 4.**
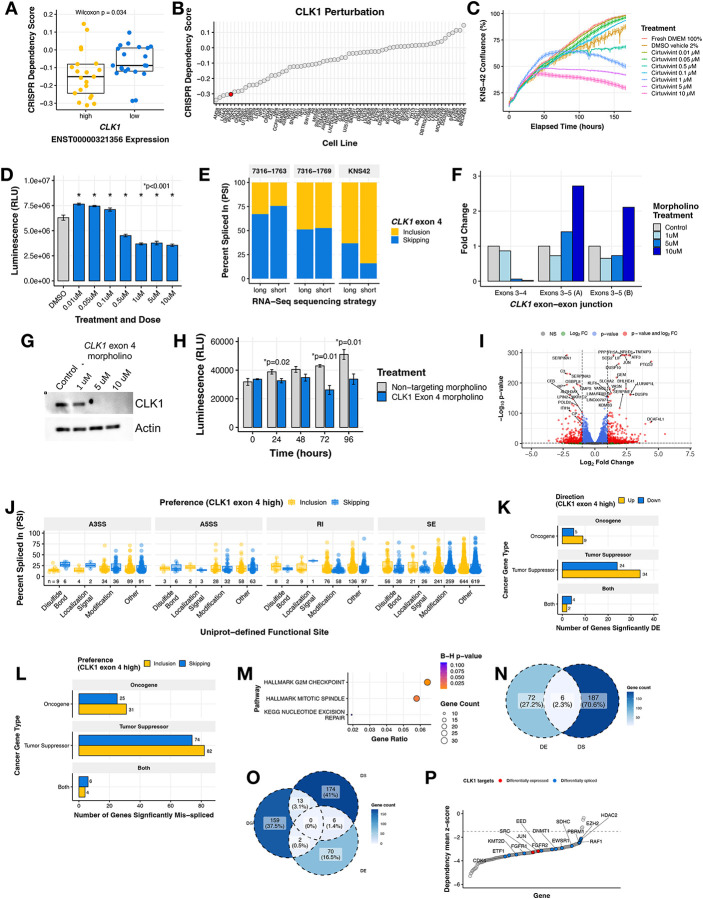
CLK1 aberrant splicing contributes to oncogenesis in brain tumor cell lines. **(A)** Boxplot of DepMap dependency scores stratified by high or low *CLK1* exon 4 containing transcript expression in brain tumor cell lines. Wilcoxon p-value shown. **(B)** Ranked dotplot of DepMap dependency scores in brain tumor cell lines with pediatric line KNS-42 highlighted in red. **(C)** Proliferation of KNS-42 cells treated with increasing concentrations of pan-DYRK/CLK1 inhibitor Cirtuvivint over six days. **(D)** Day 6 cell viability of KNS-42 cells treated with increasing concentrations of Cirtuvivint. Stars denote Bonferroni-adjusted p-values following pairwise Student’s *t-tests*. **(E)** Stacked bar plots of the percent inclusion and skipping of *CLK1* exon 4 transcripts in patient-derived cell lines (7316–1763 and 7316–1769 from the CBTN) and KNS-42 (commercial) derived using either long (ONT) or short RNA-seq strategies. **(F)** Barplot showing the RNA expression fold-change in cells treated with control morpholino or morpholino targeting the *CLK1* exon 3–4 junction or exon 3–5 junction **(G)** Western blot of CLK1 with increasing morpholino treatment of 1, 5, and 10 μM. **(H)** Cell viability of cells treated with *CLK1* exon 4 morpholino or non-targeting morpholino. Stars denote within-time paired Student’s *t-tests*. **(I)** Volcano plot illustrating genes differentially-expressed in KNS-42 cells treated with *CLK1* exon 4 targeting morpholino compared to cells treated with non-targeting morpholino. **(J)** Boxplot of |ΔPSI| of significantly differential splicing events comparing KNS-42 cells treated with *CLK1* exon 4 targeting morpholino vs. non-targeting morpholino (ΔPSI ≥ |.10|, p-value < 0.05, FDR < 0.05). Plot shows Uniprot-defined functional sites which are gained/lost categorized by splicing case (A3SS, A5SS, RI, and SE). **(K)** Barplots displaying number of differentially expressed (DE) genes or **(L)** differentially spliced (DS) genes affecting functional sites categorized by gene family. **(M)** Over-representation analysis using ClusterProfiler of DS cancer genes that result in gain/loss of functional sites. **(N)** Venn diagram depicting overlap of DS and DE genes from K and L **(O)** Venn diagram depicting overlap of DS and DE genes from K and L and significant (Wald FDR < 0.05, z-score < −1.5) essential genes identified in matched CBTN HGG cell lines through CRISPR dependency experiments from the Childhood Cancer Model Atlas (CCMA v3). **(P)** Ranked dotplot of significant CRISPR gene dependency mean z-scores for pediatric HGG cell lines with *CLK1* expression and splicing-based target genes highlighted in red and blue respectively.

We sought to further examine the role of *CLK1* as a potential oncogene in HGGs. We investigated the cancer Dependency Map (DepMap) portal and database and found that CNS and brain tumor cell lines with high expression of the exon 4 included transcript of *CLK1* (≥ third quantile mRNA expression of ENST00000321356) have significantly higher CRISPR dependency (lower scores) compared to *CLK1* low expressing cell lines (≤ first quantile) (Wilcoxon p = 0.034, Figure 4A). This observation was significant and unique only to cell lines derived from CNS tumor and myeloid malignancies (Figure S4A), suggesting tissue- and tumor-specific regulation of *CLK1*. Across all DepMap profiled cell lines, we found that the pediatric brain tumor cell line KNS-42 had a strong dependency on *CLK1* (low CRISPR dependency score) (Figure 4B) and chose it for further *in vitro* testing. We next tested the impact of CLK1 inhibition in KNS-42 cells using the pan-Dyrk/Clk inhibitor Cirtuvivint (SM08502)^29^. Using the IncuCyte to monitor real-time proliferation, we observed a significant reduction in cell growth at multiple concentrations over a 6-day period (Figure 4C). Additionally, we observed a dose-dependent decrease in cell viability using CellTiter-Glo at three days (Figure 4D) and six days (Figure S4B) post-treatment of 0.5, 1, 5, and 10 μM Cirtuvivint.

Based on these findings and to rule out off-target effects of the pan-inhibitor, we selected KNS-42 along with two additional cell lines from our pediatric brain tumor cohort with high *CLK1* exon 4 PSI (7316–1763 and 7316–1769) to experimentally validate the exon 4 splice event identified from short-read RNA-Seq. We performed long-read RNA-seq using Oxford Nanopore Technologies (ONT) and validated the presence of two major full-length *CLK1* mRNA isoforms that either included or skipped exon 4 across these three patient-derived cell lines (Figure 4E).

We therefore postulated that the gain of *CLK1* phosphorylation sites on exon 4 increases mRNA and subsequent protein production in HGGs. To directly test this hypothesis, we modulated *CLK1* exon 4 splicing using targeted morpholino oligomers (see Methods), in which we forced exon 4 skipping in the KNS-42 cell line. We performed qRT-PCR and observed a near total loss of the *CLK1* exon 4 inclusion transcript at both 5 and 10 μM of exon 4 targeted morpholino, evidenced by reduced expression of the exon 3–4 junction. Likewise, at these same concentrations, we observed increased *CLK1* exon 4 skipping using primers targeting the exon 3–5 junction (Figure 4F). Importantly, forced *CLK1* exon 4 skipping resulted in ablation of CLK1 protein at 5 and 10 μM (Figure 4G), corroborating previous work that *CLK1* exon 4 is required for full-length and catalytically active *CLK1*^30–32^. Next, we assessed the functional impact of *CLK1* exon 4 splicing using CellTiter-Glo and confirmed that cells with high *CLK1* exon 4 skipping (*CLK1* exon 4 targeting morpholino) exhibited significantly decreased viability compared to those with *CLK1* exon 4 inclusion (non-targeting morpholino) at 24, 72, and 96 hours (p ≤ 0.01, within-time Student’s *t-test*, Figure 4H). Taken together, we demonstrate that *CLK1* is a dependency in pediatric HGGs required for cellular growth and viability and CLK1 mRNA and protein is maintained through increased exon 4 inclusion.

To identify bona-fide *CLK1* targets mediated by exon 4 splicing, we performed RNA-seq from KNS-42 cells treated with morpholino oligomers (N = 3 controls, N = 3 targeted to skip exon 4). We performed differential gene expression (DE) analysis and identified 296 genes with differential expression (193 upregulated, 103 downregulated) between the treated and untreated populations (Figure 4I, Table S5A). Next, we quantified differential alternative splicing (Methods, Table S5B) and applied the same downstream computational workflow from Figure S3A to prioritize splice events affecting functional sites. We identified a total of 2,006 unique differential splicing (DS) events within 1,467 genes predicted to alter functional sites (SE = 1,905, A5SS = 196, A3SS = 272, and RI = 388, Figure 4J and Table S5C–F). These dysregulated genes included TSGs and oncogenes involved in RNA-binding, epigenetics, transcription factors, and kinases (Figures 4K–L and Table S5G). These genes were over-represented in G2M checkpoint, mitotic spindle, and nucleotide excision repair pathways (Figure 4M). To further investigate the impact on DNA repair and other pathways, we performed gene-set enrichment analyses of DNA repair and cancer signaling pathways on these DS oncogenes and TSGs and found that depletion of CLK1 leads to upregulation of TNFA, PI3K/AKT/MTOR, IL6/JAK/STAT3, and apoptosis pathway expression and downregulation of multiple DNA repair pathways (Figure S4C–F). Moreover, of the cancer genes with putative functional consequences driven by *CLK1* splicing, we discovered that 2.3% (N = 6) had dysregulation at the level of both splicing and expression (Figure 4N), indicating these may impact the tumor’s proteome. The DE genes were significantly over-represented (Bonferroni-adjusted p-value <0.05) for KRAS upregulation and drug metabolism pathways (Figure S5A) while DE genes were significantly over-represented (Figure S5B) in G2M checkpoint, mitotic spindle, and nucleotide excision repair pathways, suggesting a potential for these events to impact cellular functions, contribute to the cancer disease state, and/or play a role in regulatory mechanisms of gene expression.

Finally, we asked whether *CLK1* splicing affects any of the essential oncogenes defined by the pediatric gene dependency maps of the Childhood Cancer Model Atlas^33^. We observed 15 of these genes also exhibit significant gene dependencies (GD) in established pediatric HGG cell lines (Figure 4O–P, Table S5B), including *CDK4*, *FGFR1*, *FGFR2*, *EZH2*, *RAF1*, and *SRC*. For instance, the expression levels of mRNAs encoding proto-oncogene *SRC*^34,35^ are higher in cells with high *CLK1* exon 4 (non-targeting morpholino), indicating that *CLK1* may enhance or promote *SRC* expression. The differential splicing cases are more complex as they affect multiple transcripts, but taken together, these data suggest that transcript-level changes mediated through *CLK1* could be contributing to some of these dependencies, particularly given the association of aberrant splicing with cancer progression^36–38^.

## Discussion

Pediatric brain cancer remains the leading cause of disease-related mortality in children, and HGGs present formidable challenges due to their resistance to chemotherapy and surgical limitations. In this study, we conducted a large-scale analysis of aberrant alternative splicing across pediatric CNS tumors, revealing widespread dysregulation of pre-mRNA alternative splicing. We developed an analytical framework to prioritize and predict the consequences of splicing events. Our study demarcates intricate splicing patterns across various tumor types and introduces the splicing burden index (SBI) as a novel metric to quantify differential splicing events at the sample level without requiring a normal control. We discovered significant negative correlations between tumor mutation burden (TMB) and SBI in CPT, LGG, GNT, and schwannoma, suggesting that aberrant splicing may serve as a compensatory mechanism for tumorigenesis in these mutationally silent tumors. Further analysis of skipped exon events unveiled novel biological clusters driven by splicing variations across histologies and molecular subtypes, highlighting extensive splicing heterogeneity in pediatric HGGs. Of note, molecular subtypes were not cluster-specific and specifically for MB, this differs from a previous report which showed that subgroups WNT, SHH, Group 3, and Group 4 can be clustered using splicing information^39^. This is due to fundamental differences in clustering procedure and study goals. We performed unsupervised clustering to identify novel groupings, whereas Dubuc and Morrissy, et. al performed supervised clustering following identification of differentially expressed splice events among the four subgroups in order to classify groups using splice events^39^. Utilizing the UniProt Knowledgebase, we identified splice variants in HGGs that alter functional sites, potentially impacting protein functions such as activation, folding, and localization.

A key finding was differential splicing of *CDC-like kinase 1 (CLK1)*, a critical splicing factor and cell-cycle modulator in pediatric HGGs. This splicing event led to the inclusion of phosphorylation sites in exon 4, promoting increased protein abundance. Experimental modulation of *CLK1*, either through inhibition or morpholino-directed exon 4 depletion in the KNS-42 cell line, resulted in significantly reduced cell proliferation and/or viability. Splicing modulation to deplete exon 4 ablated CLK1 RNA and protein levels, altogether supporting *CLK1* as a gene dependency in pediatric HGGs. Additionally, we identified transcriptional dysregulation of essential cancer genes mediated by aberrant *CLK1* splicing. *CLK1* is currently being targeted therapeutically with the Pan-Clk/Dyrk Inhibitor Cirtuvivint (SM08502) in heme malignancies^29^ and non-CNS solid tumors such as castrate-resistant prostate cancer, colorectal cancer, and non-small cell lung cancer^40–42^. Here, our study suggests that *CLK1* may also represent a therapeutic vulnerability in CNS malignancies including pediatric HGGs.

This investigation enhances our understanding of the splicing landscape in pediatric brain tumors and proposes that aberrant splicing may be a viable target for therapeutic intervention. Further, we openly share the splicing data for all pediatric CNS tumors and believe this can be a valuable resource for the oncology community. Our approach to characterizing splicing aberrations and their functional consequences paves the way for future research into mRNA splicing-based mechanisms of tumorigenesis, the identification and development of therapies targeting aberrant splice events, and may even guide splicing-based diagnostics, all of which have the potential to improve the therapeutic landscape for pediatric brain cancers.

### Limitations of the study

In this study, splicing quantifications were primarily performed using short-read RNA-Seq technology, which limits the interpretation of the full spectrum of splicing variation, particularly larger multi-exon transcripts or with genes that contain a high number of transcripts. While there are proteomics data for over 200 matched pediatric brain tumors publicly available, we were limited in sample size for DIPG or DMG and other HGGs, so it will be important to validate our findings in larger datasets as they become available. Additionally, the lack of pediatric normal tissue RNA-Seq necessitated use of non-tumor controls restricted to adult samples from GTEx, and may not necessarily represent the splicing landscape of pediatric tissues. Further, GTEx does not contain tissue of origin for all pediatric brain tumors (eg: pons for DIPG or brainstem for DMG), further limiting the comparison. Additionally, within histologies (eg: LGG), the primary site of the tumor can vary widely depending on diagnosis and it would be ideal to match each tumor to a normal one by one. Although this is not yet possible with the normal RNA-Seq available, the creation of the upcoming developmental GTEx will be critical in the future. We mitigated these normal tissue limitations through the use of the SBI metric, in which we compared each tumor to every other tumor in the cohort. Finally, this RNA-Seq cohort contained samples with many different library preparation strategies (poly-A, stranded, exome capture, stranded poly-A), which ultimately leads to batch effects in certain expression analyses. Using two samples sequenced by different library preparation strategies, we demonstrated that SBI was largely unaffected by library type (Figure S1A–B). Therefore, to mitigate batch effects with gene expression measurements, we used the entire pooled cohort for splicing analyses (PSI, SBI) but used only stranded samples in other analyses involving gene expression values such as correlations, differential expression, and/or over-representation analyses. Overall, we employed robust statistical techniques, cross-validated our findings with external datasets, and utilized orthogonal approaches and experimental methods where possible.

## STAR Methods

### RESOURCE AVAILABILITY

#### Lead Contact

Requests for access to raw data and/or specimens may be directed to and will be fulfilled by Jo Lynne Rokita (rokita@chop.edu).

#### Materials availability

This study did not create new, unique reagents.

#### Data and code availability

##### PBTA patient genomic data

All pediatric brain tumor raw data are available upon request from the database of Genotypes and Phenotypes (dbGAP), accession number phs002517.v2.p2, and/or from the Children’s Brain Tumor Network (https://cbtn.org) and the Pacific Pediatric Neuro-Oncology Consortium (pnoc.us) for data not immediately available in dbGaP. All processed data used in this study were derived from the OpenPedCan project^16^ v13 data release at https://github.com/d3b-center/OpenPedCan-analysis. All code for the manuscript analyses and figures are openly available at https://github.com/rokitalab/clk1-splicing.

##### GTEX non-tumor tissue control RNA-Seq

We utilized OpenPedCan^16^ release v13 processed RNA-Seq data from the Genotype Tissue Expression (GTEx) project which had harmonized gene symbols to GENCODE v39 using the custom script at: https://github.com/d3b-center/D3b-DGD-Collaboration/blob/main/scripts/update_gene_symbols.py.

##### CLK1 morpholino RNA-Seq

RNA-sequencing data from the *CLK1* morpholino experiment has been deposited in GSE273841.

##### Merged primary and summary data

Merged primary matrices and summary files utilized in this manuscript were derived from are openly accessible via the download script in the https://github.com/rokitalab/clk1-splicing repository. To compare RNA-Seq from *CLK1* exon 4 morpholino-treated cells vs control morpholino-treated cells, we ran rMATs with three biological replicates for each condition `--b1 – b2`. This paired mode analysis calculated ΔPSI, p-values, and FDR statistics for each splice event.

### EXPERIMENTAL MODEL AND STUDY PARTICIPANT DETAILS

#### Study participants

Study participants include pediatric brain tumor patients whose genomic data was deposited into, and obtained from, the OpenPedCan^16^ project.

#### Patient-derived cell line models

The high-grade glioma patient-derived cell lines 7316–1736 and 7316–1739 were obtained by CBTN request and the KNS-42 cell line was obtained commercially as noted in the Key Resources table.

### METHOD DETAILS

#### Primary data analyses

Somatic primary workflows were implemented by the Kids First Data Resource Center as described in the Open Pediatric Brain Tumor Atlas (OpenPBTA)^43^ and OpenPedCan^16^ projects. The code for these workflows, including RNA-seq quantification, fusion identification, RNA splicing, and SNV, INDEL, CNV, SV calling, can be found at https://github.com/d3b-center/OpenPedCan-workflows. Sample-level data can be found through the Kids First Portal at https://kidsfirstdrc.org/.

#### *CLK1* exon 4 related visualizations and correlations

To visualize the *CLK1* exon 4 splice event, we utilized the R package *ggsashimi*^44^. We correlated *CLK1* exon 4 PSI values with *CLK1–201* or *CLK1* TPM. We computed Pearson correlation coefficients and p-values of this plot using the R package *ggpubr*^45^. High *CLK1* exon 4 inclusion tumors were defined as those with PSI values above the 75th percentile, while low SBI samples were those with PSI values below the 25th percentile comparing across all samples.

#### Protein Visualizations

Protein visualizations were obtained from the PhosphoSitePlus web portal^46^, emphasizing protein domains, residue numbers, and sites of phosphorylation binding.

#### Cell Culture

The pediatric HGG cell line KNS-42 was cultured in DMEM-F12 (GIBCO, 11320033) supplemented with 10% FBS (GIBCO, 26140079), 2 mmol/L L-glutamine (GIBCO, 25030081), and 1X penicillin/streptomycin (GIBCO, 15140122) at 37°C and 5% CO_2_. The cell line was authenticated by Guardian Forensic Sciences (Abington, PA) using the GenePrint 24 (Promega, B1870) short tandem repeat kit. Cells tested negative for mycoplasma using the EZ-PCR Mycoplasma Detection Kit (Biological Industries, 20-700-20) and were used for a maximum of 12 passages post thaw.

#### Morpholino Treatments

A Vivo-Morpholino ACTCTTCTGGAAACGTCAAGTGGGC (Gene Tools, LLC) targeting the intron 3-exon 4 splice junction was used to skip exon 4 in *CLK1*. Cells were treated with 1, 5, and 10 μM concentrations of *CLK1* morpholino and 10 μM of Control morpholino. 48 hours post-treatment, cells were harvested for PCR and immunoblots.

#### RNA Extraction and Quantitative Real-time PCR (qRT-PCR)

Total RNA was isolated and treated with DNAse using the Maxwell RSC simplyRNA Cells kit (Promega, AS1390) with the Maxwell RSC48 Instrument (Promega) per the manufacturer’s instructions. Next, 2 μg of RNA were reverse-transcribed using SuperScript IV (Invitrogen, 18090010). Primers used for *CLK1* mRNA transcript quantification are listed in Table S5H. qRT-PCR was performed using PowerSYBR Green PCR Master Mix (Invitrogen, 4367659) on an Applied Biosystems Viia7 machine. The amplification was performed using the following settings: denaturation at 95°C for 10 min, followed by 40 cycles of denaturation at 95°C for 15 s and annealing at 60°C for 1 min. The comparative cycle threshold (CT) method was applied to quantify the expression levels of *CLK1*. The fold change of gene expression was calculated by the equation 2ΔΔCT, with *HPRT* (Thermo Fisher, 4453320, assay ID: Hs02800695_m1) used as the housekeeping gene.

#### Protein Extraction

Cultured cells were washed once in chilled D-PBS (pH 7.4) and lysed in RIPA buffer containing 50 mM Tris-HCl, pH 7.4, NP-40 (1%), deoxycholate (0.25%), 150 mM NaCl, 1 mM EDTA pH 8.0, 1x protease and phosphatase inhibitor cocktail (Pierce Halt Inhibitor Cocktail, Thermo Fisher Scientific, 78446), and SDS (0.1%). Total protein in the lysate was estimated by the DC Protein assay (BioRad Laboratories, 5000111).

#### Detection of Proteins Using Immunoblot Analysis

70 μg of total protein were mixed with 5X SDS loading dye (Biorad, 161–0374) and resolved on 10% SDS-polyacrylamide gel. The protein was transferred onto a PVDF membrane (Immobilin-P, Millipore, IPVH00010) and probed with α-CLK1 mouse monoclonal primary antibody (Santa Cruz, sc-515897) and HRP-conjugated secondary antibody (Cell Signaling Technology, 7076S). Bands were detected using enhanced chemiluminescence (Millipore, WBKLS0500) and captured by a Chemiluminescence imager (GE Healthcare). β-actin was used as the loading control and probed with α-β-actin rabbit monoclonal antibody (Cell Signaling Technology, 12262S).

#### Cell Viability Assay

Cell viability was measured using the CellTitre-Glo (CTG) luminescent cell viability assay (Promega, G7570). Cells were seeded in white 96-well flat-bottom plates at a density of 24,000 cells per well and treated the following day with either 7.5 μM control or CLK1 exon 4 targeted morpholino. Luminescence was measured using a Biotek Synergy 2 plate reader at 24, 48, 72, and 96 hours.

#### pan-DYRK/CLK1 inhibitor Cirtuvivint (SM08502) experiments

The KNS-42 cell line was cultured in DMEM-F12 (Gibco, 11330032) supplemented with 10% FBS (Corning, MT3501CV, lot 003322001) and additional L-glutamine (Thermo Fisher, 25030081) to a final concentration of 4.5 mM. Dissociation was performed with Trypsin-EDTA (0.05%, Thermo 25300054) and counted on a DeNovix Cell Drop cell counter.

For growth kinetics, 10,000 (3 day assay) or 6,000 (6 day assay) cells were plated per well into a 96-well plate (Greiner Bio-One, 655098) in a 200 uL total volume per well. Plates were placed into an Incucyte SX5 device and scanned every 2 hours for several days to measure growth via a mask designed uniquely for this cell type. At the end point of the assay, cell viability was analyzed with CellTiter Glo 2.0 reagent (Promega, G9242) by replacing half the media with reagent and reading on a Promega GloMax device.

Cirtuvivint (MedChem Express, HY-137435) was resuspended in 100% DMSO (Sigma, D2650–5X5ML) to 1 mM and stored in aliquots at −80 C. Dosing was optimized via serial dilution at a range of 20 uM to 0.02 uM against a vehicle control equivalent to the highest dosing of drug. Cells were plated and at 24 hours, 100 uL of media were removed from each well and replaced with drug media for a final dose range of 0.01, 0.05, 0.5, 0.1, 1, 5, and 10 uM. Cells were untouched for 3 days total while growth was monitored via Incucyte.

### QUANTIFICATION AND STATISTICAL ANALYSIS

#### Splicing identification and quantification

To detect alternative splicing, we ran rMATS turbo (v. 4.1.0)^14^ with GENCODE v39 GFF annotations, as described by the Kids First RNA-Seq workflow (https://github.com/d3b-center/OpenPedCan-workflows). We filtered for alternative splicing events with ≥ 10 junction read counts. These results were then used for all downstream processing throughout the manuscript.

#### Splicing burden index (SBI) calculation

The following describes the SBI calculation used in the manuscript.

Let X be the list of all samples, where Xᵢ_j_ represents the j-th item in the i-th sample.

Let n be the number of items in each sample.

Let SE be the splice event of interest.

Let SEᵢ be the number of splice events in the i-th sample.

Let mean_SE_ be the mean of the splice event across all samples.

Let σ_SE_ be the standard deviation of the splice event across all samples.

Let SBI be the proportion of splice events that have z-scores > |2| out of the total number of splice events in a particular sample.

Then the equation for SBI is:

$$SBI_{i}=\frac{\sum_{j=1}^{n}I\left(\left|\frac{X_{i,j}-mean_{SE}}{\sigma_{SE}}\right|>|2|\right)}{n\cdot len(X)},$$

*where* i = 1 to len(X) and j = 1 to n

We compared PSI values of each primary tumor against all other tumors in the cohort. We first computed mean and standard deviation metrics for each alternative splicing event observed in at least one sample. Then for each sample in each group or histology, we identified the proportion of genes that underwent aberrant splicing as defined by a z-score > |2| across the entire transcriptome that undergoes alternative splicing.

#### Consensus clustering

We first preprocessed the splicing PSI matrix, restricting it to one splice event per gene by choosing the splice event with the maximum PSI value in a given gene. To reduce the dimensionality of the input matrix, we applied a feature selection using Hartigans’ dip test^47^. This test identifies dips in the distribution of input features and selects features that have a bi- or multi-modal distribution across the input samples. These “dips” in the distribution may correspond to differences within underlying clinical variables of interest. A total of 6999 features passed the test for multi-modality (p-value < 0.05) and were used for downstream clustering. Next, we applied all combinations of the following clustering algorithms (PAM, K-means, and Hierarchical) and distance methods (Pearson, Spearman, Euclidean, Manhattan, Binary, Maximum, Canberra, and Minkowski) available in the R package *ConsensusClusterPlus*^48^. For each combination, we evaluated a minimum *k* value of 2 and a maximum k value of 15. This resulted in a total of 272 clustering solutions corresponding to the different input combinations.

To identify the optimal clustering solution, we first evaluated the cluster performance using the R package *fpc*^49^. Using a given input data matrix and clustering solution, the function `fpc::clusterstats` computes the metrics silhouette width, entropy, purity, and Dunn index that represent separation between different clusters and closeness of data points within a cluster. The silhouette score defines the compactness of individual clusters (intra-cluster distance) and separation amongst clusters (inter-cluster distance) to measure an overall representative score. The entropy and purity evaluate the stability of the cluster. The higher the purity, the more stable the cluster is and the smaller the entropy, the better the clustering performance. The Dunn index is the ratio of the smallest inter-cluster distance and the largest intra-cluster distance. A higher Dunn Index will indicate compact, well-separated clusters, while a lower index will indicate less compact or less well-separated clusters. We used the R package *COINr* to assign weights to each metric and compute a composite score representing the overall “cluster quality”. The composite score was calculated by assigning a directional and weighted scoring mechanism. Ranks were assigned to each evaluated combination, with the highest composite score or cluster quality being assigned a rank of 1, 2, etc. For our dataset, the highest cluster quality, i.e. top ranking method, was the combination of PAM clustering algorithm with Binary distance measure and 12 clusters.

#### Clustering-based differential expression or pathway enrichment

We identified differentially expressed genes per cluster of interest and conducted pre-ranked pathway enrichment using *limma*^50^, *fgsea*^51^, and *GSVA*^52^ on those genes. We interrogated KEGG spliceosome and HALLMARK cancer pathways. We visualized these clusters using the R package *pheatmap*^53^ labeling rows with histology and calculated cluster information.

#### Differential expression and visualization

Differential expression was performed based on a model using the negative binomial distribution, a method employed by the R package *DeSeq2*^54^. Those differential genes that had a p-value < 0.05 were deemed as significantly up or down-regulated. Volcano plots were generated by the *EnhancedVolcano* R package. Bar plots were generated using the R package *ggplot2*^55^. Note: differential expression analyses were limited to stranded-only RNA-seq samples in order to limit batch effects.

#### Identification of recurrent functional differential splicing variants in pediatric HGGs

To identify differential or aberrant alternative splicing events, we assessed the percent spliced in (PSI) value of each splice event relative to the median PSI value of splice event across all samples. Splicing events with a ΔPSI exceeding |2| z-scores from the median PSI value were classified as differential or aberrant. For these events, we computed average ΔPSIs and generated bed files for each mis-spliced exon event. We then obtained bed files of known functional annotations as defined by Uniprot release 2024_03^24^ from UCSC Genome Browser web server. We ran bedtools v2.30^56^ to find the overlap between mis-spliced exons and functional features using the command `bedtools intersect -wo -à. We then plotted summary data by functional category (disulfide bonding sites, localization signals, amino acid modifications, and other).

#### Upset R and Volcano plots

To visualize the intersections of multiple sets, we employed the UpSetR^57^ plot in R. The input data consisted of differential and recurrent splicing events, if it was > 2 z-scores from the meanPSI and 2% of the histology-specific cohort. Volcano plots were generated by the *EnhancedVolcano* R package.

#### Splicing burden index and tumor mutation burden correlations

We identified samples with available data for both SBI (RNA-Seq) and WGS or WXS tumor mutation burden (TMB) from OpenPedCan^16^. Using the R package *ggscatter*, we performed a Pearson correlation analysis to examine the relationship between SBI and TMB. To ensure robustness, we repeated this analysis after excluding hyper-mutated samples (defined as those with TMB ≥ 10). Subsequently, we compared the distribution of TMB between high SBI and low SBI tumor samples using the Wilcoxon rank-sum test. High SBI samples were defined as those with SBI values above the 75th percentile, while low SBI samples were those with SBI values below the 25th percentile. The analyses were conducted across all samples and further stratified according to `plot_group`, as specified in the histologies clinical file.

#### Pathway over-representation analysis (ORA) and gene set variation analysis (GSVA)

We conducted over-representation analysis (ORA) using the R package clusterProfiler^58^ and pathway data from the msigdbr package^59^, including “CP:KEGG”, “CP:BIOCARTA”, “CP:HALLMARK”, and “TFT:GTRD.” After inputting the genes of interest (eg. differentially spliced), we applied a p-value cutoff of 0.05 and used the Benjamini-Hochberg (BH) method for p-value adjustment. For visualization of the over-represented pathways, we employed the ènrichplot::dotplot()` function, displaying the gene ratio and the count of genes in each pathway.

To perform Gene set variation analysis (GSVA) we utilized the R packages `GSVÀ and `msigdbr`. Expression data for our samples, sourced from OpenPedCan v13^16^, were used to compute gene-set enrichment scores. Genes with zero variance were excluded from the analysis. We then assessed enrichment in Hallmark, KEGG, and custom pathways from Knijnenburg et al^60^. Gaussian-distributed scores were calculated using *gsvaParam* function in R. The results were visualized using heatmaps of GSVA scores, generated with the R packages *ComplexHeatmap* and *circlize*.

#### Oxford Nanopore Technologies (ONT) Targeted Long-Read RNA-Sequencing

We designed primers to bind to exons present in all isoforms of *CLK1* to ensure full coverage of all alternative splicing events. 5 ng of cDNA were amplified with LongAmp Taq 2X Master Mix (M0287S, New England Biolabs) for 25 cycles. The resulting amplicons were subjected to amplicon-seq (SQKNBD112.24, ONT) library preparation, loaded into a Spot-ON flow cell R9 Version (FLO-MIN112, ONT), and sequenced in a MinION Mk1C device (ONT) until at least 1,000 reads per sample were obtained. Results were aligned using Minimap2 version 2.24-r1122 and visualized in IGV version 2.12.3.

#### DepMap and CRISPR dependency analyses

Datasets comprising gene transcript expression, cell line information, and CRISPR dependency scores were downloaded from DepMap (version 24Q2). The expression of *CLK1* ENST00000321356 (exon 4 containing transcript) was categorized into high and low TPM expression, defined by values above the 75th quantile and below the 25th quantile, respectively. CRISPR dependency scores were plotted on the y-axis, and Wilcoxon tests were conducted to compare high versus low TPM expression groups. These were stratified for each cell line type. Additionally, CRISPR dependency scores for all CNS/brain cell lines were plotted, with KNS-42 highlighted in red. For the Childhood Cancer Model Atlas CRISPR dependency analyses, we acquired data from the Childhood Cancer Model Atlas^33^. We plotted CRISPR dependency scores (z) on the y-axis for each gene in CBTN pediatric HGG cell lines, either as median scores or stratified by individual patients with genes of interest highlighted.

#### Proteogenomic analysis

Pediatric proteomics, phosphoproteomics, and RNA data were obtained from the Clinical Proteomic Tumor Analysis Consortium (CPTAC) via the ProTrack: Pediatric Brain Tumor open-source web portal. Data and z-scores were computed using the methods described by Petralia et al^22^.

## Supplementary Material

Supplement 1

Supplement 2

Supplement 3

Supplement 4

Supplement 5

1

## Figures and Tables

**Table:** KEY RESOURCES TABLE

REAGENT or RESOURCE	SOURCE	IDENTIFIER
**Antibodies**		
α-CLK1 (F-12) mouse mAb	Santa Cruz	sc-515897
α-β-Actin (8H10D10) Mouse mAb (HRP Conjugate)	Cell Signaling Technology	12262S
Anti-mouse IgG, HRP-linked Antibody	Cell Signaling Technology	7076S
**Drugs**		
DMSO	Sigma	D2650-5X5ML
Cirtuvivint (SM08502)	MedChem Express	HY-13743
**Critical commercial assays**		
GenePrint 24	Promega	B1870
EZ-PCR Mycoplasma Detection Kit	Biological Industries	20-700-20
Maxwell RSC simplyRNA Cells Kit	Promega	AS1390
SuperScript IV	Invitrogen	18090010
CellTitre-Glo luminescent cell viability assay	Promega	G7570
**Deposited data**		
PBTA raw WGS, WXS, Panel, RNA-Seq	dbGAP	phs002517.v2.p2
PBTA harmonized WGS, WXS, Panel, RNA-Seq	OpenPedCan^16^	https://github.com/d3b-center/OpenPedCan-workflows
PBTA merged summary files and downstream analyses	This project	10.5281/zenodo.13362856
Morpholino treated KNS42 RNA-seq	This project	GSE273841
**Experimental models: Cell lines**		
PBTA patient-derived cell lines 7316-1763 and 7316-1769	Children’s Brain Tumor Network	Children’s Brain Tumor Network
KNS-42 human glioma cell line	Accegen	ABC-TC0532
**Oligonucleotides**		
CLK1 intron 3-exon 4 splice junction targeting morpholino - ACTCTTCTGGAAACGTCAAGTGGGC	Gene Tools, LLC, This Project	
CLK1 Ex3-Ex4 (inclusion of Exon4) F- GGACATCGCCAAAGAGACCA	Integrated DNA Technologies IDT, This Project	
CLK1 Ex3-Ex4 (inclusion of Exon4) R- TCCTTCGGTGACTCTTCCCA	Integrated DNA Technologies IDT, This Project	
CLK1 Ex3-Ex5 (inclusion of Exon4) F- ATCGCCAAAGAGACCATGAAAG	Integrated DNA Technologies IDT, This Project	
CLK1 Ex3-Ex5 (inclusion of Exon4) R- GTATCAACAATTTCATCCCATGTGA	Integrated DNA Technologies IDT, This Project	
CLK1 Ex3-Ex5 (inclusion of Exon4) F- CCATGAAAGCCGGTATCAGAAC	Integrated DNA Technologies IDT, This Project	
CLK1 Ex3-Ex5 (inclusion of Exon4) R- ACCTAAAGTATCAACAAIIICATCCCA	Integrated DNA Technologies IDT, This Project	
**Software and algorithms**		
Replicate Multivariate Analysis of Transcript Splicing (rMATS)	https://github.com/Xinglab/rmats-turbo	rMATS-turbo: an efficient and flexible computational tool for alternative splicing analysis of large-scale RNA-seq data^14^
ggsashami	https://github.com/guigolab/ggsashimi	ggsashimi: Sashimi plot revised for browser- and annotation-independent splicing visualization^44^
OpenPedCan analysis repository v2.1.0 (data release v13)	OpenPedCan^16^	https://github.com/d3b-center/OpenPedCan-analysis
Project repository	This Project	https://github.com/rokitalab/clk1-splicing

## Highlights

- Splice variation across pediatric CNS tumors from 1,415 patients is heterogeneous
- In high-grade gliomas, 11,940 splice events in 5,368 genes contain functional sites
- *CLK1* exon 4 inclusion adds two phosphorylation binding sites and drives expression
- *CLK1* splicing impacts cancer genes, phenotypes, and is therapeutically targetable
