# Characterization of aberrant splicing in pediatric central nervous system tumors reveals *CLK1* as a candidate oncogenic dependency

**DOI:** 10.1101/2024.08.03.606419

**Published:** 2025-08-19

**Authors:** Ammar S. Naqvi, Patricia J. Sullivan, Ryan J. Corbett, Priyanka Sehgal, Karina L. Conkrite, Komal S. Rathi, Brian M. Ennis, Katharina E Hayer, Bo Zhang, Miguel A. Brown, Daniel P. Miller, Alex Sickler, Adam A. Kraya, Kaleem L. Coleman, Joseph M. Dybas, Zhuangzhuang Geng, Christopher Blackden, Shehbeel Arif, Antonia Chroni, Aditya Lahiri, Madison L. Hollawell, Phillip B. Storm, Dalia Haydar, Jessica B. Foster, Mateusz Koptyra, Peter J. Madsen, Sharon J. Diskin, Andrei Thomas-Tikhonenko, Adam C. Resnick, Jo Lynne Rokita

**Affiliations:** 1 Center for Data-Driven Discovery in Biomedicine, Children’s Hospital of Philadelphia, Philadelphia, PA, USA; 2 Division of Neurosurgery, Children’s Hospital of Philadelphia, Philadelphia, PA, USA; 3 Center for Cancer and Immunology Research, Children’s National Hospital, Washington, D.C., USA; 4 Division of Cancer Pathobiology, Children’s Hospital of Philadelphia, Philadelphia, PA, USA; 5 Division of Oncology and Center for Childhood Cancer Research, Children’s Hospital of Philadelphia, Philadelphia, PA; 6 Department of Biomedical and Health Informatics, Children’s Hospital of Philadelphia, Philadelphia, PA, USA; 7 Department of Pediatrics, The George Washington University School of Medicine and Health Sciences, Washington, D.C., USA; 8 Department of Pediatrics, University of Pennsylvania, Philadelphia, PA, USA; 9 Department of Pathology and Laboratory Medicine, University of Pennsylvania, Philadelphia, PA, USA

**Keywords:** aberrant splicing, pediatric brain tumors, *CLK1*, oncogenic dependency, therapeutic targeting

## Abstract

**Background:**

Pediatric brain cancer is the leading cause of disease-related mortality in children, yet many aggressive tumors lack effective therapies. Aberrant RNA splicing is a hallmark of cancer, but its role in pediatric central nervous system (CNS) tumors remains underexplored.

**Methods:**

We analyzed 752 primary pediatric brain tumors spanning histologies and molecular subtypes to characterize aberrant splicing. We developed the *Splicing Burden Index (SBI)* as a sample-level metric and performed hierarchical clustering of variable splice events. Cluster-specific events were annotated for overlap with functional protein domains. We conducted *in vitro* experiments to assess the role of the splicing regulator *CDC-like kinase 1 (CLK1)*.

**Results:**

Clustering identified 11 splicing-defined groups, several enriched for distinct tumor types, though heterogeneity was observed across histologies. Cluster 6, with the poorest event-free survival, comprised multiple histologies and contained 39,528 aberrant splice events across 10,412 genes, including 8,369 single exon (SE) events overlapping known functional protein domains. *CLK1* expression was most positively correlated with SE SBI in Cluster 6, and inclusion of its exon 4 (active kinase isoform) was widespread and associated with inferior event-free survival. Pharmacologic inhibition of *CLK1* with cirtuvivint suppressed viability and proliferation in KNS-42 cells, while morpholinos targeting exon 4 reduced *CLK1* RNA and protein abundance, impaired growth, and induced splicing and expression changes in cancer-related genes.

**Conclusions:**

This study maps splicing dysregulation in pediatric brain tumors, defines biologically- and clinically-relevant splicing-defined subgroups, and identifies *CLK1* as a regulator of oncogenic splicing programs. These findings support therapeutic targeting of splicing kinases in high-risk pediatric CNS tumors.

## Introduction

Pediatric brain cancer is the number one cause of disease-related death in children^1^. Furthermore, pediatric high-grade gliomas (HGGs) are largely resistant to chemotherapy and are often surgically unresectable, making them exceptionally challenging^2^. Despite decades of clinical trials in pediatrics, patients with certain tumor types such as diffuse intrinsic pontine glioma (DIPG) or diffuse midline glioma (DMG) will succumb to their disease, with a median overall survival of 11 months in patients^3^. Even with optimal multimodal therapy, median overall survival for non-DMG patients with a HGG is 14–20 months^4^.

Within the last decade, advances in surgical biopsy, autopsy donation, and sequencing have enabled genomic characterization of pediatric HGGs, revealing recurrent somatic drivers including mutations, fusions, and copy number alterations. However, the role of alternative splicing, a key post-transcriptional regulatory mechanism, remains underexplored in these tumors. Previous studies have identified rare, mutually exclusive mutations in spliceosome-associated factors such as *SF3B1* and *SF3B2* in pediatric HGGs, which disrupt cellular processes involved in maintenance of DNA replication, genome integrity, or transcriptional fidelity^5,6^. More recently, Siddaway, et. al. describe alternative aberrant splicing as a mechanism of oncogenic pathway activation in pediatric HGGs, including those in the RAS/MAPK pathway, leading to worse patient survival^7^. Furthermore, an alternatively spliced neuronal cell adhesion molecule (NRCAM) but not its canonical isoform was found to be essential for pHGG cell linederived xenograft growth *in vivo*^8^.

Alternative splicing plays essential roles in gene regulation, expanding proteomic diversity, modulating transcript stability, and controlling protein localization, folding, and function^9,10^. The mammalian brain exhibits the most complex and conserved alternative splicing programs of any tissue, and their disruption is associated with a variety of neurodevelopmental disorders and neurological diseases^11,12^. Splicing regulation is tightly governed by *trans*-acting RNA-binding proteins (RBPs), such as the Serine-rich Splicing Factors (SRSFs), which bind to regulatory sequences on preliminary mRNA transcripts. Disturbing that balance or their regulators can be detrimental to cellular functionality and result in oncogenic transformation^13,14^.

To date, however, comprehensive analysis of alternative splicing across pediatric CNS tumors has not been performed. While prior work has focused on HGGs, the broader role across diverse pediatric brain tumor types remains unknown. In this study, we perform a large-scale analysis of genome-wide alternative splicing across 752 pediatric CNS tumors encompassing a wide range of histologic and molecular subtypes. We uncover widespread splicing alterations that reveal unique splicing biology within and across histologies and/or molecular subtypes. Many of these events are predicted to disrupt known protein domains, potentially altering function. We identify and experimentally characterize *CLK1* as a candidate oncogenic dependency in pediatric CNS tumors and propose that targeting aberrant splicing and/or its resulting downstream proteins may offer additional therapeutic avenues for precision cancer therapy.

## Materials and Methods

### Study participants and patient-derived cell line models

Study participants include pediatric brain tumor patients whose genomic data were deposited into and obtained from the OpenPedCan^15^ project. Histologies include Atypical Teratoid Rhabdoid Tumor (ATRT, N = 56), choroid plexus tumor (CPT, N = 32), craniopharyngioma (CPG, N = 51), diffuse intrinsic pontine glioma or diffuse midline glioma (DIPG or DMG, N = 135), ependymoma (N = 110), germ cell tumor (N = 16), low-grade glioma (LGG, N = 337), medulloblastoma (MB, N = 200), meningioma (N = 29), mesenchymal tumor (N = 26), mixed neuronal-glial tumor (GNT, N = 112), neurofibroma plexiform (N = 12), non-neoplastic tumor (N = 45), other CNS embryonic tumor (N = 16), other high-grade glioma (other HGG, N = 190), schwannoma (N = 21), and other rare brain tumors (N = 27).

### Primary data analyses

Somatic primary workflows were implemented by the Kids First Data Resource Center as described in the Open Pediatric Brain Tumor Atlas (OpenPBTA)^16^ and OpenPedCan^15^ projects. The code for these workflows, including RNA-seq quantification, fusion identification, RNA splicing, and SNV, INDEL, CNV, SV calling, can be found at https://github.com/d3b-center/OpenPedCan-workflows. Sample-level data can be found through the Kids First Portal at https://kidsfirstdrc.org/. To detect alternative splicing, we ran rMATS turbo (v. 4.1.0)^17^ with GENCODE v39 GFF annotations on single samples, as described by the Kids First RNA-Seq workflow (https://github.com/d3b-center/OpenPedCan-workflows). We filtered for alternative splicing events with ≥ 10 junction read counts. To compare RNA-Seq from *CLK1* exon 4 morpholino-treated cells vs control morpholino-treated cells, we ran rMATs with three biological replicates for each condition ‘--b1 –b2’. This paired mode analysis calculated ΔPSI, p-values, and FDR statistics for each splice event. These results were then used for all downstream processing throughout the manuscript.

### Cell Culture

The high-grade glioma patient-derived cell lines 7316–1763 and 7316–1769 were obtained by CBTN request, and the KNS-42 cell line was obtained from Accegen (ABCTC0532). The pediatric HGG cell line KNS-42 was cultured in DMEM-F12 (GIBCO, 11320033) supplemented with 10% FBS (GIBCO, 26140079), 2 mmol/L L-glutamine (GIBCO, 25030081), and 1X penicillin/streptomycin (GIBCO, 15140122) at 37°C and 5% CO_2_. The cell line was authenticated by Guardian Forensic Sciences (Abington, PA) using the GenePrint 24 (Promega, B1870) short tandem repeat kit. Cells tested negative for mycoplasma using the EZ-PCR Mycoplasma Detection Kit (Biological Industries, 20-700-20) and were used for a maximum of 12 passages post thaw.

### Morpholino Treatments

A Vivo-Morpholino ACTCTTCTGGAAACGTCAAGTGGGC (Gene Tools, LLC) targeting the intron 3-exon 4 splice junction was used to skip exon 4 in *CLK1*. Cells were treated with 1, 5, and 10 μM concentrations of *CLK1* morpholino and 10 μM of Control morpholino. 48 hours post-treatment, cells were harvested for PCR and immunoblots.

### RNA Extraction and Quantitative Real-time PCR (qRT-PCR)

Total RNA was isolated and treated with DNAse using the Maxwell RSC simplyRNA Cells kit (Promega, AS1390) with the Maxwell RSC48 Instrument (Promega) per the manufacturer’s instructions. Next, 2 μg of RNA were reverse-transcribed using SuperScript IV (Invitrogen, 18090010). Primers used for *CLK1* mRNA transcript quantification are listed in TableS6J. qRT-PCR was performed using PowerSYBR Green PCR Master Mix (Invitrogen, 4367659) on an Applied Biosystems Viia7 machine. The amplification was performed using the following settings: denaturation at 95°C for 10 min, followed by 40 cycles of denaturation at 95°C for 15 s and annealing at 60°C for 1 min. The comparative cycle threshold (CT) method was applied to quantify the expression levels of *CLK1*. The fold change of gene expression was calculated by the equation 2ΔΔCT, with *HPRT* (Thermo Fisher, 4453320, assay ID: Hs02800695_m1) used as the housekeeping gene.

### Protein Extraction

Cultured cells were washed once in chilled D-PBS (pH 7.4) and lysed in RIPA buffer containing 50 mM Tris HCl, pH 7.4, NP 40 (1%), deoxycholate (0.25%), 150 mM NaCl, 1 mM EDTA pH 8.0, 1x protease and phosphatase inhibitor cocktail (Pierce Halt Inhibitor Cocktail, Thermo Fisher Scientific, 78446), and SDS (0.1%). Total protein in the lysate was estimated by the DC Protein assay (BioRad Laboratories, 5000111).

### Detection of Proteins Using Immunoblot Analysis

70 μg of total protein were mixed with 5X SDS loading dye (Biorad, 161–0374) and resolved on 10% SDS polyacrylamide gel. The protein was transferred onto a PVDF membrane (Immobilin-P, Millipore, IPVH00010) and probed with α-CLK1 mouse monoclonal primary antibody (Santa Cruz, sc-515897) and HRP conjugated secondary antibody (Cell Signaling Technology, 7076S). Bands were detected using enhanced chemiluminescence (Millipore, WBKLS0500) and captured by a Chemiluminescence imager (GE Healthcare). β-actin was used as the loading control and probed with α-β-actin rabbit monoclonal antibody (Cell Signaling Technology, 12262S).

### pan-DYRK/CLK1 inhibitor Cirtuvivint (SM08502) experiments

The KNS-42 cell line was cultured in DMEM-F12 (Gibco, 11330032) supplemented with 10% FBS (Corning, MT3501CV, lot 003322001) and additional L-glutamine (Thermo Fisher, 25030081) to a final concentration of 4.5 mM. Dissociation was performed with Trypsin-EDTA (0.05%, Thermo 25300054) and counted on a DeNovix Cell Drop cell counter.

For growth kinetics, 10,000 (3 day assay) or 6,000 (6 day assay) cells were plated per well into a 96-well plate (Greiner Bio-One, 655098) in a 200 uL total volume per well. Plates were placed into an Incucyte SX5 device and scanned every 2 hours for several days to measure growth via a mask designed uniquely for this cell type. At the end point of the assay, cell viability was analyzed with CellTiter Glo 2.0 reagent (Promega, G9242) by replacing half the media with reagent and reading on a Promega GloMax device.

Cirtuvivint (MedChem Express, HY-137435) was resuspended in 100% DMSO (Sigma, D2650–5X5ML) to 1 mM and stored in aliquots at −80 C. Dosing was optimized via serial dilution at a range of 20 uM to 0.02 uM against a vehicle control equivalent to the highest dosing of drug. Cells were plated and at 24 hours, 100 uL of media were removed from each well and replaced with drug media for a final dose range of 0.01, 0.05, 0.5, 0.1, 1, 5, and 10 uM. Cells were untouched for 3 days total while growth was monitored via Incucyte.

### Cell Viability Assay

Cell viability was measured using the CellTitre-Glo (CTG) luminescent cell viability assay (Promega, G7570). Cells were seeded in white 96-well flat-bottom plates at a density of 24,000 cells per well and treated the following day with either 7.5 μM control or CLK1 exon 4 targeted morpholino. Luminescence was measured using a Biotek Synergy 2 plate reader at 24, 48, 72, and 96 hours.

## Results

### Pediatric brain tumors display heterogeneous global patterns of aberrant splicing

**Figure 1:**
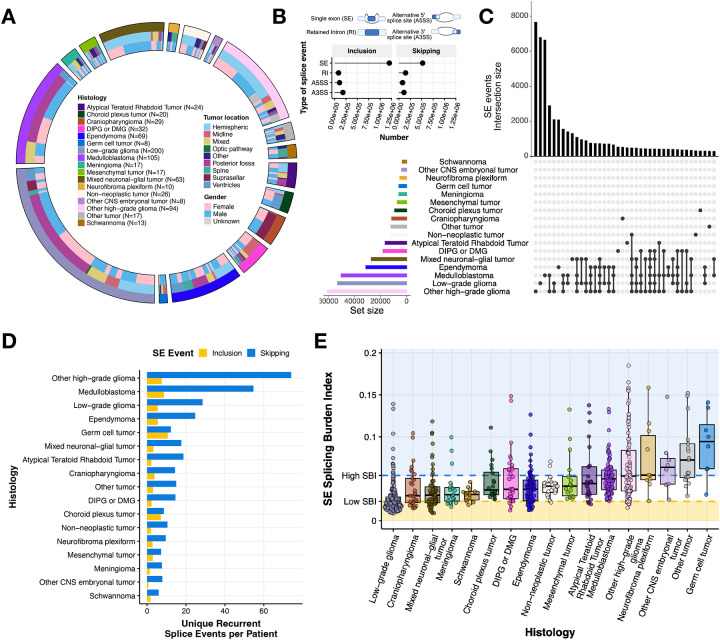
Pediatric brain tumors display heterogeneous global patterns of aberrant splicing. **(A)** Circos plot of CNS tumors used in this study, categorized by histology, tumor location, and reported gender. Non-neoplastic tumors consist of benign tumors and/or cysts. **(B)** Lollipop plot illustrating the total number of recurrent differential splicing events across the cohort, classified by splicing type (SE: single exon, RI: retained intron, A3SS: alternative 3’ splice site, A5SS: alternative 5’ splice site). **(C)** UpsetR plot of recurrent differential SE splicing events (N ≥ 2 of samples within a histology). **(D)** Barplots of the number of histology-specific recurrent SE events per patient. Histologies are reverse-ordered by the total number of unique events (skipping + inclusion). **(E)** Distribution plots of SE splicing burden index (SBI) by histology. Shaded regions represent high (blue; ≥ Quartile 3) and low (yellow; ≤ Quartile 1) SE SBI groups.

To investigate alternative splicing in pediatric brain tumors, we analyzed stranded total RNA-Seq data for 752 diagnostic tumors from the Open Pediatric Cancer (OpenPedCan) project^15^ (Figure 1A). Demographic and clinical data for each patient and tumor in this study are available in Table S1. We quantified splicing events at single-sample resolution using percentspliced-in (PSI) values derived from replicate Multivariate Analysis of Transcript splicing (rMATS)^17,18^ to identify skipped exon (SE), alternative 5’ splice site (A5SS), alternative 3’ splice site (A3SS), and retained intron (RI) events.

Due to the diverse biological and molecular drivers of these CNS cancers, we hypothesized that we might observe distinct splicing patterns across histologies and/or molecular subtypes. Among recurrent (N ≥ 2) differential splice events, SE events were the most frequently observed (Figure 1B), consistent with a previous report in pediatric HGGs^7^. We then explored whether these SE events were histology-specific or shared events across tumor types. Indeed, we observed both shared and histology-specific SE events (Figure 1C), with medulloblastoma (MB), low-grade glioma (LGG), and non-DIPG/DMG high-grade glioma (other HGG) tumors exhibiting the highest number of unique recurrent SE events. This pattern remained consistent after normalizing by the number of patients within each histology (Figure 1D). A complete list of unique events per histology is reported in Table S2A.

To enable cross-sample comparisons of alternative splicing, we devised the splicing burden index (SBI): a metric that quantifies the proportion of differential alternative splicing (AS) events in a sample (Online Methods). SBI is calculated using a single event type, with the following analysis focused on SE events. Across the cohort, the median SBI was 0.0356 (3.56%). Among tumor types, LGGs had the lowest median SBI at 2.05%, while germ cell tumors (GCTs) had the highest at 9.43% (Figure 1E). SBI also revealed variability within tumor histologies. Tumors with high SBI variance (> 3rd quartile variance) indicate more heterogeneous tumors, including neurofibroma plexiform, other HGGs, GCTs, and other rare tumors. We performed a similar analysis using other splice types (Alternative 5’ Splice Site [A5SS], Alternative 5’ Splice Site [A3SS], and Retained Intron [RI]) and observed that LGGs continued to exhibit the lowest median SBI, while other rare tumors and GCTs maintained the highest median SBI (Figures S1A–C). Despite a relatively low splicing burden across all histologies, the observed variability highlights the nuanced tumor-specific and intra-tumoral heterogeneity of splicing programs in these CNS tumors.

We hypothesized that tumors with a low tumor mutation burden (TMB) might have a higher splicing burden index as an alternate mechanism driving tumorigenesis. However, we observed the opposite: a weak but significant positive correlation between TMB and SBI (Pearson’s R = 0.15, p-value = 8.2e-5; Figure S1D), which persisted after excluding hyper-mutant tumors (R = 0.15, p-value = 7.4e-5, Figure S1E).

### Splice events cluster pediatric brain tumor histologies and are associated with survival outcomes

**Figure 2:**
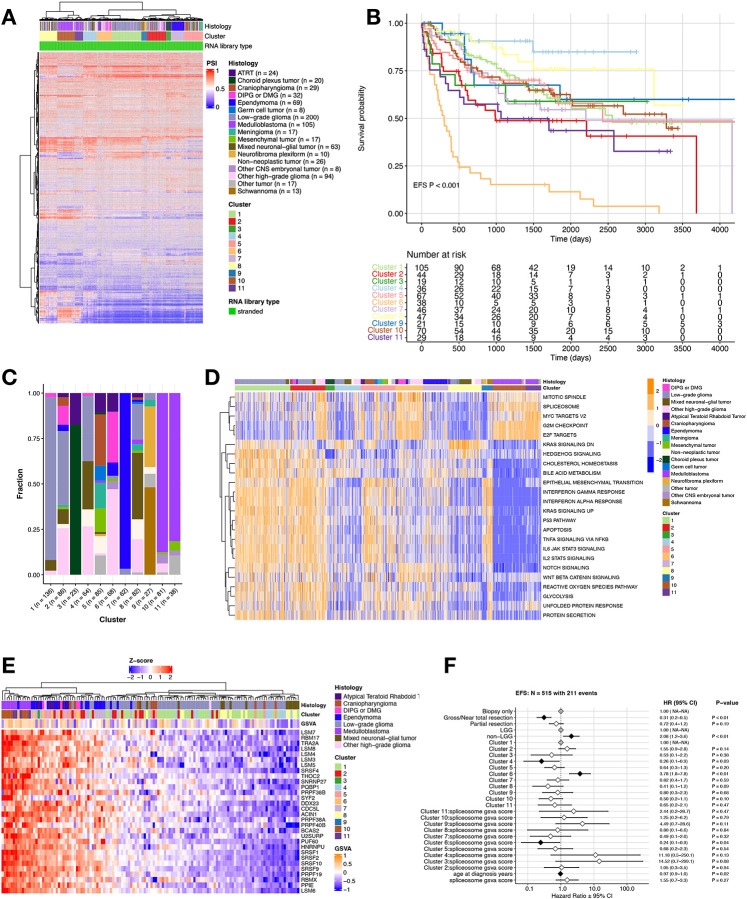
Splicing-based clustering reveals heterogeneous tumor groups with distinct histologies, pathway activities, and clinical outcomes. **(A)** Hierarchical clustering heatmap of PSI values for top 5,000 most variable SE splice events across tumors. **(B)** Kaplan-Meier curve of EFS for patients by cluster membership. **(C)** Stacked barplot showing histology sample membership fraction for each cluster. **(D)** Heatmap of top cancer-related pathways by cluster (GSVA scores represented by blue/orange color). **(E)** Heatmap of protein abundance for top 30 most variable proteins within the KEGG spliceosome pathway (GSVA scores denote KEGG spliceosome scores) **(F)** Forest plot of cox proportional hazards multivariate interaction EFS model for cluster and KEGG spliceosome GSVA score, including covariates for tumor resection and age at diagnosis. Black and white diamonds indicate statistically significant and not significant hazard ratios (HRs), respectively, with intervals denoting 95% confidence intervals. Gray diamonds indicate reference levels of factor covariates.

To assess whether CNS tumors exhibit shared splicing patterns, we performed hierarchical clustering of samples using PSI values from the top 5,000 most variable SE events across all primary tumors (N = 752). This analysis revealed 11 distinct clusters, each enriched for specific histologies and/or molecular subtypes (Figure 2A and S2A–E). For instance, Clusters 10 and 11 were enriched for MB, with Cluster 10 showing significant enrichment for Group 4 (OR = 80.5, p < 0.05) and Cluster 11 enriched for the SHH subtype (OR = 5.1, p < 0.05) (Figure S2B). These findings are consistent with prior work demonstrating that the MB subgroups WNT, SHH, Group 3, and Group 4 can be delineated based on splicing patterns^19^. Clusters 5 and 6 were significantly enriched for ATRT-MYC and ATRT-SHH subtypes, respectively (OR = 38.4 and 19.2, p < 0.05) (Figure S2C). HGGs, including DIPG/DMGs, exhibited the greatest splicing heterogeneity, with samples spanning 7 of the 11 identified clusters (Figure S2D–E). We examined event-free survival (EFS) across splicing-defined clusters and found that Cluster 6 was associated with the poorest EFS (p < 0.001) (Figure 2B). This cluster was predominantly comprised of HGGs, representing 75% of the group (Figure 2C), along with a mix of additional histologies. Clusters 4, 8, and 9 demonstrated the most favorable EFS (p < 0.05); Cluster 4 was significantly enriched for non-neoplastic tumors, other HGGs (including H3 wildtype and H3 G35 tumors), and mixed neuronal-glial tumors (GNT); Cluster 8 for non-neoplastic tumors and GNTs; and Cluster 9 for neurofibromatosis-associated (NF) tumors (Figure S2A). A full list of samples with associated cluster membership information is outlined in Table S2B.

To explore the functional impact of splicing alterations, we identified differentially expressed cancer-associated signaling pathways in each of the 11 clusters (Figure 2D). Strikingly, the spliceosome pathway was significantly upregulated in Cluster 6, the cluster with the worst EFS (Table S2C, Log_2_FC = 0.41, Bonf-adj p = 4.89e-13 vs. all other groups) and significantly downregulated in Clusters 4, 8, and 9, with significantly better EFS (Table S2, Bonf-adj p < 0.05). In addition, the epithelial mesenchymal transition (EMT) pathway was the most significantly differentially-expressed in Cluster 9 (Table S2, Bonf-adj p = 3.39e-9), enriched for NF tumors and consistent with a previous report demonstrating activated EMT in samples with NF-1 loss^20^. These results demonstrate that spliced genes can cluster into biologically meaningful groups, highlighting distinct pathways and gene targets within each cluster.

To evaluate whether upregulation of the KEGG spliceosome GSVA score translates into elevated protein expression, we investigated splicing factor protein levels by integrating gene expression and matched proteogenomic (N = 122) data from the Clinical Proteomic Tumor Analysis Consortium (CPTAC)^21^. We visualized protein (Figure 2E) and RNA (Figure S2F) expression of KEGG spliceosome pathway genes (Table S3A) alongside their corresponding GSVA scores and observed a significant positive correlation between protein abundance and KEGG spliceosome scores (R = 0.4, p = 2.6e-6; Figure S2G), suggesting that RNA expression can serve as a reliable readout for pathway activity.

We next assessed whether spliceosome activity, measured by GSVA score, was associated with survival outcomes across the cohort using a multivariate cox proportional hazards model adjusting for tumor resection, glioma grade group, age at diagnosis, and cluster. Favorable prognostic variables included tumor resection (HR = 0.31, p < 0.01) and Cluster 4 membership (HR = 0.26, p = 0.03), whereas non-LGG tumors (HR = 2.08, p < 0.01) and Cluster 6 membership (HR = 3.78, p < 0.01) were associated with worse EFS. Notably, we detected a significant interaction between spliceosome GSVA scores and Cluster 6, where higher spliceosome activity conferred a survival benefit (HR = 0.24, p = 0.04, Figure 2F). Given the prognostic effects of both spliceosome GSVA scores and SE SBI, we further tested their relationship using an interaction model, which revealed a significant synergistic effect (HR = 0.25, p < 0.01; Figure S2H). Together, these results suggest that elevated spliceosome activity, particularly in tumors with high SE burden, may partially offset the poor prognosis associated with aggressive subgroups such as Cluster 6.

### Widespread splicing alterations driven by expression changes in splicing factors

**Figure 3:**
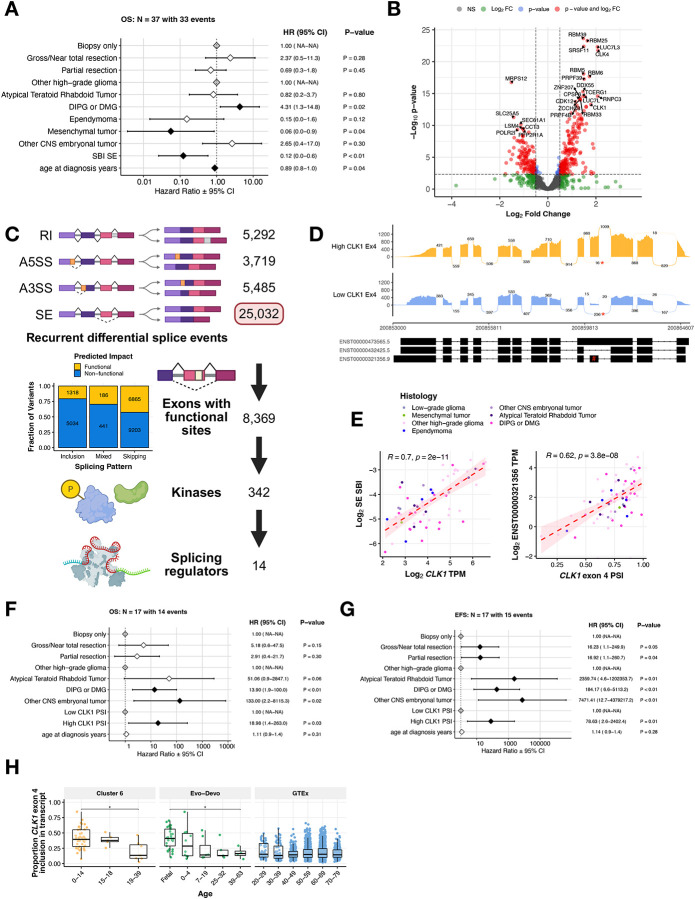
Functional splice event prioritization in Cluster 6 nominates *CLK1* Exon 4 inclusion as an oncogenic dependency. **(A)** Cox proportional hazards model forest plot of OS among patients in Cluster 6, including covariates for extent of tumor resection, histology group, SE SBI, and age at diagnosis. Black and white diamonds indicate statistically significant and not significant hazard ratios, respectively, with intervals denoting 95% confidence intervals. Gray diamonds indicate reference levels of factor covariates. **(B)** Volcano plot illustrating differentially-expressed splicing factor genes in Cluster 6 tumors with high SBI compared to those with low SBI (NS = not significant, FC = fold change, colored dots represent log_2_FC > |.5| and/or Benjamini and Hochberg (B-H) adjusted p-value < 0.05). **(C)** Workflow to prioritize candidate differential exon-level splicing events that alter UniProt-defined functional sites in Cluster 6 tumors. Stacked bar plots represent the fraction of exon inclusion, skipping, or mixed splicing events categorized by predicted impact. **(D)** Sashimi plot of two representative tumor samples with either high (BS_30VC3R2Q) or low (BS_FYP2B298) *CLK1* exon 4 inclusion. Reads supporting exon 4 skipping are marked with an asterisk (*), and exon 4 is indicated by a hash (#) on the transcript plot. **(E)** Scatter plot showing log_2_-single exon SBI versus log_2_-*CLK1* TPM and log_2_-*CLK1* ENST00000321356 transcript TPM versus log_2_-*CLK1* exon 4 PSI in Cluster 6 samples. Pearson’s R and p-value are shown. **(F)** Cox proportional hazards model forest plot of OS or **(G)** EFS among patients in Cluster 6, including covariates for extent of tumor resection, histology group, *CLK1* exon 4 PSI, and age at diagnosis. Black and white diamonds indicate statistically significant and not significant hazard ratios, respectively, with intervals denoting 95% confidence intervals. Gray diamonds indicate reference levels of factor covariates. **(H)** Distribution of *CLK1* exon 4 PSI across age categories in Cluster 6 samples, Evo-Devo tissues, and GTEx controls. All boxplots represent the 25th and 75th percentile and the bar represents the median.

Since Cluster 6 exhibited marked upregulation of spliceosome pathways and the poorest event-free survival, we selected it for further investigation. This cluster also showed the greatest variability in SBI scores, with the largest interquartile range and overall spread across all clusters (Figure S3A), suggesting that splicing burden may influence outcomes within this aggressive group. Consistent with the protective effect of higher GSVA scores observed in Figure 2F, a multivariate model stratifying GSVA scores by quartile confirmed that tumors in the highest (4th) quartile had significantly better EFS than those in the lowest (1st) quartile (HR = 0.18, p = 0.03; Figure S3B). Similarly, in a separate model, higher SE SBI scores were significantly associated with improved overall survival in Cluster 6 (HR = 0.12, p < 0.01, Figure 3A), a finding further supported by Kaplan–Meier analysis showing significantly better overall survival for tumors in the highest versus lowest SBI quartile tumors (p = 0.041; Figure S3C).

To investigate the mechanisms driving the widespread splicing alterations, we first assessed somatic alterations in Cluster 6. Consistent with the high proportion of HGGs present in Cluster 6, we observed frequent mutations in *TP53* (55%), *H3–3A* (49%), and *ATRX* (42%) (Figure S3D). Mutations in either HUGO spliceosome genes (Table S2B) or known splicing factor genes (Table S2C) were absent in Cluster 6 tumors and rare in the cohort overall (Tables S3D–E), suggesting alternative regulatory mechanisms as drivers of the splicing alterations that define Cluster 6. It has been previously shown that in the absence of splicing factor gene mutations, RNA expression changes in these genes can cause downstream splicing changes to promote tumor formation^22–24^. Thus, we performed differential gene expression (DE) analysis between high vs low SBI tumors in Cluster 6 for known splicing factors and related genes^25^ (Table S3C) and found 41.3% (N = 558/1350) to be significantly differentially expressed (adjusted p-value < 0.05, Figure 3B, Table S3F). Specifically, 71.4% (20/28) genes encoding the serine/arginine-rich splicing factor (SRSF) and heterogeneous nuclear ribonucleoproteins (hnRNP) families of trans-acting splicing factors known to directly influence exon-associated splicing^26^ were significantly DE between high vs low SBI tumors.

### Recurrent splicing aberrations alter the inclusion of known functional sites in transcripts

To further elucidate the aberrant splicing landscape of Cluster 6, we developed a robust and adaptable workflow to prioritize recurrent (N ≥ 2) differentially spliced (ΔPSI z-score > |2|) SE events with predicted functional impact (Figure 3C). We identified 23,674 recurrent differential SE splicing events in Cluster 6 tumors. Of these, we prioritized 8,369 with a putative functional effect, defined by the gain or loss of a known Uniprot functional site. These functional sites included changes to disulfide bonding (N = 630), localization signaling (N = 284), amino acid modification (N = 1,533), and domains (N = 6,946) (Table S4). To identify potentially targetable events, we selected functional splice events in kinases, resulting in 349 SE events. We reduced our focus to kinases with a known role in splicing regulation, narrowing our list of candidates to 14 SE events in 8 genes.

*CDC-like kinase 1 (CLK1)*, a known master modulator of alternative splicing^13^, was the most significantly correlated splicing factor related gene with Cluster 6 SE SBI (Table S5, Figure 3E). *CLK1* regulates the SR (Serine aRginine) family of splicing factor proteins through hyper-phosphorylation of the SR-rich peptide regions of SR proteins to induce cooperative RNA binding and increased activity^27–29^. Canonical *CLK1* requires exon 4 for activation^30^. We observed differential splicing of this exon, with seven tumors in Cluster 6 showing significant skipping (reduced inclusion) and an average ΔPSI of 0.437 (Table S4D). We used a sashimi plot to illustrate this, contrasting tumors with high versus low exon inclusion (Figure 3D). The majority of tumors showed high levels of *CLK1* exon 4 inclusion (Figure S3E) with a mean PSI of 0.790 and Cluster 6 showed the highest degree of variability of *CLK1* exon 4 PSI (Figure S3F).

Given the identification of *CLK1* exon 4 skipping as a recurrent event in Cluster 6 tumors, we examined whether *CLK1* expression was associated with overall splicing dysregulation. *CLK1* expression was significantly positively correlated with SE SBI in multiple clusters (Figure S4A), with the strongest correlation observed in Cluster 6 (Pearson’s R = 0.70, p = 2e-11, FDR = 2.4e-8; Figure 3E). Notably, among all splicing factors and RNA binding proteins (N = 1,350)^25^, *CLK1* expression showed the most significant positive correlation with SE SBI in Cluster 6. This relationship appeared to be driven by histology, with strong correlations observed in other HGGs (R = 0.67, p = 1.2e-13; Figure S4B) and DIPG/DMG tumors (R = 0.52, p = 0.0021; Figure S4B). Furthermore, expression of the canonical transcript ENST00000321356, which includes exon 4, was positively correlated with exon 4 PSI in Cluster 6 (R = 0.62, p = 3.8e-08; Figure 3E), as well as in all other clusters except Cluster 9 (Figure S4C). In Cluster 6, tumors with high *CLK1* exon 4 PSI had significantly worse OS (HR = 18.98, p = 0.03, Figure 3F) and EFS (HR = 78.63, p = 0.01, Figure 3G), even accounting for clinical covariates, suggesting a *CLK1*-associated splicing state rather than generalized spliceosome activation. Finally, we assessed *CLK1* exon 4 inclusion in non-tumor brain tissues using RNA-Seq data from the Evolutionary Developmental (Evo-Devo) Atlas (N = 59)^31^ and GTEx (N=2,642). *CLK1* exon 4 inclusion was significantly higher in Cluster 6 compared to GTEx but comparable to fetal Evo-Devo samples, and decreased with age across non-tumor tissues (*p* < 0.05), consistent with an oncofetal splicing pattern (Figure 3H). Together, these findings highlight *CLK1* exon 4 inclusion as a recurrent, functionally relevant splicing aberration in Cluster 6 tumors linked to widespread splicing dysregulation, adverse clinical outcomes, and an oncofetal splicing pattern, underscoring its potential as a biologically meaningful and therapeutically relevant target.

### *CLK1* is an oncogenic dependency in pediatric CNS tumors

**Figure 4.**
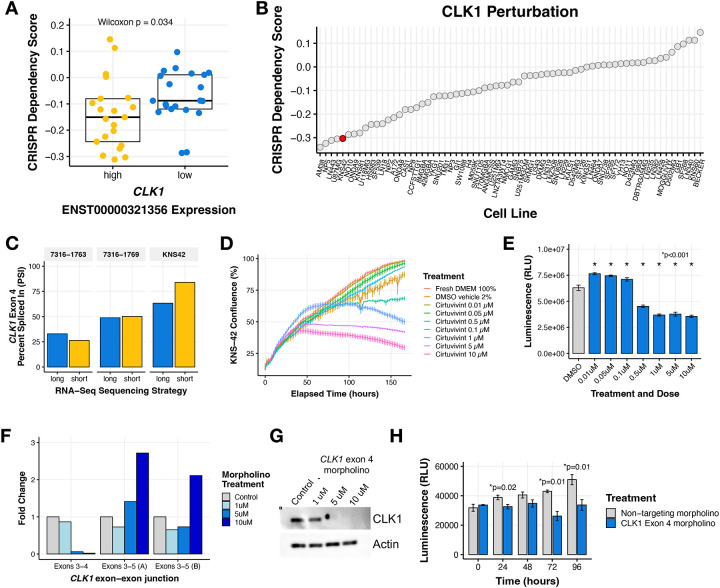
*CLK1* exon 4 is required for pediatric brain tumor cell line growth and viability. **(A)** Boxplot of DepMap dependency scores stratified by high or low *CLK1* exon 4 containing transcript expression in brain tumor cell lines. Wilcoxon p-value shown. **(B)** Ranked dotplot of DepMap dependency scores in brain tumor cell lines with pediatric line KNS-42 highlighted in red. **(C)** Proliferation of KNS-42 cells treated with increasing concentrations of pan-DYRK/CLK1 inhibitor Cirtuvivint over six days. **(D)** Day 3 cell viability of KNS-42 cells treated with increasing concentrations of Cirtuvivint. Stars denote Bonferroni-adjusted p-values following pairwise Student’s *t-tests*. **(E)** Barplot showing the RNA expression fold-change in cells treated with control morpholino or morpholino targeting the *CLK1* exon 3–4 junction or exon 3–5 junction. **(F)** Western blot of CLK1 with increasing morpholino treatment of 1, 5, and 10 μM. **(G)** Cell viability of cells treated with *CLK1* exon 4 morpholino or non-targeting morpholino. Stars denote within-time paired Student’s *t-tests*.

To experimentally test our findings from Cluster 6 tumors, we sought to evaluate *CLK1* and exon 4 inclusion as potential dependencies and therapeutic targets in pediatric brain tumor cell lines. We investigated the cancer Dependency Map (DepMap) portal and database and found that CNS and brain tumor cell lines with high expression of the exon 4 included transcript of *CLK1* (≥ third quantile mRNA expression of ENST00000321356) have significantly higher CRISPR dependency (lower scores) compared to *CLK1* low expressing cell lines (≤ first quantile) (Wilcoxon p = 0.034, Figure 4A). This observation was significant only to cell lines derived from CNS tumor and myeloid malignancies (Figure S5A), suggesting tissue- and tumor-specific regulation of *CLK1*. Of the DepMap profiled cell lines, the pediatric glioblastoma cell line KNS-42 had a strong dependency on *CLK1* (Figure 4B) and hence was chosen for further *in vitro* testing, along with two additional cell lines from our pediatric brain tumor cohort with high *CLK1* exon 4 PSI (7316–1763 and 7316–1769). We experimentally validated the exon 4 splice event identified from short-read RNA-Seq. Specifically, we performed long-read RNA-seq using Oxford Nanopore Technologies (ONT), which confirmed that these patient-derived cell lines contain a similar ratio of *CLK1* mRNA isoforms that either include or skip exon 4 (Figure 4C).

Next, we tested the impact of CLK1 inhibition in KNS-42 cells using the pan-Dyrk/Clk inhibitor cirtuvivint (SM08502)^32^. Using the IncuCyte Live Cell Analysis System to monitor real-time proliferation, we observed a significant reduction in cell growth at multiple concentrations over a 6-day period (Figure 4D). Additionally, we observed a dose-dependent decrease in cell viability using CellTiter-Glo at three days (Figure 4E) and six days (Figure S5B) post-treatment of 0.5, 1, 5, and 10 μM Cirtuvivint.

*CLK1* regulates the SR (Serine aRginine) family of splicing factor proteins through hyper-phosphorylation of the SR-rich peptide regions of SR proteins to induce cooperative RNA binding and increased activity^27–29^. We therefore postulated that the gain of *CLK1* exon 4 increases mRNA and subsequent protein production. To directly test this hypothesis, we modulated *CLK1* exon 4 splicing using targeted morpholino oligomers (see Online Methods), in which we forced exon 4 skipping in the KNS-42 cell line. We performed qRT-PCR and observed a near total loss of the *CLK1* exon 4 inclusion transcript at both 5 and 10 μM of exon 4 targeted morpholino, evidenced by reduced expression of the exon 3–4 junction. At these same concentrations, we observed increased *CLK1* exon 4 skipping using primers targeting the exon 3–5 junction (Figure 4F). Importantly, forced *CLK1* exon 4 skipping resulted in ablation of CLK1 protein at 5 and 10 μM (Figure 4G), corroborating previous work that *CLK1* exon 4 is required for full-length and catalytically active *CLK1*^33–35^. Next, we assessed the functional impact of *CLK1* exon 4 splicing using CellTiter-Glo and confirmed that cells with high *CLK1* exon 4 skipping (*CLK1* exon 4 targeting morpholino) exhibited significantly decreased viability compared to those with *CLK1* exon 4 inclusion (non-targeting morpholino) at 24, 72, and 96 hours (p ≤ 0.01, within-time Student’s *t-test*, Figure 4H). Taken together, we demonstrate that *CLK1* is a dependency in pediatric CNS tumors, required for cellular growth and viability in pediatric HGGs, and *CLK1* mRNA and protein is maintained through increased exon 4 inclusion.

**Figure 5.**
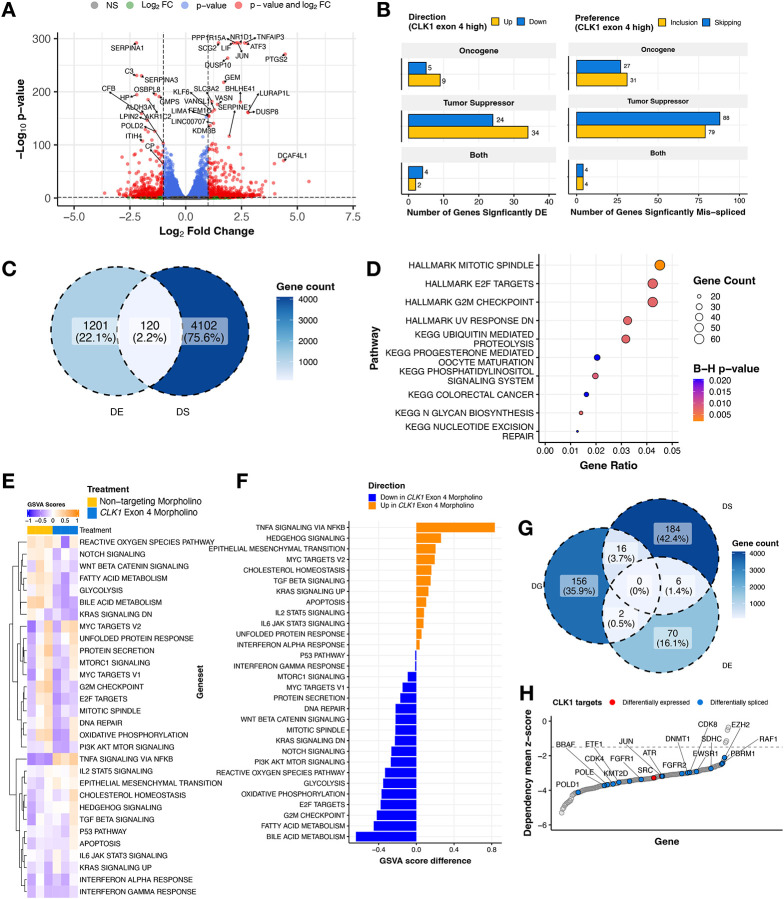
Perturbing *CLK1* disrupts RNA splicing and impairs oncogenic transcriptional programs. **(A)** Volcano plot illustrating genes differentially-expressed in KNS-42 cells treated with *CLK1* exon 4 targeting morpholino compared to cells treated with non-targeting morpholino. **(B)** Barplots displaying number of differentially expressed (DE) genes or differentially spliced (DS) genes affecting functional sites categorized by gene family. **(C)** Venn diagram depicting overlap of all DS and DE genes **(D)** Over-representation analysis of HALLMARK and KEGG pathways for DS cancer genes **(E)** Heatmap displaying single-sample HALLMARK GSVA scores for DS genes affecting functional sites in cells treated with *CLK1* exon 4 morpholino or non-targeting morpholino. **(F)** Barplots illustrating the mean GSVA score difference by treatment (n = 3 replicates per treatment). **(G)** Venn diagram depicting overlap of DS and DE genes and significant (Wald FDR < 0.05, z-score < −1.5) dependency genes (DG) identified in matched CBTN HGG cell lines through CRISPR dependency experiments from the Childhood Cancer Model Atlas (CCMA v3). **(H)** Ranked dotplot of significant CRISPR gene dependency mean zscores for pediatric HGG cell lines with *CLK1* expression and splicing-based target genes highlighted in red and blue, respectively.

To identify *CLK1* targets mediated by exon 4 splicing, we performed RNA-seq from KNS-42 cells treated with morpholino oligomers (N = 3 controls, N = 3 targeted to skip exon 4). We performed differential gene expression (DE) analysis and identified 1,322 genes with differential expression (569 upregulated, 753 downregulated) between cells treated with morpholino or non-targeting control (Figure 5A, Table S6A), including 79 oncogenes or tumor suppressor genes (TSGs, Figure 5B). Next, we identified a total of 8,741 unique differential splicing (DS) events within 4,222 genes (SE = 6,185; A5SS = 410, A3SS = 506, and RI = 526; (Table S6B–F), including 233 oncogenes or TSGs (Figure 5B). There were 120 genes (2.2%) which were both DE and DS (Figure 5C, Table S6G), indicating these may impact total protein abundance in tumors. DS genes were significantly over-represented for mitotic spindle, E2F targets, G2M checkpoint, and nucleotide excision repair pathways (Bonf-adj p < 0.05, Figure 5D). To further investigate the impact on DNA repair and other pathways, we performed GSVA of DNA repair and cancer signaling pathways on these DS oncogenes and TSGs and found that depletion of CLK1 leads to upregulation of TNFA, PI3K/AKT/MTOR, IL6/JAK/STAT3, and apoptosis pathway expression and downregulation of multiple DNA repair pathways (Figures 5E–F and S5C–D). Importantly, *CLK1* morpholino-mediated knockdown resulted in significant reduction of *CLK1* and additional known targets, *LRP5, AXIN2*, and *LEF1* (Log_2_FC < −0.1, B-H p-adj < 0.05, Table S6H) and downregulation of WNT signaling (Figure 5E–F), consistent with a known mechanism of Cirtuvivant^32^.

Finally, we asked whether *CLK1 exon 4* inclusion levels affect any of the essential oncogenes defined by the pediatric gene dependency maps of the Childhood Cancer Model Atlas^36^. We observed 18 of these genes also exhibit significant gene dependencies in established pediatric HGG cell lines (Figure 5F–G, Table S6I, Figure S6), including seven direct regulators of MAPK signaling: *BRAF, EZH2, RAF1, JUN, FGFR1, FGFR2*, and *SRC*. For instance, the expression levels of mRNAs encoding proto-oncogene *SRC*^37,38^ are higher in cells with high *CLK1* exon 4 (non-targeting morpholino), indicating that *CLK1* may enhance or promote *SRC* expression. The differential splicing cases are more complex as they affect multiple transcripts within a gene, but taken together, these data suggest that transcript-level changes mediated through *CLK1* differential exon 4 splicing may contribute to some of these dependencies, particularly DNA repair and MAPK signaling, consistent with the established link between aberrant splicing with cancer progression^39–41^.

## Discussion

Pediatric brain cancer remains the leading cause of disease-related mortality in children, and HGGs present challenges of chemotherapy resistance and surgical limitations. In this study, we conducted a large-scale analysis of differential alternative splicing across pediatric CNS tumors, revealing pervasive splicing dysregulation. We introduce the SBI as a quantitative, sample-level metric to measure differential splicing, enabling cross-histology comparisons without the requirement for matched normal controls. Notably, we did not observe our hypothesized inverse relationship between TMB and SBI; instead, we observed a weak but significant positive correlation between TMB and SBI. This suggests that splicing dysregulation in pediatric brain tumors may not simply serve as a compensatory mechanism in genetically “quiet” tumors, but could reflect broader disruptions in RNA processing programs.

Clustering tumors by highly variable skipped exon (SE) events identified 11 splicing-defined groups, many significantly enriched for specific histologies or molecular subtypes. Importantly, Cluster 6 emerged as the poorest prognosis group, enriched for HGGs and characterized by both high splicing variability and upregulation of the spliceosome pathway. This subgroup exhibited the greatest variance in SBI and survival analyses indicated that, paradoxically, higher spliceosome activity was associated with improved outcomes in these otherwise aggressive tumors. This suggests that the functional impact of splicing dysregulation may differ depending on the balance between oncogenic and potentially tumor-suppressive splicing programs.

By integrating RNA-seq–derived exon-level splicing quantification with CRISPR perturbation data and in contrast to GSVA scores, higher exon 4 PSI in *CDC-like kinase 1 (CLK1)*, a critical splicing factor and cell-cycle modulator, was linked to significantly worse prognosis, suggesting that global spliceosome activity and *CLK1*-driven exon inclusion may reflect distinct and potentially opposing aspects of splicing biology in Cluster 6 tumors. This divergence underscores that not all splicing pathway activation is equivalent. Broadly elevated spliceosome activity might reflect a compensatory or less oncogenic program, whereas *CLK1* exon 4 inclusion could mark a more malignant, dependency-associated splicing state.

Experimental modulation of *CLK1*, either through pharmacological inhibition or morpholino-directed exon 4 depletion in the KNS-42 cell line, resulted in significantly reduced cell proliferation and/or viability. Splicing modulation to deplete exon 4 ablated *CLK1* RNA and protein levels. Transcriptomic profiling following *CLK1* exon 4 depletion revealed widespread alterations in the splicing and expression of cancer-relevant genes, with enrichment for cell cycle, DNA repair, and MAPK signaling pathways, many of which are essential dependencies in pediatric HGG cell lines. These findings support the hypothesis that *CLK1*-mediated splicing sustains multiple oncogenic programs in CNS tumors. Notably, CLK family kinases, including *CLK1*, are already under clinical investigation in multiple adult malignancies through early phase trials. The Pan-Clk/Dyrk Inhibitor cirtuvivint (SM08502) is being used in a phase 1 clinical trial in patients with acute myeloid leukemia (AML) and myelodysplastic syndromes (MDS)^42^, and has shown preclinical efficacy in non-CNS solid tumors such as castrate-resistant prostate cancer, colorectal cancer, endometrial cancer, and non-small cell lung cancer^32,43–46^. An ATP-competitive, macrocyclic inhibitor of the CLK family, BH-30236^47^, is in a Phase 1/1b clinical trial for patients with AML and MDS^48^. Finally, the CLK1-specific inhibitor, CTX-712, is in Phase 1/2 trial for relapsed or refractory AML and high risk MDS^49^, underscoring *CLK1* and its family as a target across diverse tumor histologies. Our study extends the rationale for CLK1 inhibition to pediatric brain tumors, particularly those with high splicing burden and exon 4 inclusion.

In summary, our work maps the splicing landscape in pediatric CNS tumors, defines biologically and clinically relevant splicing subgroups, and establishes *CLK1* exon 4 inclusion as a recurrent, functional, and targetable aberration in aggressive pediatric brain tumors. Our approach to characterizing splicing aberrations and their functional consequences paves the way for future research into mRNA splicing-based mechanisms of tumorigenesis, the identification and development of therapies targeting aberrant splice events, and may even guide splicing-based diagnostics, all of which have the potential to improve the therapeutic landscape for pediatric brain cancers. Finally, we openly share the splicing data for all pediatric CNS tumors as a resource for the oncology community.

### Limitations of the study

In this study, initial splicing quantifications were performed using short-read RNA-Seq technology, which limits the interpretation of the full spectrum of splicing variation, particularly larger multi-exon transcripts or with genes that contain a high number of transcripts. While there are proteomics data for over 200 matched pediatric brain tumors publicly available, we were limited in sample size for DIPG or DMG and other HGGs, so it will be important to validate our findings in larger datasets as they become available. We did not have matched normal RNA for any tumor of origin tissue which precluded us from identifying mutually exclusive events that may also play a role in tumorigenesis. For example, within histologies (eg: LGG), the primary site of the tumor can vary widely depending on diagnosis and it would be ideal to match each tumor to its tissue of origin. Although this is not yet possible with available data, the creation of the upcoming developmental GTEx will be critical in the future. We mitigated these normal tissue limitations by utilizing multiple normal control cohorts as well as the SBI metric. Overall, we employed robust statistical techniques, cross-validated our findings with external datasets, and utilized orthogonal approaches and experimental methods where possible.

## Supplementary Material

Supplement 1

Supplement 2

Supplement 3

Supplement 4

Supplement 5

Supplement 6

Supplement 7

Supplement 8

## Data Availability

All pediatric brain tumor raw data are available upon request from the database of Genotypes and Phenotypes (dbGAP), accession number phs002517.v2.p2, and/or from the Children’s Brain Tumor Network (https://cbtn.org) and the Pacific Pediatric Neuro-Oncology Consortium (pnoc.us) for data not immediately available in dbGaP. All processed data used in this study were derived from the OpenPedCan project^15^ v13 data release at https://github.com/d3b-center/OpenPedCan-analysis. All code for the manuscript analyses and figures are openly available at https://github.com/rokitalab/clk1-splicing. RNA-Seq data from the GTEx project (dbGAP Accession phs000424) and the Evo-Devo atlas (Array Express Accession E-MTAB-6814) were harmonized with GENCODE v39 using the Kids First Data Resource Center workflow at https://github.com/kids-first/kf-rnaseq-workflow. For this study, we restricted to samples from individuals under 40 years of age. RNA-sequencing data from the *CLK1* morpholino experiment has been deposited in GSE273841. Merged primary matrices and summary files utilized in this manuscript were derived from are openly accessible via the download script in the https://github.com/rokitalab/clk1-splicing repository.

## Key Points

- Splicing profiles of 752 pediatric CNS tumors define 11 clusters linked to histology
- *CLK1* exon 4 inclusion is widespread in CNS tumors, producing active kinase isoform
- High *CLK1* exon 4 inclusion predicts poor prognosis in aggressive CNS tumors

## Importance of study

This study systematically characterizes alternative splicing across 752 primary pediatric central nervous system (CNS) tumors and reveals extensive heterogeneity in splicing programs. We identify 11 distinct splicing-defined clusters, with *Cluster 6* enriched for aberrant splice events, transcriptional dysregulation, and poor clinical outcomes. Within this high-burden cluster, *CDC-like kinase 1 (CLK1)* exon 4 was differentially spliced with higher inclusion significantly associated with worse event-free survival. Functional experiments show that pharmacological inhibition or splice-modulating depletion of *CLK1* significantly reduces tumor cell viability and proliferation, while altering the expression and splicing of numerous cancer-associated genes. These findings underscore the fundamental role of RNA splicing in the biology of pediatric brain tumors, the relevance of splice-defined tumor subgroups, and highlight splicing modulation as a promising therapeutic strategy in pediatric CNS tumors.
