# Supplementary material for: Characterization of aberrant splicing in pediatric central nervous system tumors reveals CLK1 as a candidate oncogenic dependency
Source: bioRxiv. 2025 Aug 19:2024.08.03.606419. Originally published 2024 Aug 6. Preprint. [Version 5] doi: 10.1101/2024.08.03.606419 (PMC11326178; doi:10.1101/2024.08.03.606419)
Supplement: Supplement 1 [file media-1.docx]

**Supplemental Figure Legends**

**Figure S1.** Distribution plots of SE splicing burden index (SBI) by histology for RI **(A)**, A5SS **(B)**, and A3SS **(C)** events. Shaded regions represent high (blue; ≥ Quartile 3) and low (yellow; ≤ Quartile 1) SBI groups.

Correlation plots for SBI vs TMB across the entire cohort including **(D)** and excluding **(E)** hypermutant and ultra-hyper mutant tumors. Pearson’s R and p-values are shown.

**Figure S2. (A)** Enrichment heatmaps for all histologies, **(B)** MB subtypes, **(C)** ATRT subtypes, and **(D)** HGG subtypes by cluster. Boxes contain N tumors, Odds ratio (OR) in parentheses, and stars denote Fisher’s Exact test FDR < 0.05. **(E)** Stacked barplot showing tumor histology membership in each cluster stratified by molecular subtype for EPN, HGG, LGG, and MB histologies. **(F)** Heatmap of RNA expression corresponding to the top 30 most variably expressed proteins within the KEGG spliceosome pathway (GSVA scores denote KEGG spliceosome scores) in **Figure 2E. (G)** Scatter plot of the mean KEGG protein z-scores versus KEGG spliceosome GSVA scores. Pearson’s R and p-value are shown. **(H)** Forest plot of cox proportional hazards multivariate EFS KEGG spliceosome GSVA score by SE SBI interaction model with covariates tumor resection, LGG group, cluster, and age at diagnosis. Black and white diamonds indicate statistically significant and not significant HRs, respectively, with intervals denoting 95% confidence intervals. Gray diamonds indicate reference levels of factor covariates.

**Figure S3. (A)** Boxplots of splicing burden index by cluster, colored by histology. All boxplots represent the 25th and 75th percentiles, and the bar represents the median. **(B)** Forest plot of cox proportional hazards multivariate EFS model of cluster 6 patients with covariates tumor resection, histology group, KEGG spliceosome GSVA score quartile, and age at diagnosis. Black and white diamonds indicate statistically significant and not significant HRs, respectively, with intervals denoting 95% confidence intervals. Gray diamonds indicate reference levels of factor covariates. **(C)** Kaplan-Meier plot for OS of cluster 6 patients, stratified by high or low SBI (4th and 1st quartiles, respectively). **(D)**  Oncoprint displaying mutation frequencies of key brain cancer genes in Cluster 6 samples with annotations for gender, cancer predisposition, histology, CNS region, and tumor mutation status. Boxplots of *CLK1* exon 4 PSI values by histology **(E)** and cluster **(F)**.

**Figure S4. (A)** Scatter plots showing log2-single exon SBI versus log2-*CLK1* TPM by cluster and **(B)** histology. **(C)** Scatter plots of *CLK1* exon 4 PSI correlation with expression of the canonical exon 4-containing transcript, ENST00000321356, by cluster. Pearson’s R and p-values are denoted.

**Figure S5. (A)** Boxplot of dependency scores stratified by high vs low *CLK1* exon 4 containing transcript expression across all available DepMap brain tumor cell lines. Within histology Wilcoxon p-values are shown. All boxplots represent the 25th and 75th percentile and the bar represents the median. **(B)** Cell viability assay after six days of treatment of KNS-42 cells with increasing concentrations of pan-DYRK/CLK1 inhibitor Cirtuvivint. **(C)** Heatmap presenting single-sample DNA repair pathway GSVA scores for DS genes affecting functional sites in cells treated with *CLK1* exon 4 morpholino or non-targeting morpholino. **(D)** Barplots displaying mean DNA repair pathway GSVA scores (n = 3 replicates per treatment).

**Figure S6. (A)** Ranked dotplot of significant CCMA v3 CRISPR gene dependency z-scores in individual CBTN cell lines with *CLK1* expression (red) and splicing-based (blue) target genes highlighted for HGG or **(B)** DMG patient-derived cell lines.

**Supplementary Table Legends**

**Table S1**: Sample metadata. **(A)** Readme and feature definitions. **(B)** Sample information with clinical metadata and demographics **(C)** CNS region definition from OpenPedCan[^1^](https://paperpile.com/c/ISfPKG/o4atf).

**Table S2**: Histology-specific splicing events. **(A)** Single exon differential splicing events. SpliceID includes gene name, mis-spliced exon start and end coordinates, upstream and downstream exon start and end coordinates. **(B)** Cluster membership for each stranded RNA library sample. **(C)** All nominally (p < 0.05) and significantly (p adj < 0.5) differentially-expressed pathways by cluster.

**Table S3**: **(A)** KEGG spliceosome gene list. **(B)** HUGO spliceosome component gene list **(C)** Splicing factor and related genes from [^2^](https://paperpile.com/c/ISfPKG/1D14). **(C)** Somatic mutations in HUGO genes across the cohort **(D)** Somatic mutations in HUGO spliceosome genes or **(E)** splicing factor and related genes across the cohort **(F)** DeSeq2 results comparing high vs low SBI cluster 6 tumors for splicing factors.

**Table S4**: Differential splicing events impacting functional sites in Cluster 6 samples. **(A)** Exon skipping DS events. **(B)** Exon inclusion DS events. **(C)** Functional splice variants subsetted for known kinases. **(D)** *CLK1* exon 4 PSI for each sample in the cohort.

**Table S5:** Pearson correlation coefficients between splicing factor gene (**Table S3C**) transcripts per million (TPM) and SE SBI by cluster.

**Table S6**: *CLK1* morpholino analyses. **(A)** Differential gene expression results from DeSeq2 and **(B)** rMATs results comparing treated with *CLK1* exon 4 morpholino and non-targeting morpholino. **(C)** Differential splicing events associated with SE that correspond to known Uniprot functional sites. **(D)** Differential splicing events associated with A5SS that correspond to known Uniprot functional sites. **(E)** Differential splicing events associated with A3SS that correspond to known Uniprot functional sites. **(F)** Differential splicing events associated with RI that correspond to known Uniprot functional sites. **(G)** Genes that are differentially expressed and spliced. **(H)** Differentially expressed *CLK1* target genes overlapping with known *CLK1* targets. **(I)** Differentially expressed or differentially spliced *CLK1* target genes overlapping essential oncogenes defined by CCMA v3. **(I)** **(J)** *CLK1* exon 3-4, exon 3-5 and exon 3-5 junction forward and reverse primer sets.

**Online Methods**

***CLK1* exon 4 related visualizations and correlations**

To visualize the *CLK1* exon 4 splice event, we utilized the R package *ggsashimi*[*^3^*](https://paperpile.com/c/ISfPKG/Bochu). We correlated *CLK1* exon 4 PSI values with *CLK1-201* or *CLK1* TPM. We computed Pearson correlation coefficients and p-values of this plot using the R package *ggpubr*[*^4^*](https://paperpile.com/c/ISfPKG/JJZsG)*.* High *CLK1* exon 4 inclusion tumors were defined as those with PSI values above the 75th percentile, while low SBI samples were those with PSI values below the 25th percentile comparing across all samples.

**Splicing burden index (SBI) calculation**

The following describes the SBI calculation used in the manuscript.

Let X be the list of all samples, where Xᵢⱼ represents the j-th item in the i-th sample.

Let n be the number of items in each sample.

Let SE be the splice event of interest.

Let SEᵢ be the number of splice events in the i-th sample.

Let mean_SE_ be the mean of the splice event across all samples.

Let σ_SE_ be the standard deviation of the splice event across all samples.

Let SBI be the proportion of splice events that have z-scores > |2| out of the total number of splice events in a particular sample.

Then the equation for SBI is:


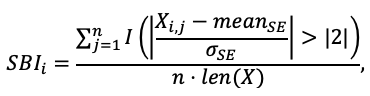


*where* i = 1 to len(X) and j = 1 to n

Let **X** be the set of all samples.

Let **Xᵢⱼ** represent the PSI value of the **j-th splice event (SE)** in the **i-th sample**.

Let **n** be the number of splice events measured in each sample.

Let **SEᵢ** be the number of observed splice events (non-missing) in sample **i**.

Let **meanSEⱼ** be the **mean PSI** of splice event **j** across all samples.

Let **σSEⱼ** be the **standard deviation** of splice event **j** across all samples.

For each PSI value **Xᵢⱼ**, compute the z-score:

**zᵢⱼ = (Xᵢⱼ − meanSEⱼ) / σSEⱼ**

**SBIᵢ = [Number of splice events with |zᵢⱼ| > 2] / SEᵢ**

Or, using summation notation: **SBIᵢ = (Σ₍ⱼ₌₁₎ⁿ I(|zᵢⱼ| > 2)) / SEᵢ**

Where:

- **I(condition)** = 1 if condition is true, 0 otherwise.
- **SEᵢ** = total number of valid (non-NA) splice events in sample *i*.

We compared PSI values of each primary tumor against all other tumors in the cohort. We first computed mean and standard deviation metrics for each alternative splicing event observed in at least one sample. Then for each sample in each group or histology, we identified the proportion of genes that underwent aberrant splicing as defined by a z-score > |2| across the entire transcriptome that undergoes alternative splicing.

**Consensus clustering**

We filtered the PSI matrix for splice events reported in > 25% of samples, and performed hierarchical clustering of samples based on the top 5,000 splice events with the highest PSI variance using the Euclidean distance measure and the Ward D2 agglomeration method. We determined the optimal number of clusters using the “elbow method”; briefly, we plotted the within-cluster sum of squares (WCSS) against the cluster number, and the x-axis value at which an inflection point was observed was defined as the optimal cluster number. We observed distinct clustering of samples by RNA library type within tumor histologies, and therefore re-ran clustering analyses separately for stranded and poly-A stranded libraries. We assessed enrichment of tumor histologies and molecular subtypes within identified clusters using Fisher’s exact tests, and defined significant enrichment of diagnoses within clusters at FDR < 0.05.

**Clustering-based differential expression or pathway enrichment**

We performed gene set variation analysis (GSVA) on expression data derived from stranded RNA-seq libraries to calculate GSVA scores for KEGG spliceosome and HALLMARK cancer pathways using the *GSVA* R package[^5^](https://paperpile.com/c/ISfPKG/jw8i4). We identified differentially expressed pathways between clusters using the *limma* R package[^6^](https://paperpile.com/c/ISfPKG/9i9gn), and visualized cluster differential expression using the *pheatmap* R package[^7^](https://paperpile.com/c/ISfPKG/NbwXv).

**Differential expression and visualization**

Differential expression was performed based on a model using the negative binomial distribution, a method employed by the R package *DeSeq2*[*^8^*](https://paperpile.com/c/ISfPKG/mjxPq). Those differential genes that had a p-value < 0.05 were deemed as significantly up or down-regulated. Volcano plots were generated by the *EnhancedVolcano* R package. Bar plots were generated using the R package *ggplot2*[*^9^*](https://paperpile.com/c/ISfPKG/gF816). Note: differential expression analyses were limited to stranded-only RNA-seq samples in order to limit batch effects.

**Identification of recurrent functional differential splicing variants in pediatric HGGs**

To identify differential or aberrant alternative splicing events, we assessed the percent spliced in (PSI) value of each splice event relative to the median PSI value of splice event across all samples. Splicing events with a ΔPSI exceeding |2| z-scores from the median PSI value were classified as differential or aberrant. For these events, we computed average ΔPSIs and generated bed files for each mis-spliced exon event. We then obtained bed files of known functional annotations as defined by Uniprot release 2025_03[^10^](https://paperpile.com/c/ISfPKG/3WoDc). We ran bedtools v2.30[^11^](https://paperpile.com/c/ISfPKG/3yUqs) to find the overlap between mis-spliced exons and functional features using the command `bedtools intersect -wo -a`. We then plotted summary data by functional category (disulfide bonding sites, localization signals, amino acid modifications, and other).

**Upset R and Volcano plots**

To visualize the intersections of multiple sets, we employed the UpSetR[^12^](https://paperpile.com/c/ISfPKG/eL8XE) plot in R. The input data consisted of differential and recurrent splicing events, if it was > 2 z-scores from the meanPSI and 2% of the histology-specific cohort. Volcano plots were generated by the *EnhancedVolcano* R package.

**Splicing burden index and tumor mutation burden correlations**

We identified samples with available data for both SBI (RNA-Seq) and WGS or WXS tumor mutation burden (TMB) from OpenPedCan[^1^](https://paperpile.com/c/ISfPKG/o4atf). Using the R package *ggscatter*, we performed a Pearson correlation analysis to examine the relationship between SBI and TMB. To ensure robustness, we repeated this analysis after excluding hyper-mutated samples (defined as those with TMB ≥ 10). Subsequently, we compared the distribution of TMB between high SBI and low SBI tumor samples using the Wilcoxon rank-sum test. High SBI samples were defined as those with SBI values above the 75th percentile, while low SBI samples were those with SBI values below the 25th percentile. The analyses were conducted across all samples and further stratified according to `plot_group`, as specified in the histologies clinical file.

**Pathway over-representation analysis (ORA) and gene set variation analysis (GSVA)**

We conducted over-representation analysis (ORA) using the R package clusterProfiler[^13^](https://paperpile.com/c/ISfPKG/dQjOh) and pathway data from the msigdbr package[^14^](https://paperpile.com/c/ISfPKG/yAUAj), including “CP:KEGG", "CP:BIOCARTA", “CP:HALLMARK”, and “TFT:GTRD.” After inputting the genes of interest (e.g. differentially spliced), we applied a p-value cutoff of 0.05 and used the Benjamini-Hochberg (BH) method for p-value adjustment. For visualization of the over-represented pathways, we employed the `enrichplot::dotplot()` function, displaying the gene ratio and the count of genes in each pathway.

To perform Gene set variation analysis (GSVA) we utilized the R packages `GSVA` and `msigdbr`. Expression data for our samples, sourced from OpenPedCan v13[^1^](https://paperpile.com/c/ISfPKG/o4atf), were used to compute gene-set enrichment scores. Genes with zero variance were excluded from the analysis. We then assessed enrichment in Hallmark, KEGG, and custom pathways from Knijnenburg et al[^15^](https://paperpile.com/c/ISfPKG/hbZyE). Gaussian-distributed scores were calculated using *gsvaParam* function in R. The results were visualized using heatmaps of GSVA scores, generated with the R packages *ComplexHeatmap* and *circlize*.

**Oxford Nanopore Technologies (ONT) Targeted Long-Read RNA-Sequencing**

We designed primers to bind to exons present in all isoforms of *CLK1* to ensure full coverage of all alternative splicing events. 5 ng of cDNA were amplified with LongAmp Taq 2X Master Mix (M0287S, New England Biolabs) for 25 cycles. The resulting amplicons were subjected to amplicon-seq (SQKNBD112.24, ONT) library preparation, loaded into a Spot-ON flow cell R9 Version (FLO-MIN112, ONT), and sequenced in a MinION Mk1C device (ONT) until at least 1,000 reads per sample were obtained. Results were aligned using Minimap2 version 2.24-r1122 and visualized in IGV version 2.12.3.

**DepMap and CRISPR dependency analyses**

Datasets comprising gene transcript expression, cell line information, and CRISPR dependency scores were downloaded from DepMap (version 24Q2). The expression of *CLK1* ENST00000321356 (exon 4 containing transcript) was categorized into high and low TPM expression, defined by values above the 75th quantile and below the 25th quantile, respectively. CRISPR dependency scores were plotted on the y-axis, and Wilcoxon tests were conducted to compare high versus low TPM expression groups. These were stratified for each cell line type. Additionally, CRISPR dependency scores for all CNS/brain cell lines were plotted, with KNS-42 highlighted in red. For the Childhood Cancer Model Atlas CRISPR dependency analyses, we acquired data from the Childhood Cancer Model Atlas[^16^](https://paperpile.com/c/ISfPKG/Q57Pz). We plotted CRISPR dependency scores (z) on the y-axis for each gene in CBTN pediatric HGG cell lines, either as median scores or stratified by individual patients with genes of interest highlighted.

**Proteogenomic analysis**

Pediatric proteomics, phosphoproteomics, and RNA data were obtained from the Clinical Proteomic Tumor Analysis Consortium (CPTAC) via the ProTrack: Pediatric Brain Tumor open-source web portal. Data and z-scores were computed using the methods described by Petralia et al[^17^](https://paperpile.com/c/ISfPKG/4lkM5).

**Bibliography**

1. [Geng Z, Wafula E, Corbett RJ, et al. The Open Pediatric Cancer Project. *bioRxiv*. Published online July 11, 2024. doi:](http://paperpile.com/b/ISfPKG/o4atf)[10.1101/2024.07.09.599086](http://dx.doi.org/10.1101/2024.07.09.599086)

2. [Sebestyén E, Singh B, Miñana B, et al. Large-scale analysis of genome and transcriptome alterations in multiple tumors unveils novel cancer-relevant splicing networks. *Genome Res*. 2016;26(6):732-744.](http://paperpile.com/b/ISfPKG/1D14)

3. [Garrido-Martín D, Palumbo E, Guigó R, Breschi A. ggsashimi: Sashimi plot revised for browser- and annotation-independent splicing visualization. *PLoS Comput Biol*. 2018;14(8):e1006360.](http://paperpile.com/b/ISfPKG/Bochu)

4. [ggpubr: “ggplot2” Based Publication Ready Plots. February 10, 2023. Accessed July 10, 2024.](http://paperpile.com/b/ISfPKG/JJZsG) <https://rdrr.io/cran/ggpubr/>

5. [Hänzelmann S, Castelo R, Guinney J. GSVA: gene set variation analysis for microarray and RNA-seq data. *BMC Bioinformatics*. 2013;14:7.](http://paperpile.com/b/ISfPKG/jw8i4)

6. [Ritchie ME, Phipson B, Wu D, et al. limma powers differential expression analyses for RNA-sequencing and microarray studies. *Nucleic Acids Res*. 2015;43(7):e47.](http://paperpile.com/b/ISfPKG/9i9gn)

7. [Kolde R, Kolde MR. Package “pheatmap.” *R package*. 2015;1(7):790.](http://paperpile.com/b/ISfPKG/NbwXv)

8. [Love M, Anders S, Huber W. Differential analysis of count data--the DESeq2 package. *Genome Biol*. 2014;15(550):10-1186.](http://paperpile.com/b/ISfPKG/mjxPq)

9. [Wickham H. Ggplot2. *Wiley Interdiscip Rev Comput Stat*. 2011;3(2):180-185.](http://paperpile.com/b/ISfPKG/gF816)

10. [Wu CH, Apweiler R, Bairoch A, et al. The Universal Protein Resource (UniProt): an expanding universe of protein information. *Nucleic Acids Res*. 2006;34(Database issue):D187-D191.](http://paperpile.com/b/ISfPKG/3WoDc)

11. [Quinlan AR, Hall IM. BEDTools: a flexible suite of utilities for comparing genomic features. *Bioinformatics*. 2010;26(6):841-842.](http://paperpile.com/b/ISfPKG/3yUqs)

12. [Conway JR, Lex A, Gehlenborg N. UpSetR: an R package for the visualization of intersecting sets and their properties. *Bioinformatics*. 2017;33(18):2938-2940.](http://paperpile.com/b/ISfPKG/eL8XE)

13. [Wu T, Hu E, Xu S, et al. clusterProfiler 4.0: A universal enrichment tool for interpreting omics data. *Innovation (Camb)*. 2021;2(3):100141.](http://paperpile.com/b/ISfPKG/dQjOh)

14. [Dolgalev I. msigdbr: MSigDB gene sets for multiple organisms in a tidy data format. *R package version*. 2020;7(1).](http://paperpile.com/b/ISfPKG/yAUAj)

15. [Knijnenburg TA, Wang L, Zimmermann MT, et al. Genomic and Molecular Landscape of DNA Damage Repair Deficiency across The Cancer Genome Atlas. *Cell Rep*. 2018;23(1):239-254.e6.](http://paperpile.com/b/ISfPKG/hbZyE)

16. [Sun CX, Daniel P, Bradshaw G, et al. Generation and multi-dimensional profiling of a childhood cancer cell line atlas defines new therapeutic opportunities. *Cancer Cell*. 2023;41(4):660-677.e7.](http://paperpile.com/b/ISfPKG/Q57Pz)

17. [Petralia F, Tignor N, Reva B, et al. Integrated Proteogenomic Characterization across Major Histological Types of Pediatric Brain Cancer. *Cell*. 2020;183(7):1962-1985.e31.](http://paperpile.com/b/ISfPKG/4lkM5)
