# Supplementary figures and images for: Characterization of aberrant splicing in pediatric central nervous system tumors reveals CLK1 as a candidate oncogenic dependency
Source: bioRxiv. 2025 Aug 19:2024.08.03.606419. Originally published 2024 Aug 6. Preprint. [Version 5] doi: 10.1101/2024.08.03.606419 (PMC11326178; doi:10.1101/2024.08.03.606419)

Figure S1

A

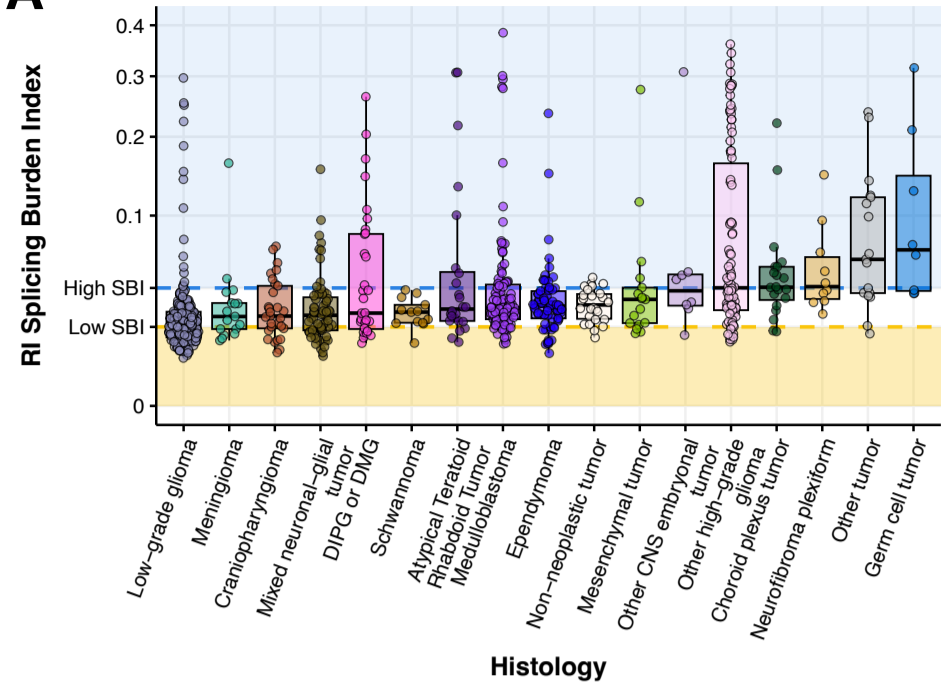

B

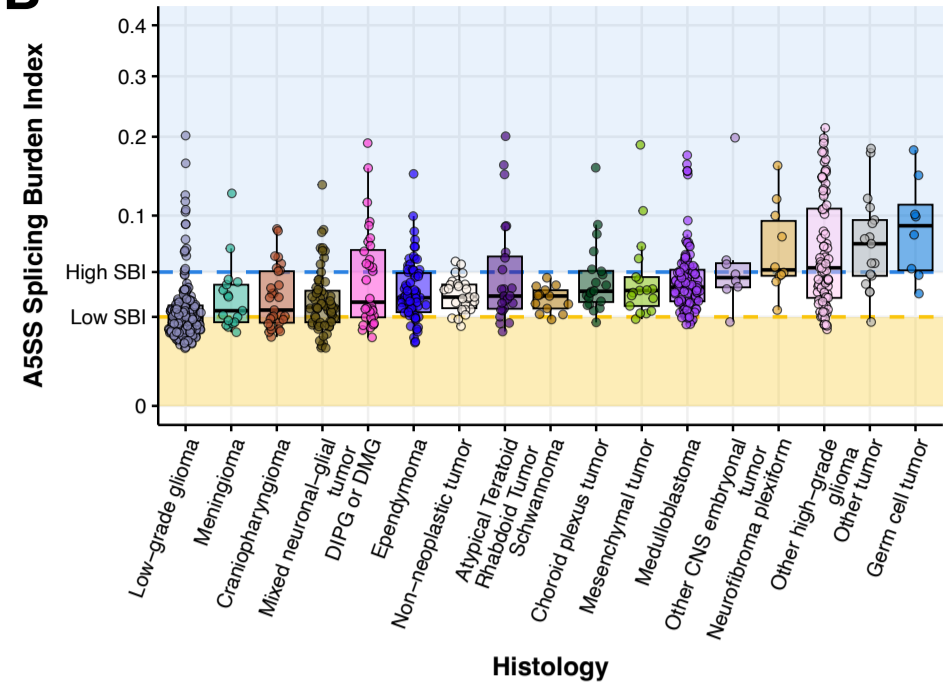

C

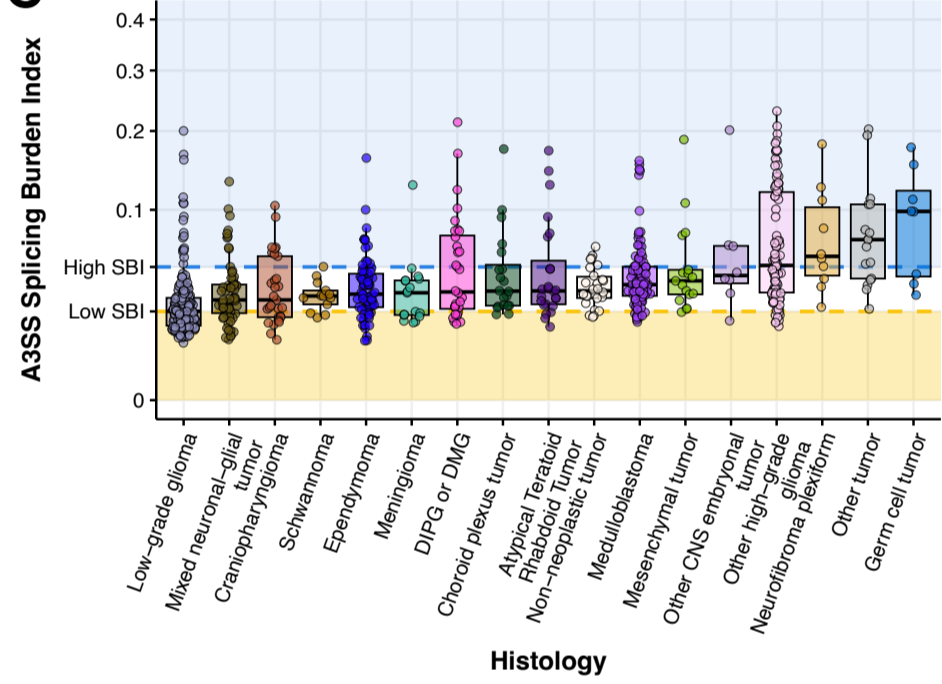

D

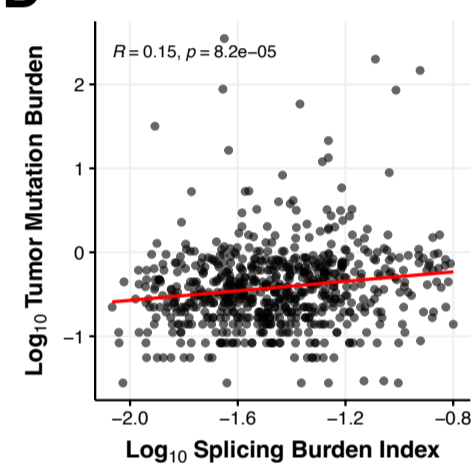

E

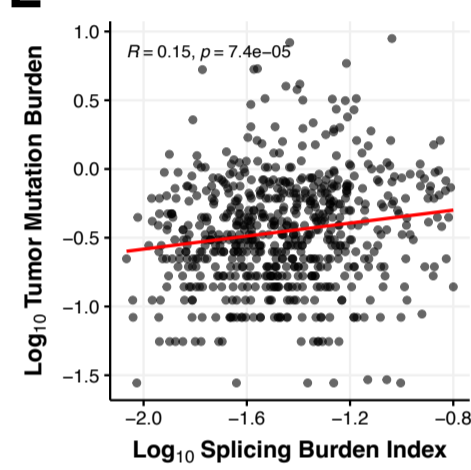

Figure S2

A

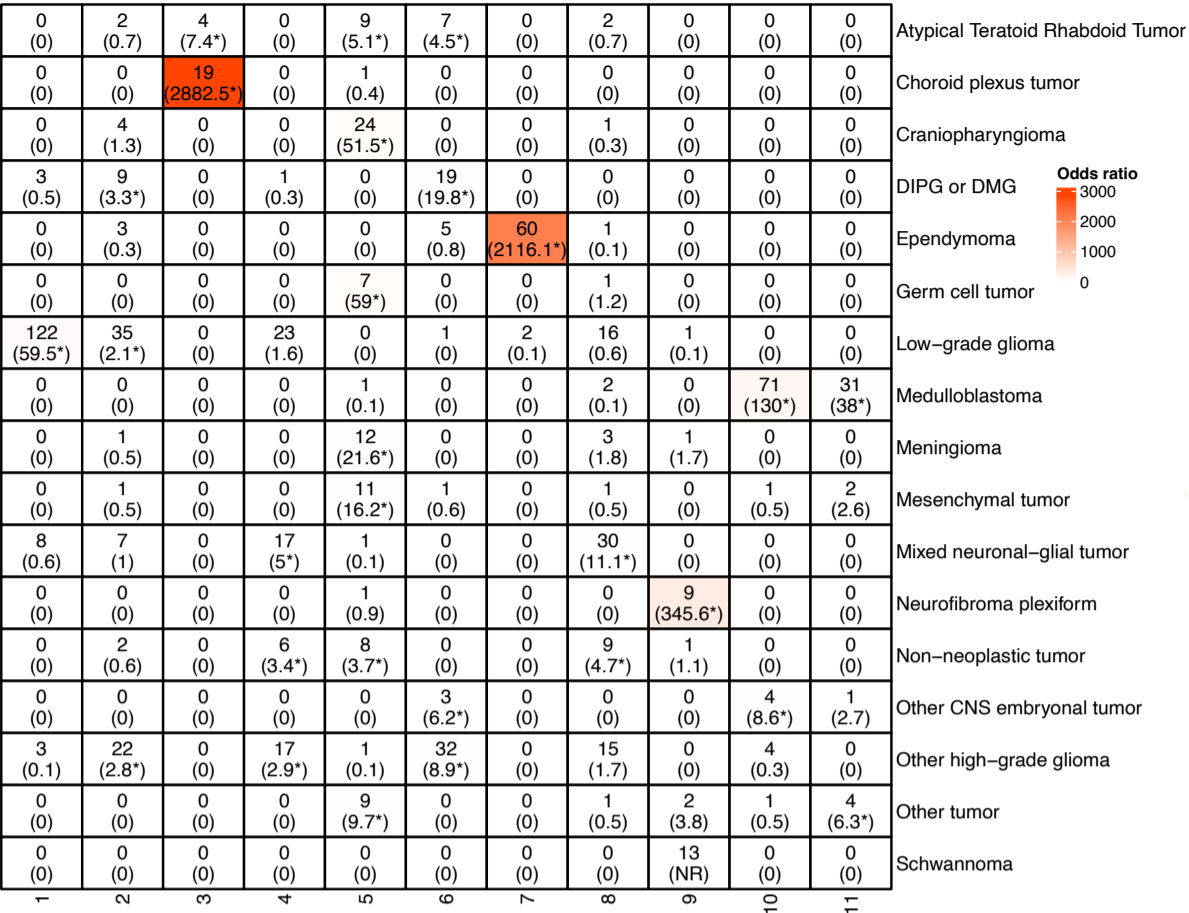

B

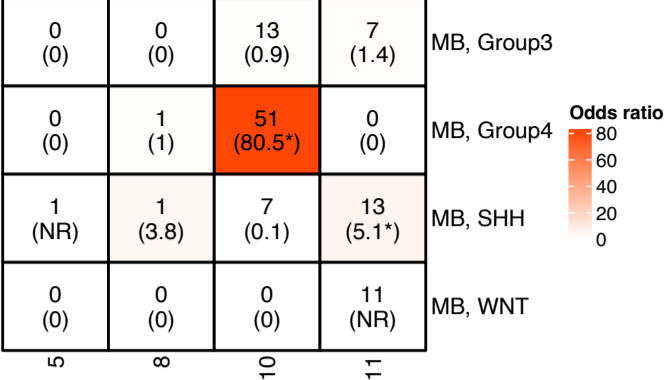

C

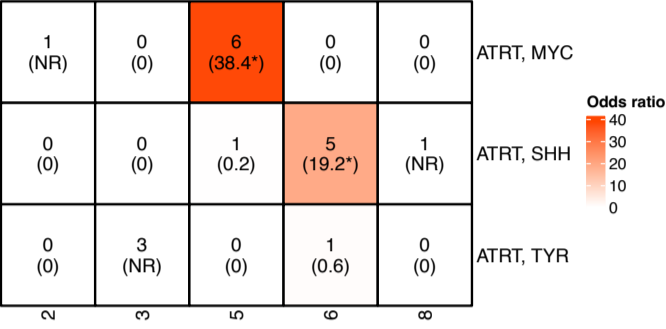

D

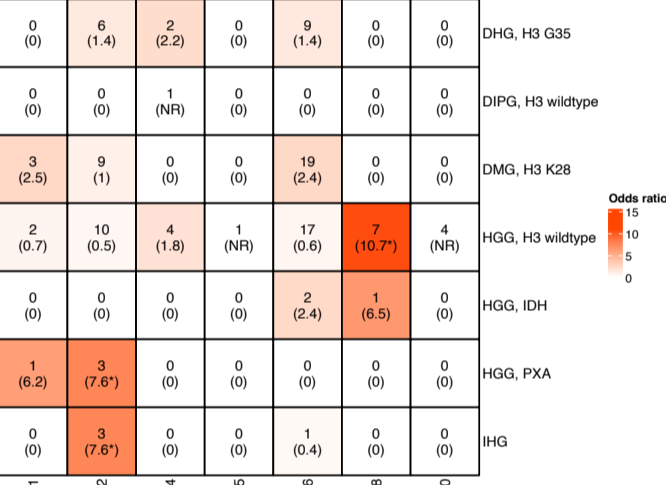

E

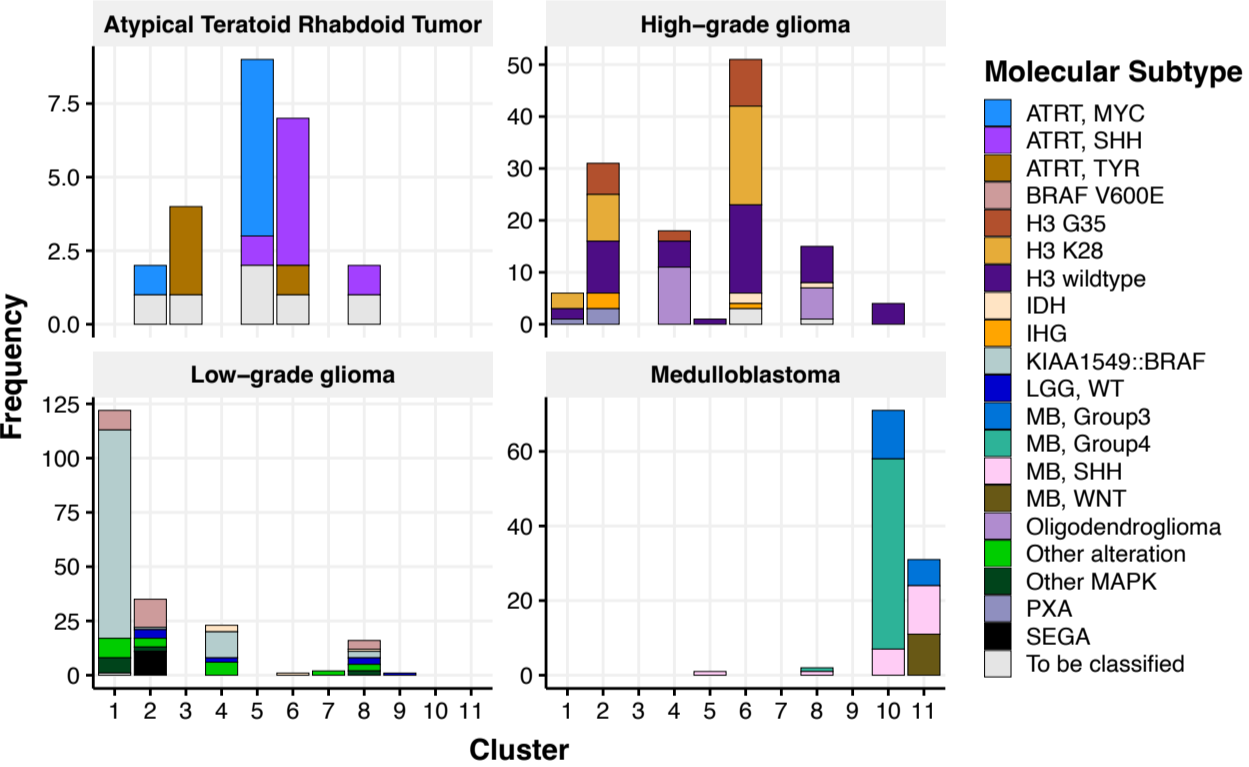

G

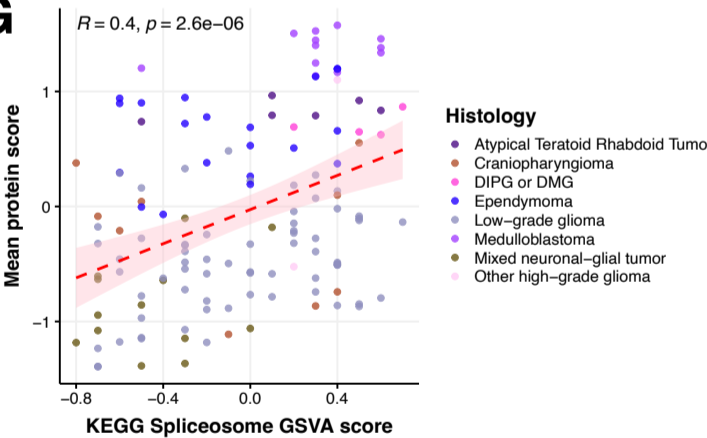

F

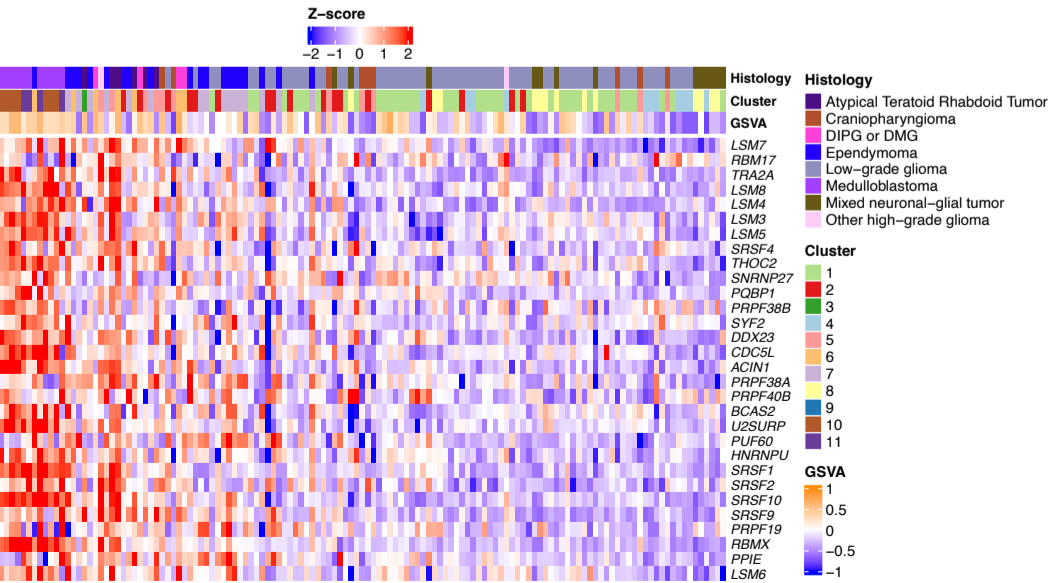

H

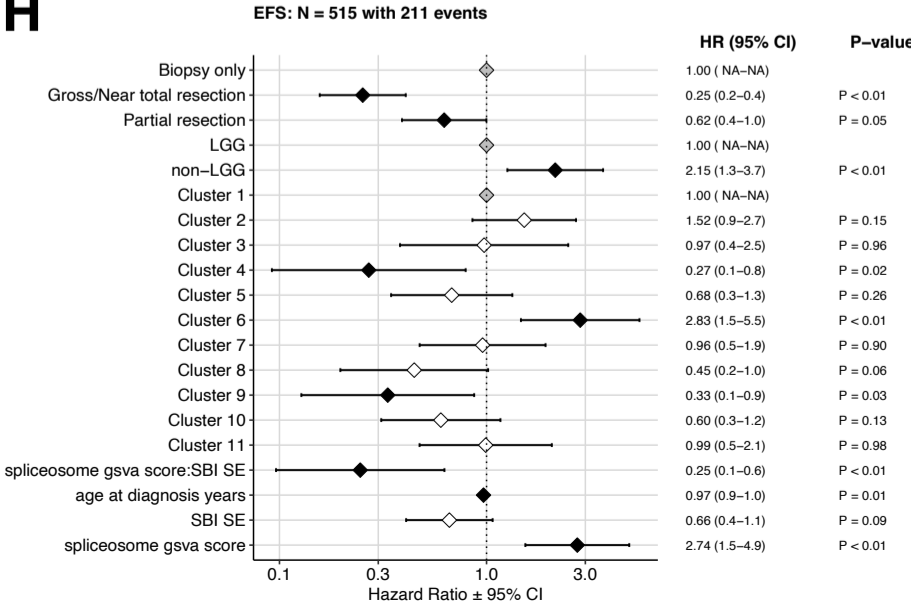

Figure S3

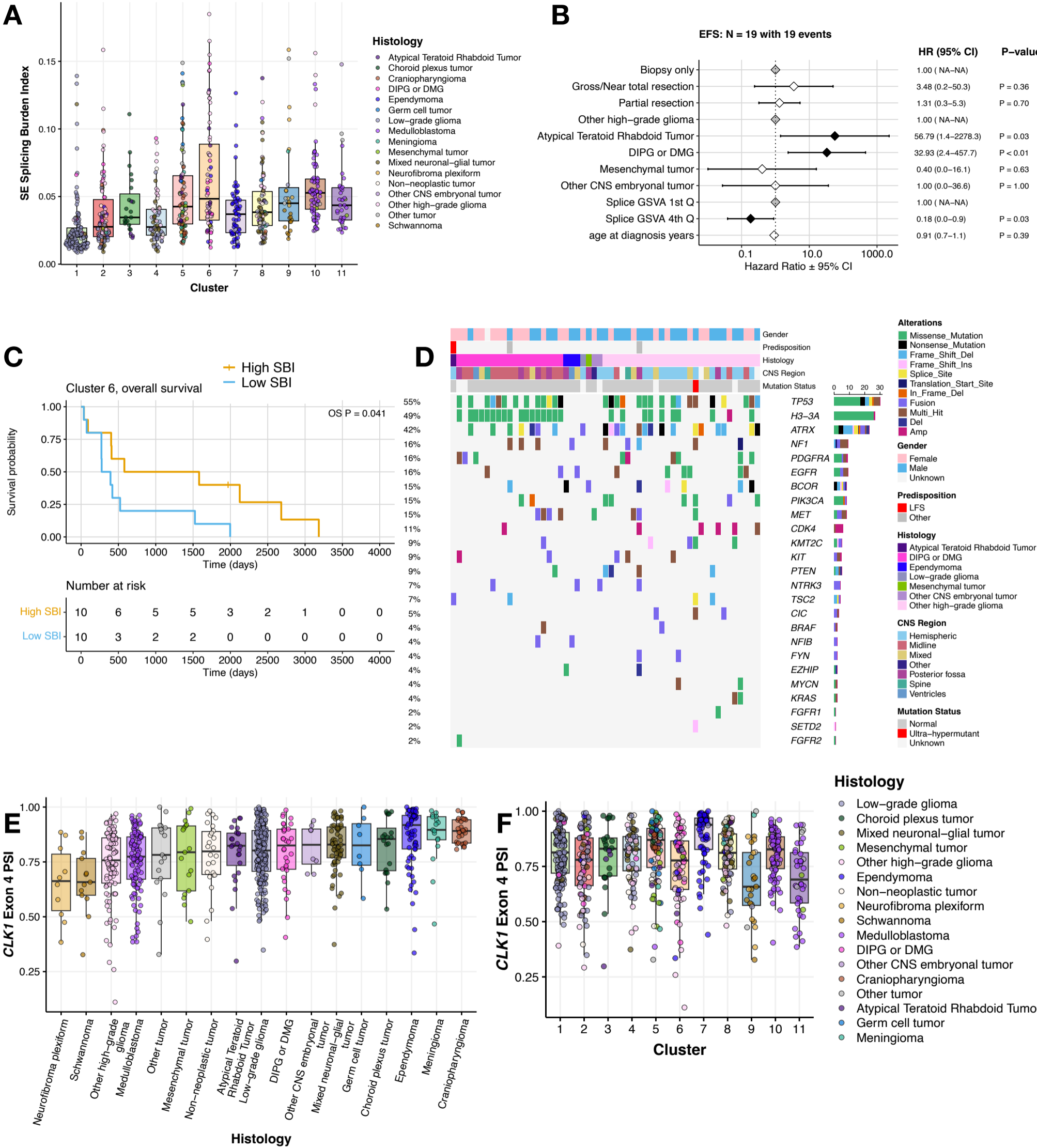

Figure S4

A

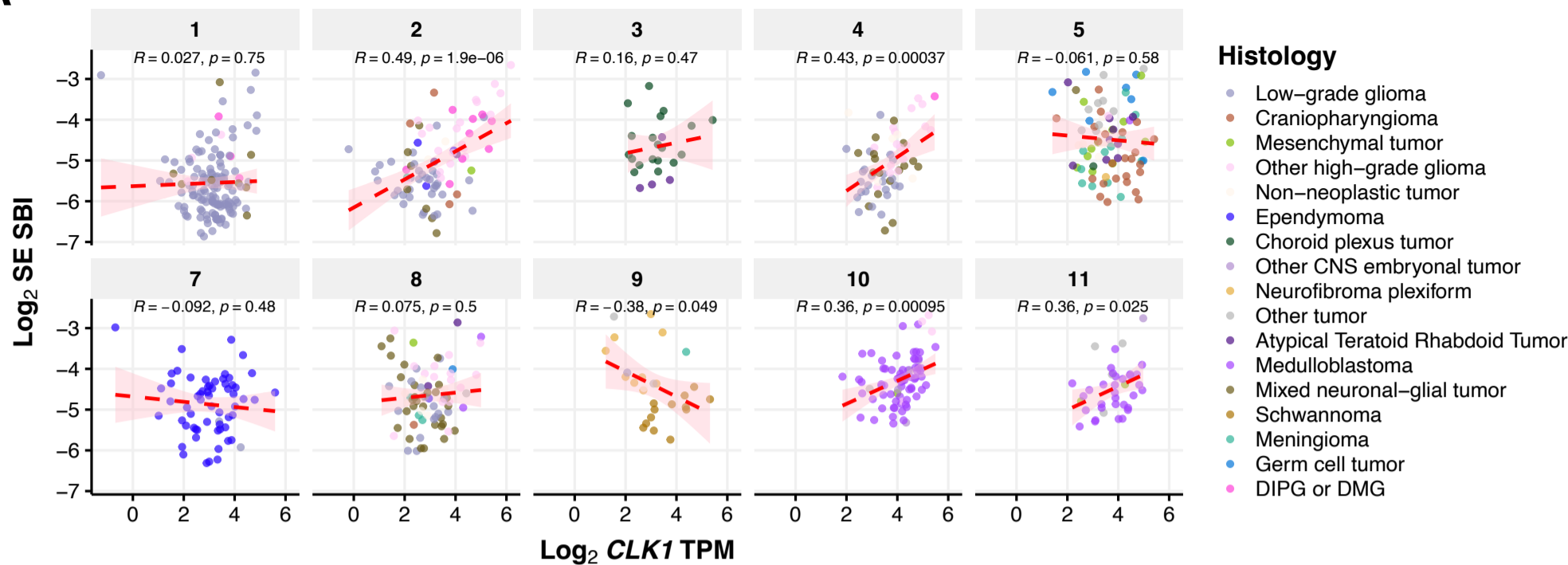

B

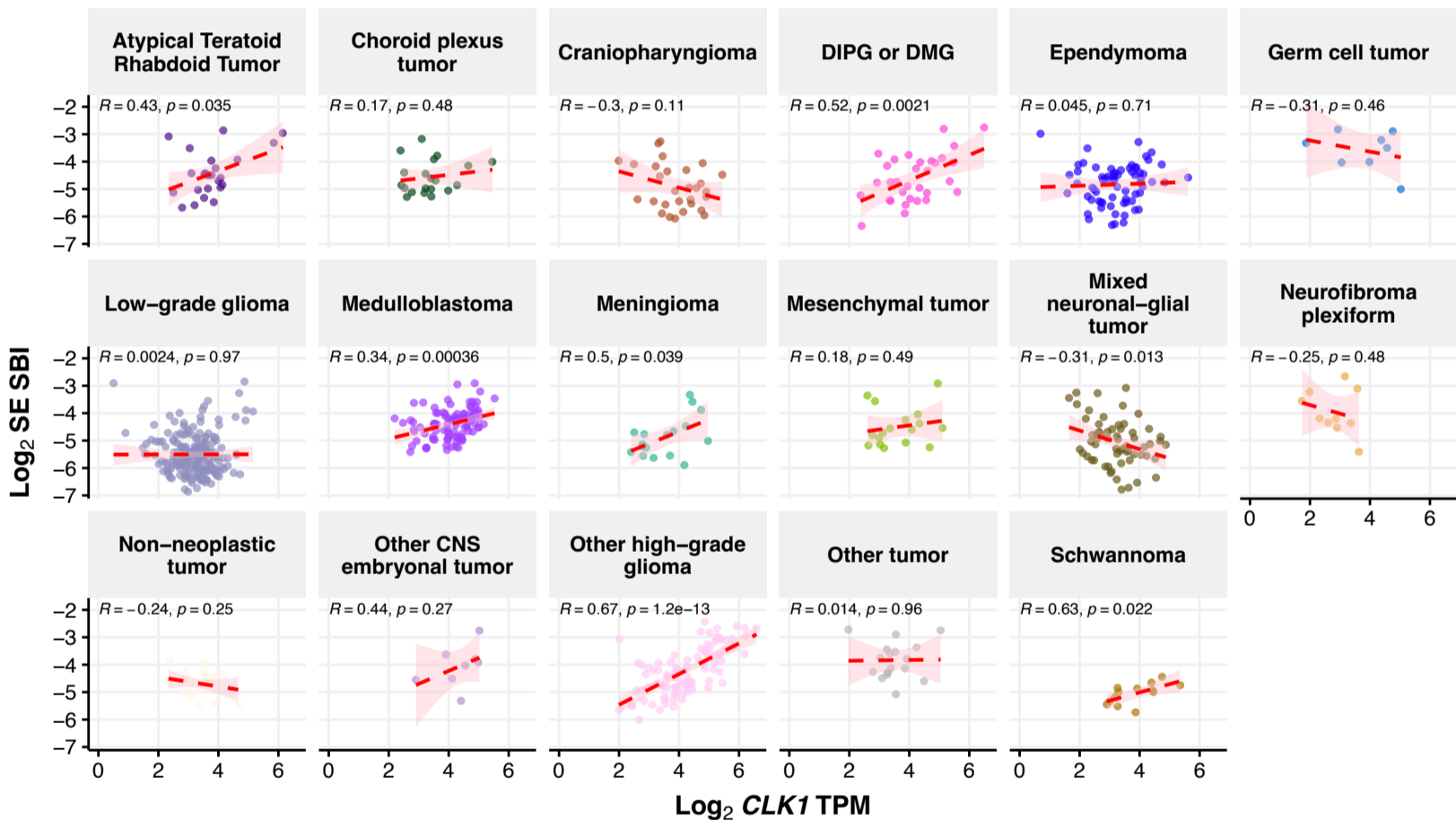

C

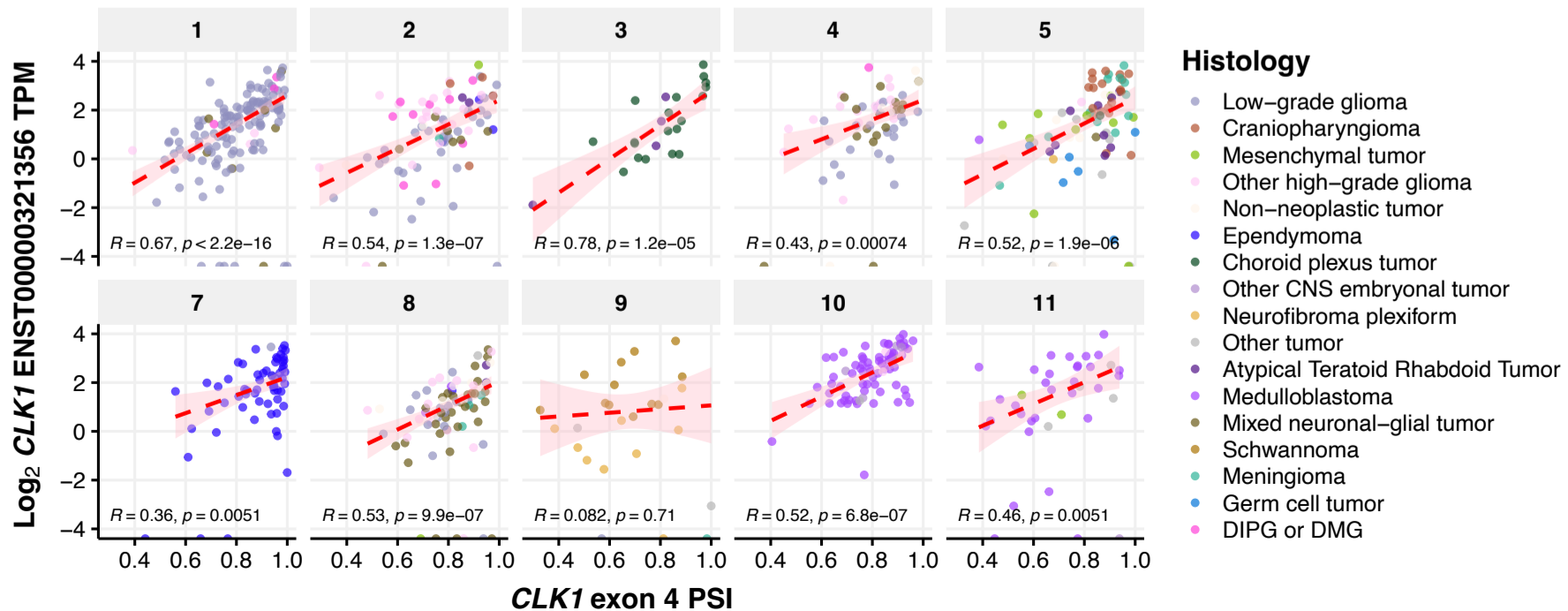

Figure S5

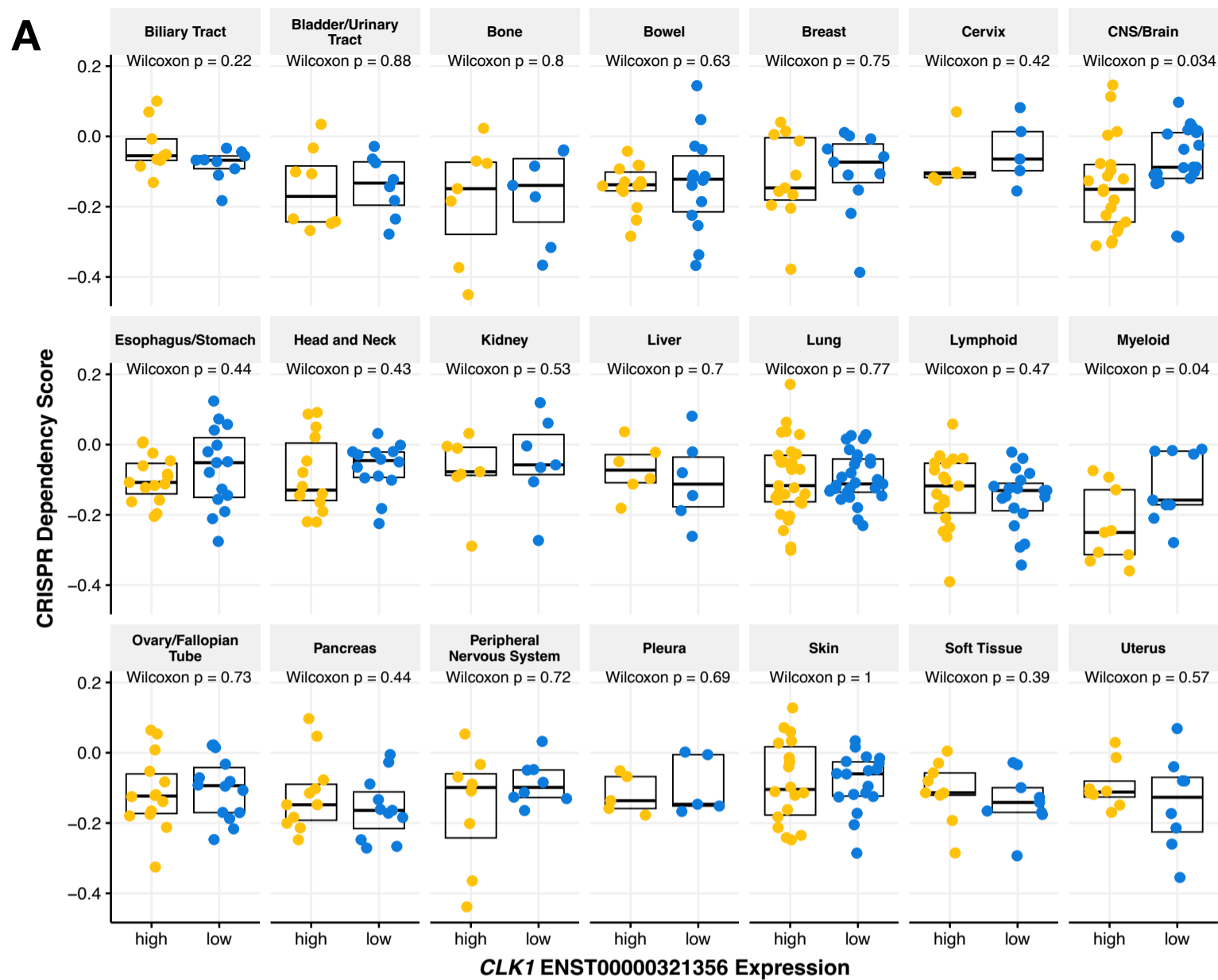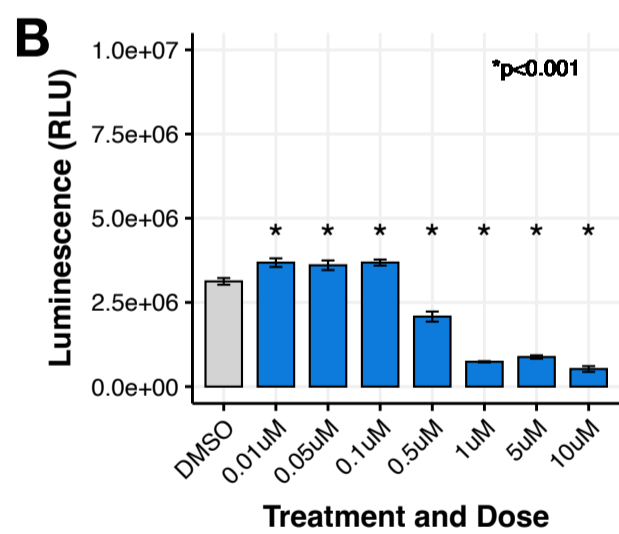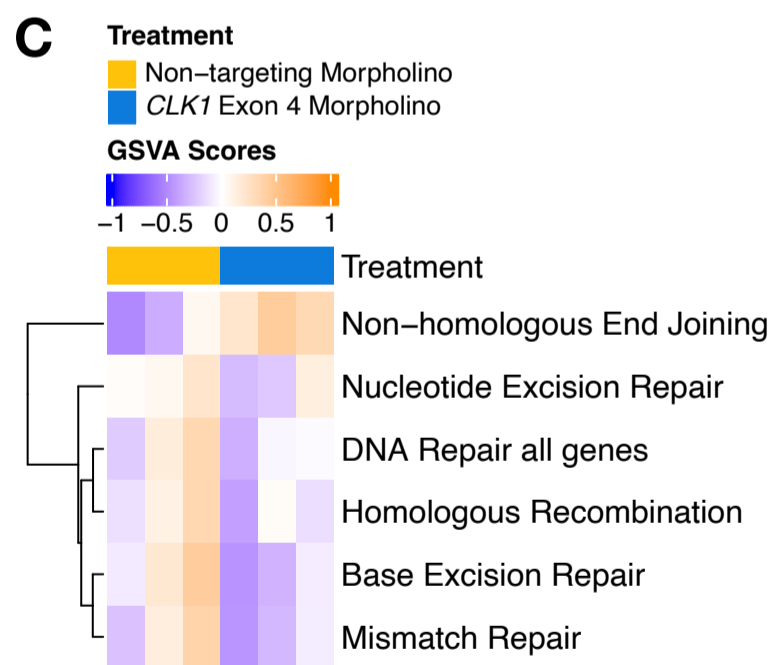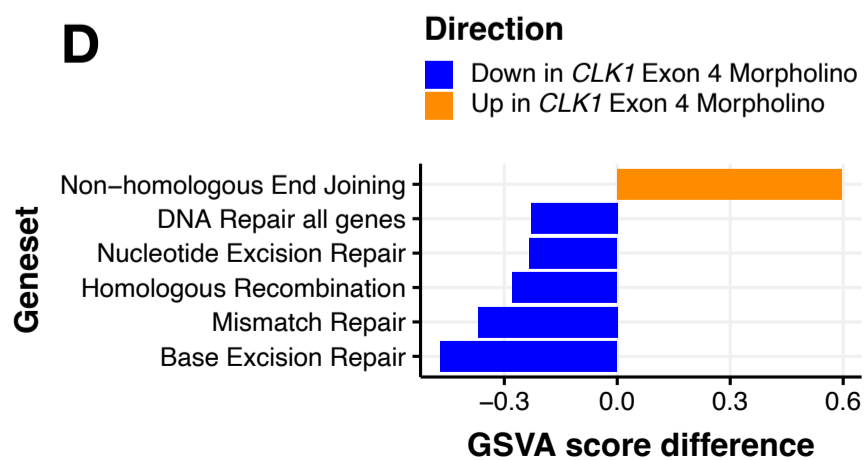

Figure S6

A

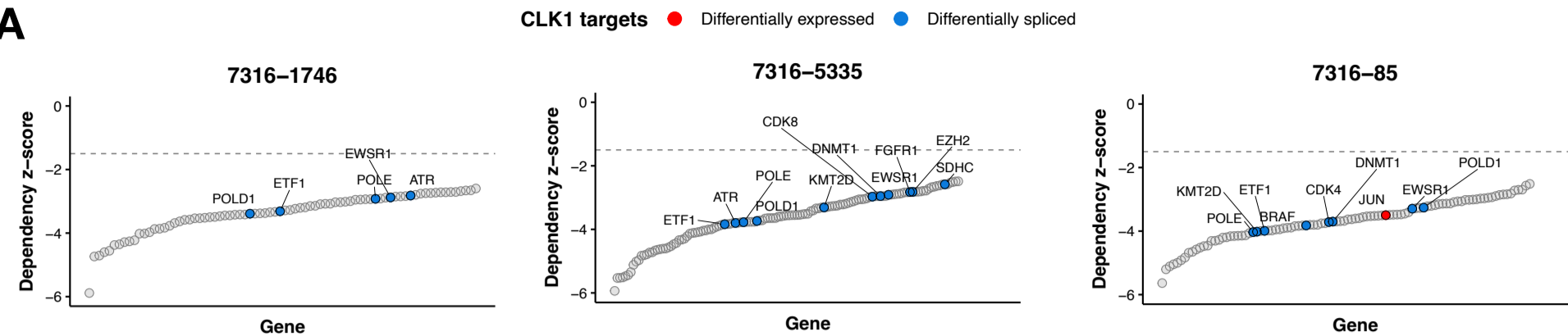

B

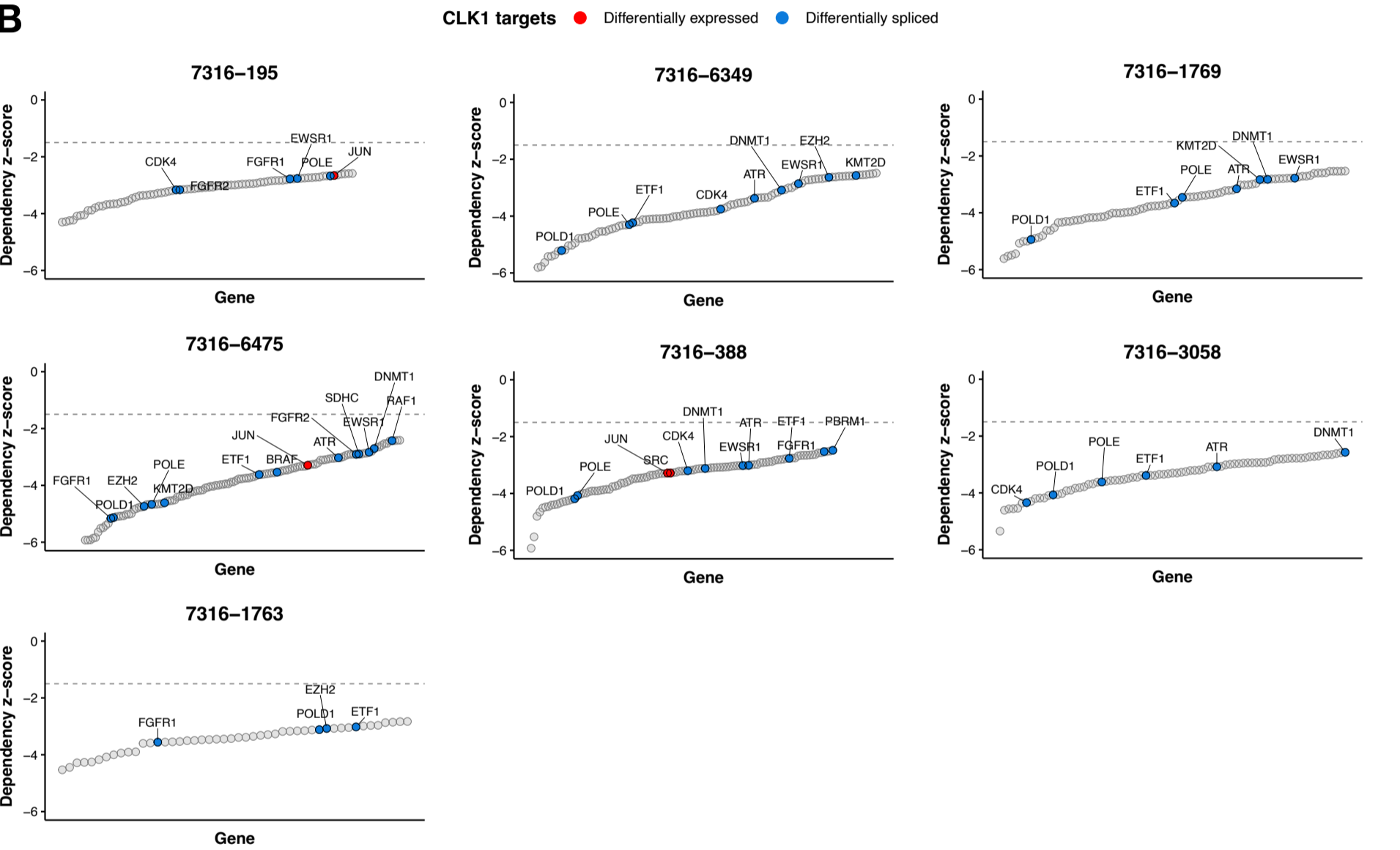

Supplement: Supplement 2 [file media-2.pdf]
