# Supplementary material for: Characterization of aberrant splicing in pediatric central nervous system tumors reveals CLK1 as a candidate oncogenic dependency
Source: bioRxiv. 2024 Aug 22:2024.08.03.606419. Originally published 2024 Aug 6. Preprint. [Version 2] doi: 10.1101/2024.08.03.606419 (PMC11326178; doi:10.1101/2024.08.03.606419)
Supplement: Supplementary file 1 [file NIHPP2024.08.03.606419v2-supplement-6.pdf]

bioRxiv preprint doi: https://doi.org/10.1101/2024.08.03.606419; this version posted August 22, 2024. The copyright holder for this preprint (which was not certified by peer review) is the author/funder, who has granted bioRxiv a license to display the preprint in perpetuity. It is made available under aCC-BY 4.0 International license.

Supplemental Figure Legends

Figure S1. Pearson correlation plots of genome-wide poly-A and stranded PSI values for SE events in PT_RYMG3M91 (**A**) and PT_W5GP3F6B (**B**) showing high concordance across RNA library types. (**C**) UpsetR plot showing recurrent differential splicing events (N ≥ 2) that prefer exon inclusion. Cumulative distribution plots of splicing burden index (SBI) by histology for RI (**D**), A5SS (**E**), and A3SS (**F**) events. Correlation plots for SBI vs TMB across the entire cohort including (**G**) and excluding (**H**) hypermutant and ultra-hyper mutant tumors, or within histology (**I**) excluding hypermutant and ultra-hypermutant tumors. Pearson's R and p-values are shown.

bioRxiv preprint doi: https://doi.org/10.1101/2024.08.03.606419; this version posted August 22, 2024. The copyright holder for this preprint (which was not certified by peer review) is the author/funder, who has granted bioRxiv a license to display the preprint in perpetuity. It is made available under aCC-BY 4.0 International license.

Figure S2. (**A**) Stacked barplot showing tumor histology membership in each cluster stratified by molecular subtype for EPN, HGG, LGG and MB histologies. (**B**) Oncoprint displaying mutation frequencies of splicing factors and spliceosome component genes in HGGs sorted by splicing burden index. Additional annotations include gender, molecular subtype, CNS region, and tumor mutation status.

Figure S3. (A) Over-representation analysis of mis-spliced kinase genes that result in gain/loss of functional sites stratified by **(A)** exon skipping and **(B)** exon skipping. **(C)** Boxplot of *CLK1* exon 4-containing transcript expression in HGGs compared to GTEx normal brain tissues. **(D)** Oncoprint displaying mutation frequencies of key brain cancer genes in HGGs with annotations for *CLK1* exon 4 PSI, *CLK1-201* expression, total *NF1*, *NF1-215* PSI, NF1 pS864 and pS2796 phosphoproteomic abundance, and total NF1 protein abundance z-scores. Additional annotations include gender, molecular subtype, CNS region, and tumor mutation status. All boxplots represent the 25th and 75th percentile and the bar represents the median.

Figure S4. (A) Boxplot of dependency scores stratified by high vs low *CLK1* exon 4 containing transcript expression across all available DepMap brain tumor cell lines. Within histology Wilcoxon p-values are shown. All boxplots represent the 25th and 75th percentile and the bar represents the median. **(B)** Cell viability assay after three days of treatment of KNS-42 cells with increasing concentrations of pan-DYRK/CLK1 inhibitor Cirtuvivint. **(C)** Heatmap displaying single-sample HALLMARK GSVA scores for DS genes affecting functional sites in cells treated with *CLK1* exon 4 morpholino or non-targeting morpholino. **(D)** Barplots illustrating the mean GSVA scores in panel **D** (n = 3 replicates per treatment). **(E)** Heatmap presenting single-sample DNA repair pathway GSVA scores for DS genes affecting functional sites in cells treated with *CLK1* exon 4 morpholino or non-targeting morpholino. **(F)** Barplots displaying mean DNA repair pathway GSVA scores from panel **E** (n = 3 replicates per treatment).

bioRxiv preprint doi: https://doi.org/10.1101/2024.08.03.606419; this version posted August 22, 2024. The copyright holder for this preprint (which was not certified by peer review) is the author/funder, who has granted bioRxiv a license to display the preprint in perpetuity. It is made available under aCC-BY 4.0 International license.

Figure S5. (A) Venn diagram showing the overlap of the total number of DS and DE genes. **(B)** Over-representation analysis of DE genes or **(C)** DS cancer genes that result in gain/loss of functional sites. **(D)** Ranked dotplot of significant CCMA v3 CRISPR gene dependency z-scores in individual CBTN cell lines with *CLK1* expression (red) and splicing-based (blue) target genes highlighted for HGG or **(E)** DMG patient-derived cell lines.

Supplementary Table Legends

Table S1: Sample metadata. (1) Readme and feature definitions. (2) Sample information with clinical metadata and demographics (3) CNS region definition from OpenPedCan[16].

Table S2: Histology-specific splicing events. (1) Exon inclusion related differential splicing events. (2) Exon skipping related differential splicing events. SpliceID includes gene name, mis-spliced exon start and end coordinates, upstream and downstream exon start and end coordinates. (3) Optimal clustering metrics and scoring method information. (4) Cluster membership for each sample.

Table S3: (1) Splicing factor and spliceosome component gene list. (2) DeSeq 2 results comparing high vs low SBI HGG tumors for splicing factors.

Table S4: Differential splicing events impacting functional sites in HGGs. (1) Exon skipping related differential splicing variants. (2) Exon inclusion related differential splicing variants. (3) Functional splice variants subsetted for known kinases.

Table S5: *CLK1* morpholino analyses. (1) Differential gene expression results from DeSeq2 and (2) rMATs results comparing treated with *CLK1* exon 4 morpholino and non-targeting

bioRxiv preprint doi: https://doi.org/10.1101/2024.08.03.606419; this version posted August 22, 2024. The copyright holder for this preprint (which was not certified by peer review) is the author/funder, who has granted bioRxiv a license to display the preprint in perpetuity. It is made available under aCC-BY 4.0 International license.

morpholino. (3) Differential splicing events associated with SE that correspond to known Uniprot functional sites. (4) Differential splicing events associated with A5SS that correspond to known Uniprot functional sites. (5) Differential splicing events associated with A3SS that correspond to known Uniprot functional sites. (6) Differential splicing events associated with RI that correspond to known Uniprot functional sites. (7) Differential splicing of known cancer genes that correspond to known Uniprot functional sites. (8) CLK1 exon 3-4, exon 3-5 and exon 3-5 junction forward and reverse primer sets. (9) Differentially expressed or differentially spliced CLK1 target genes overlapping essential oncogenes defined by CCMA v3.

Figure S1

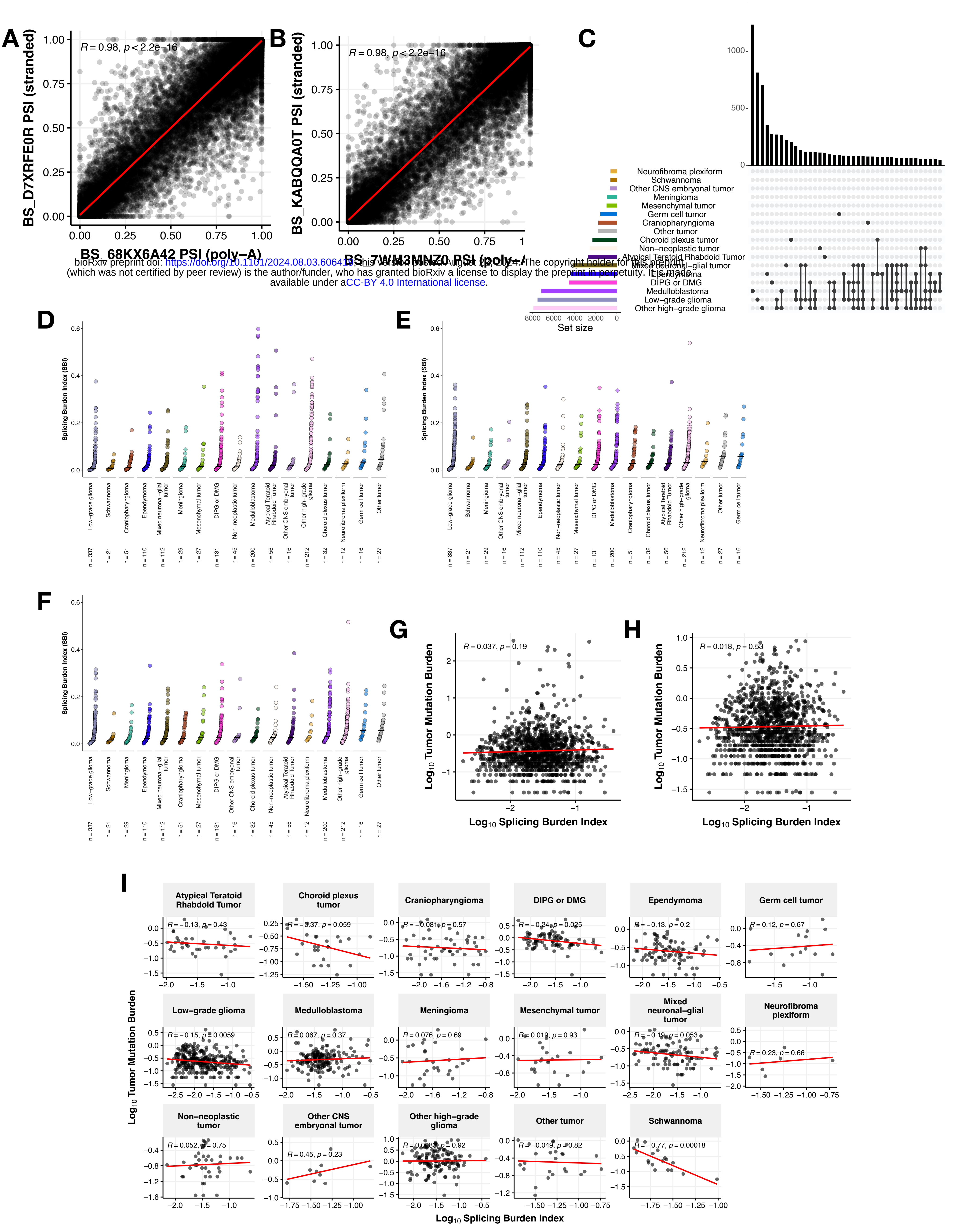

bioRxiv preprint doi: https://doi.org/10.1101/2024.08.03.606419; this version posted August 22, 2024. The copyright holder for this preprint (which was not certified by peer review) is the author/funder, who has granted bioRxiv a license to display the preprint in perpetuity. It is made available under aCC-BY 4.0 International license.

Figure S2

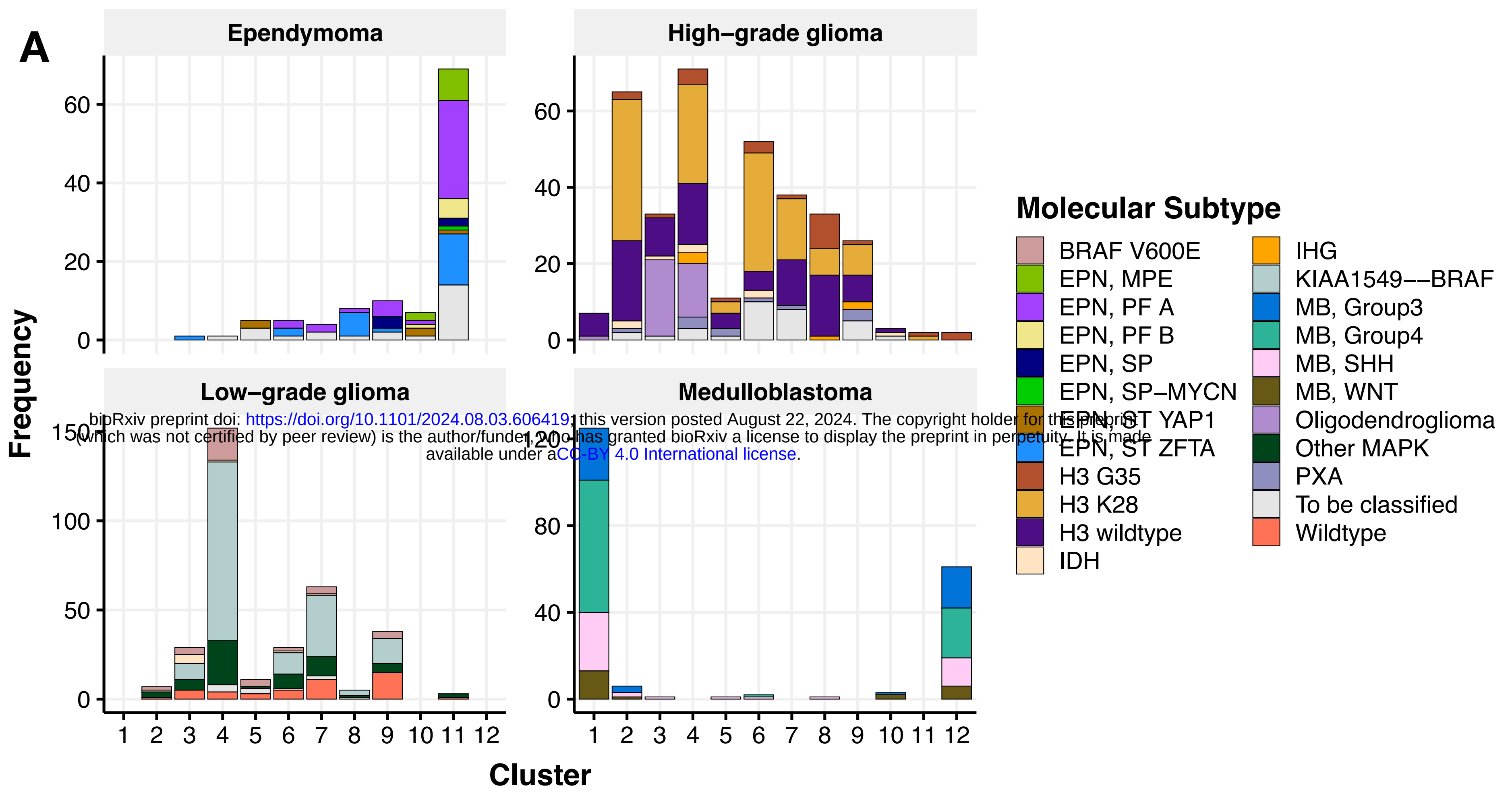

bioRxiv preprint doi: https://doi.org/10.1101/2024.08.03.606419; this version posted August 22, 2024. The copyright holder for this preprint (which was not certified by peer review) is the author/funder, who has granted bioRxiv a license to display the preprint in perpetuity. It is made available under aCC-BY 4.0 International license.

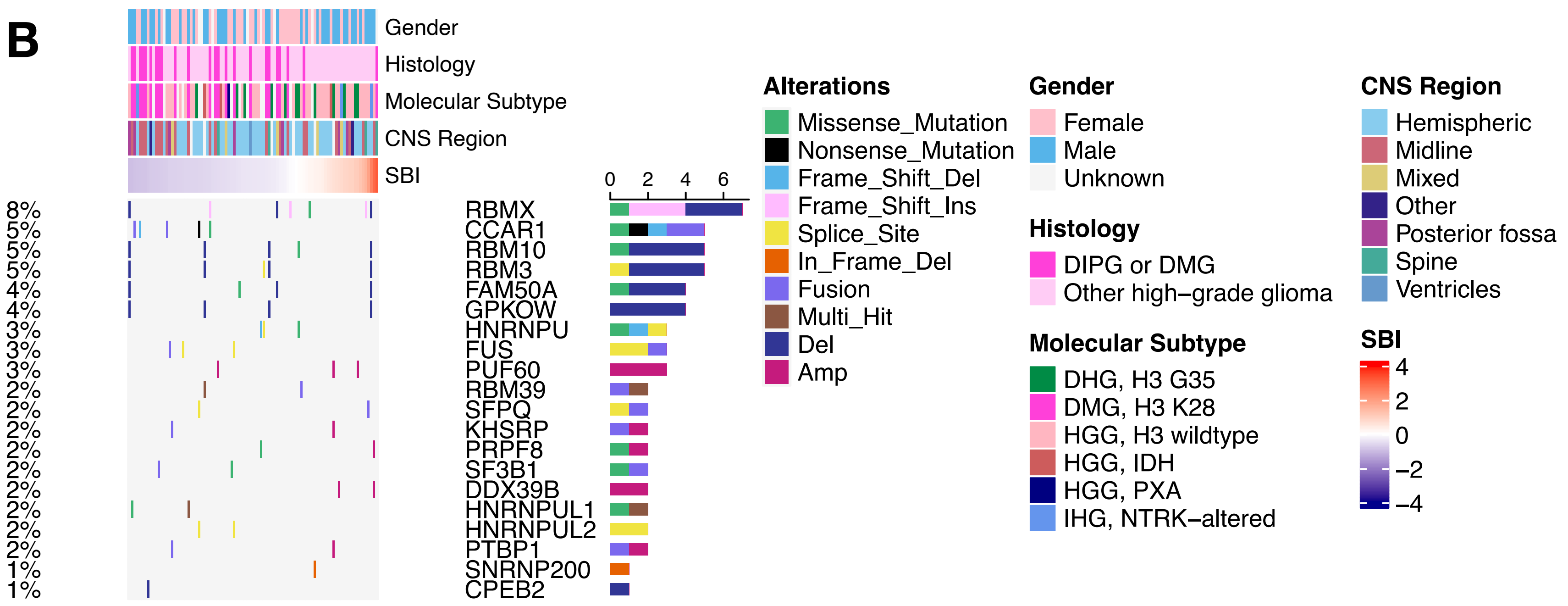

Figure S3

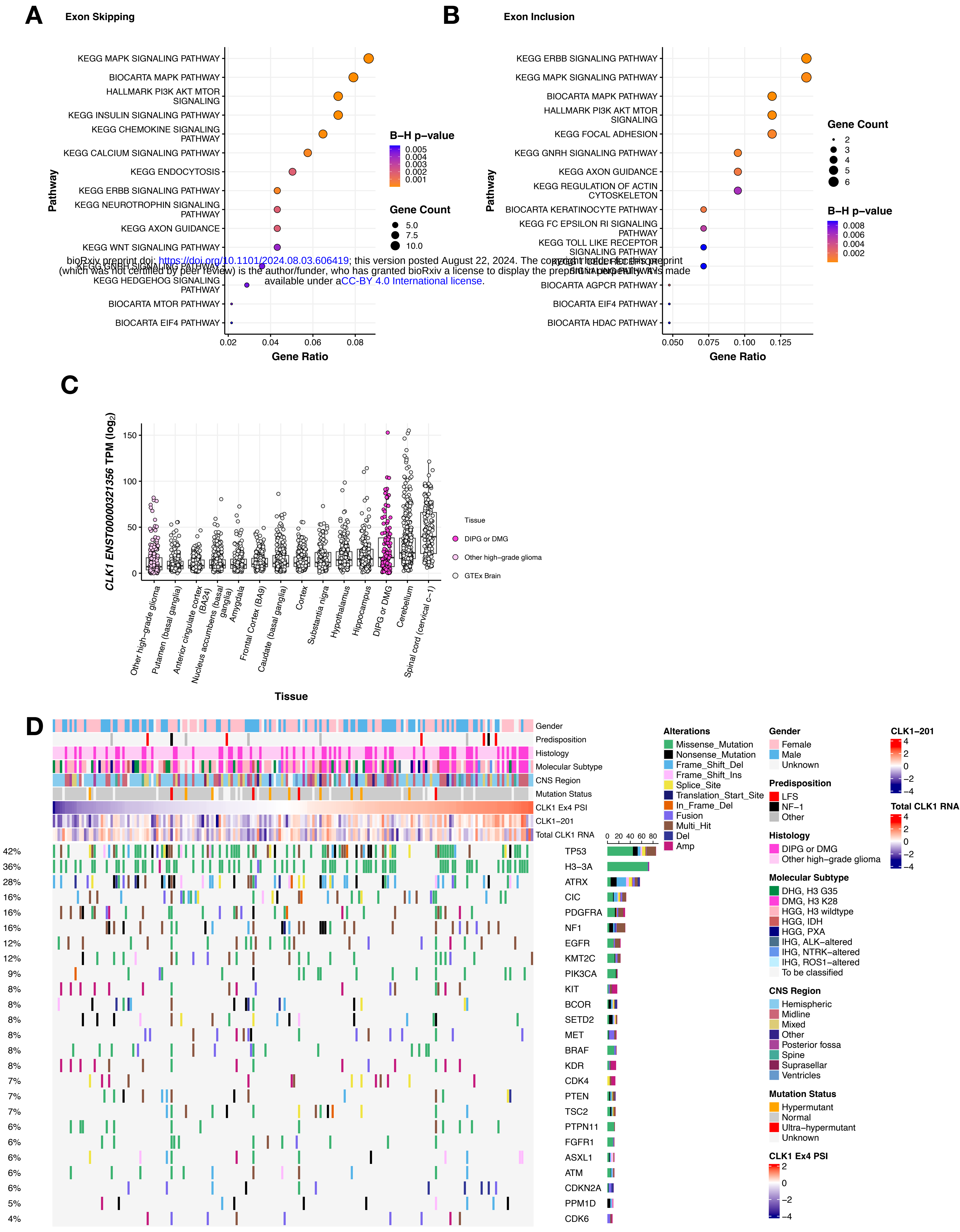

bioRxiv preprint doi: https://doi.org/10.1101/2024.08.03.606419; this version posted August 22, 2024. The copyright holder for this preprint (which was not certified by peer review) is the author/funder, who has granted bioRxiv a license to display the preprint in perpetuity. It is made available under aCC-BY 4.0 International license.

Figure S4

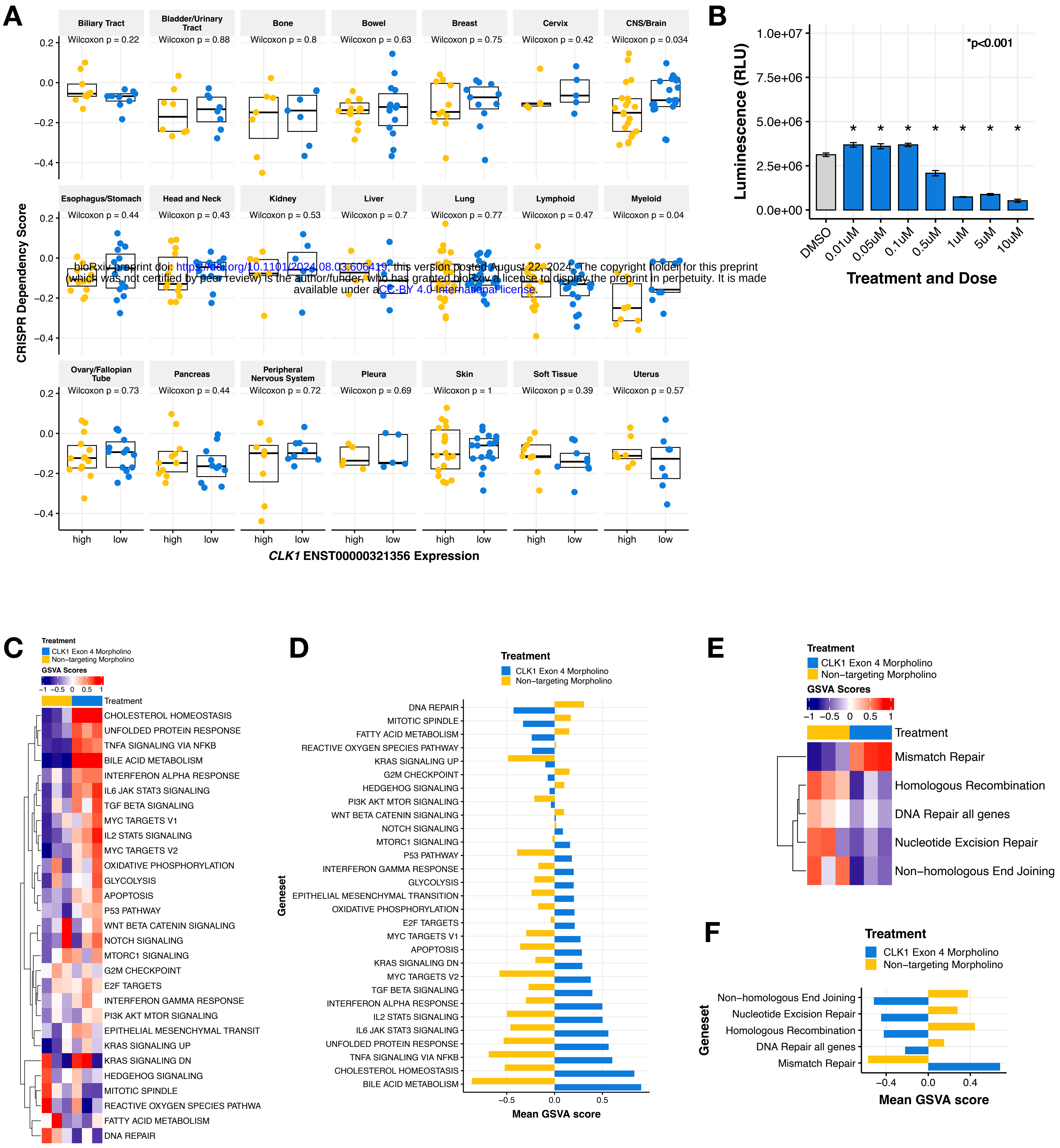

bioRxiv preprint doi: https://doi.org/10.1101/2024.08.03.606419; this version posted August 22, 2024. The copyright holder for this preprint (which was not certified by peer review) is the author/funder, who has granted bioRxiv a license to display the preprint in perpetuity. It is made available under aCC-BY 4.0 International license.

Figure S5

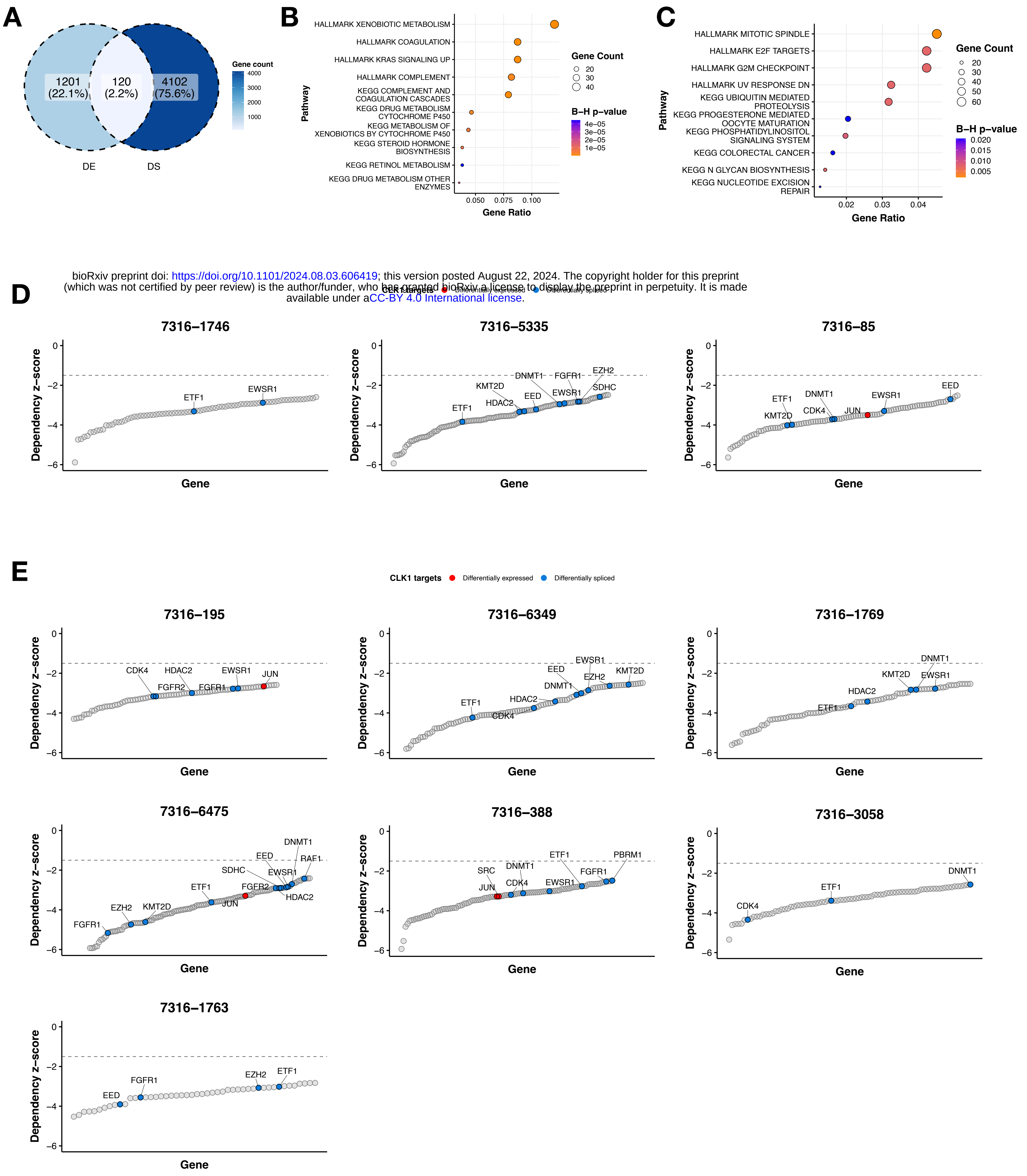

bioRxiv preprint doi: https://doi.org/10.1101/2024.08.03.606419; this version posted August 22, 2024. The copyright holder for this preprint (which was not certified by peer review) is the author/funder, who has granted bioRxiv a license to display the preprint in perpetuity. It is made available under aCC-BY 4.0 International license.
