# Supplementary material for: The splicing modulator CLK1 is a candidate oncogenic dependency in pediatric high-grade gliomas
Source: bioRxiv. 2024 Aug 6:2024.08.03.606419. Preprint. [Version 1] doi: 10.1101/2024.08.03.606419 (PMC11326178; doi:10.1101/2024.08.03.606419)

bioRxiv preprint doi: https://doi.org/10.1101/2024.08.03.606419; this version posted August 6, 2024. The copyright holder for this preprint (which was not certified by peer review) is the author/funder, who has granted bioRxiv a license to display the preprint in perpetuity. It is made available under aCC-BY 4.0 International license.

## Supplemental Figure Legends

**Figure S1.** Pearson correlation plots of genome-wide poly-A and stranded PSI values for SE events in PT_RYMG3M91 (**A**) and PT_W5GP3F6B (**B**) showing high concordance across RNA library types. (**C**) UpsetR plot showing recurrent differential splicing events (N ≥ 2) that prefer exon inclusion. Cumulative distribution plots of splicing burden index (SBI) by histology for RI (**D**), A5SS (**E**), and A3SS (**F**) events. Correlation plots for SBI vs TMB across the entire cohort including (**G**) and excluding (**H**) hypermutant and ultra-hyper mutant tumors, or within histology (**I**) excluding hypermutant and ultra-hypermutant tumors. Pearson's R and p-values are shown.

**Figure S2.** (**A**) Stacked barplot showing tumor histology membership in each cluster stratified by molecular subtype for EPN, HGG, LGG and MB histologies. (**B**) Oncoprint displaying mutation frequencies of splicing factors and spliceosome component genes in HGGs sorted by splicing burden index. Additional annotations include gender, molecular subtype, CNS region, and tumor mutation status.

**Figure S3. (A)** Over-representation analysis of mis-spliced kinase genes that result in gain/loss of functional sites stratified by **(A)** exon skipping and **(B)** exon skipping. **(C)** Boxplot of *CLK1* exon 4-containing transcript expression in HGGs compared to GTEx normal brain tissues. **(D)** Oncoprint displaying mutation frequencies of key brain cancer genes in HGGs with annotations for *CLK1* exon 4 PSI, *CLK1-201* expression, total *NF1*, *NF1-215* PSI, NF1 pS864 and pS2796 phosphoproteomic abundance, and total NF1 protein abundance z-scores. Additional annotations include gender, molecular subtype, CNS region, and tumor mutation status. All boxplots represent the 25th and 75th percentile and the bar represents the median.

**Figure S4. (A)** Boxplot of dependency scores stratified by high vs low *CLK1* exon 4 containing transcript expression across all available DepMap brain tumor cell lines. Within histology

bioRxiv preprint doi: https://doi.org/10.1101/2024.08.03.606419; this version posted August 6, 2024. The copyright holder for this preprint (which was not certified by peer review) is the author/funder, who has granted bioRxiv a license to display the preprint in perpetuity. It is made available under aCC-BY 4.0 International license.

Wilcoxon p-values are shown. All boxplots represent the 25th and 75th percentile and the bar represents the median. **(B)** Cell viability assay after three days of treatment of KNS-42 cells with increasing concentrations of pan-DYRK/CLK1 inhibitor Cirtuvivint. **(C)** Heatmap displaying single-sample HALLMARK GSVA scores for DS genes affecting functional sites in cells treated with *CLK1* exon 4 morpholino or non-targeting morpholino. **(D)** Barplots illustrating the mean GSVA scores in panel **D** (n = 3 replicates per treatment). **(E)** Heatmap presenting single-sample DNA repair pathway GSVA scores for DS genes affecting functional sites in cells treated with *CLK1* exon 4 morpholino or non-targeting morpholino. **(F)** Barplots displaying mean DNA repair pathway GSVA scores from panel **E** (n = 3 replicates per treatment).

**Figure S5. (A)** Venn diagram showing the overlap of the total number of DS and DE genes. **(B)** Over-representation analysis of DE genes or **(C)** DS cancer genes that result in gain/loss of functional sites. **(D)** Ranked dotplot of significant CCMA v3 CRISPR gene dependency z-scores in individual CBTN cell lines with *CLK1* expression (red) and splicing-based (blue) target genes highlighted for HGG or **(E)** DMG patient-derived cell lines.

**Figure S1**

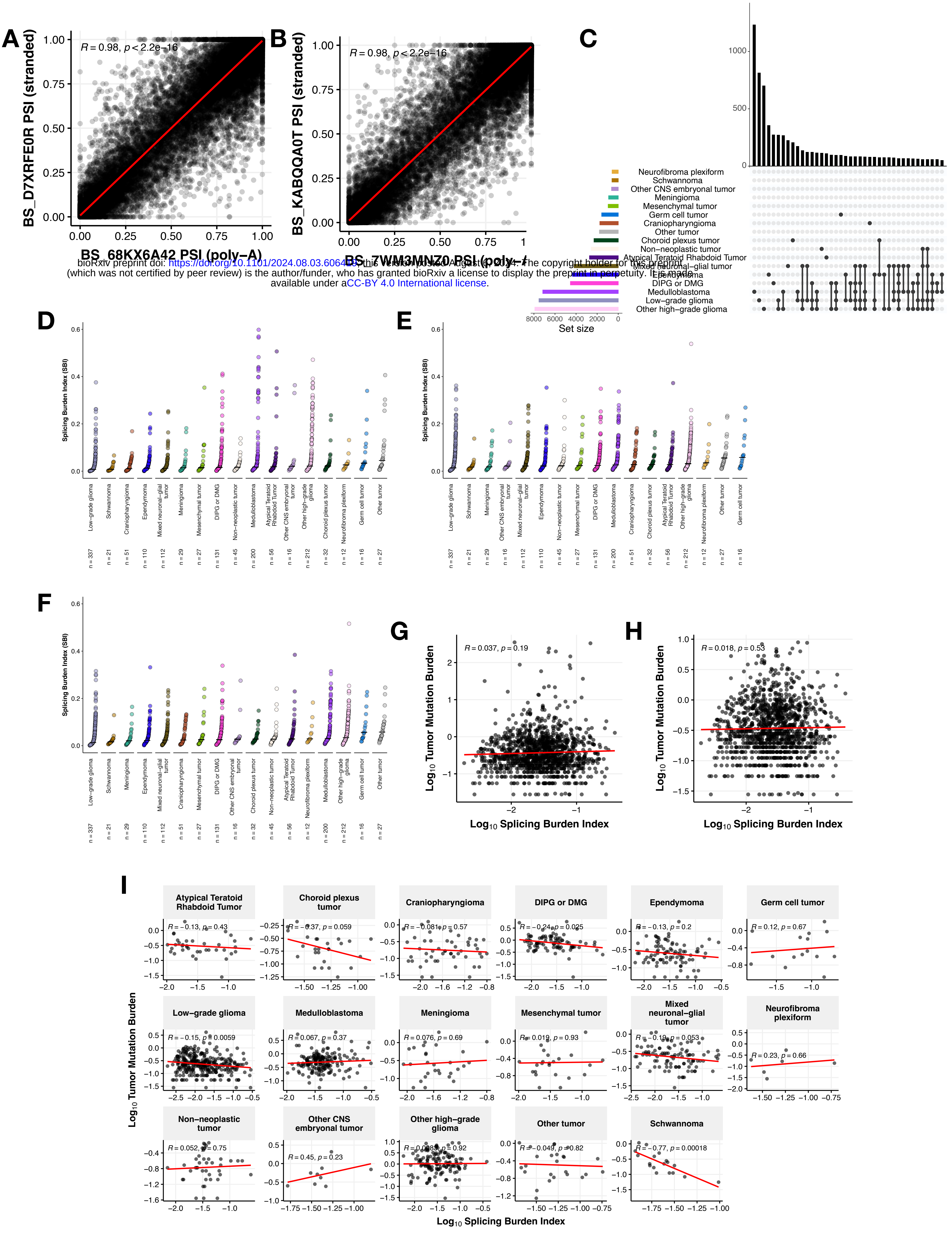


bioRxiv preprint doi: https://doi.org/10.1101/2024.08.03.606419; this version posted August 4, 2024. The copyright holder for this preprint (which was not certified by peer review) is the author/funder, who has granted bioRxiv a license to display the preprint in perpetuity. It is made available under aCC-BY 4.0 International license.

**Figure S2**

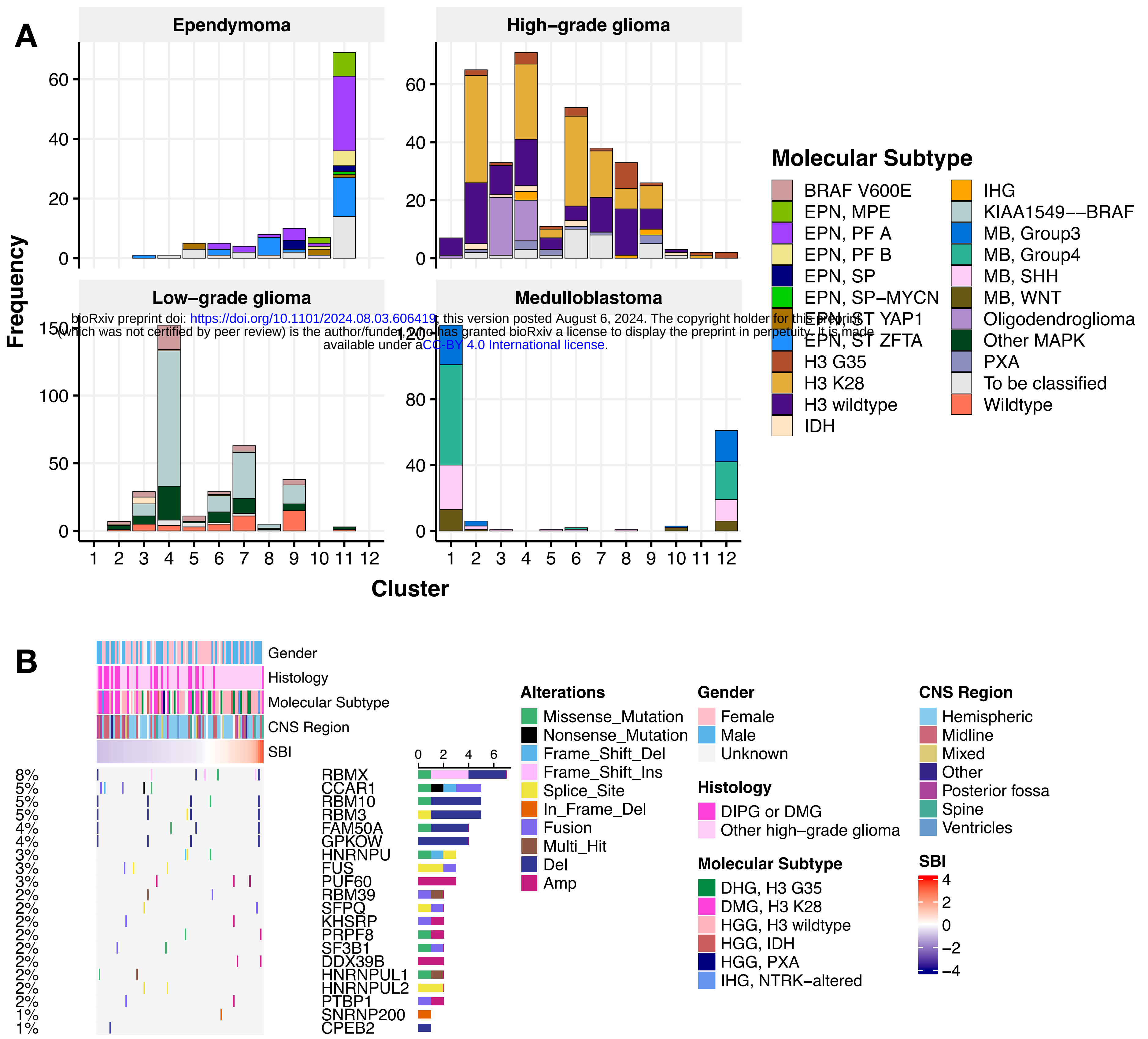


bioRxiv preprint doi: https://doi.org/10.1101/2024.08.03.606419; this version posted August 6, 2024. The copyright holder for this preprint (which was not certified by peer review) is the author/funder, who has granted bioRxiv a license to display the preprint in perpetuity. It is made available under aCC-BY 4.0 International license.

# Figure S3

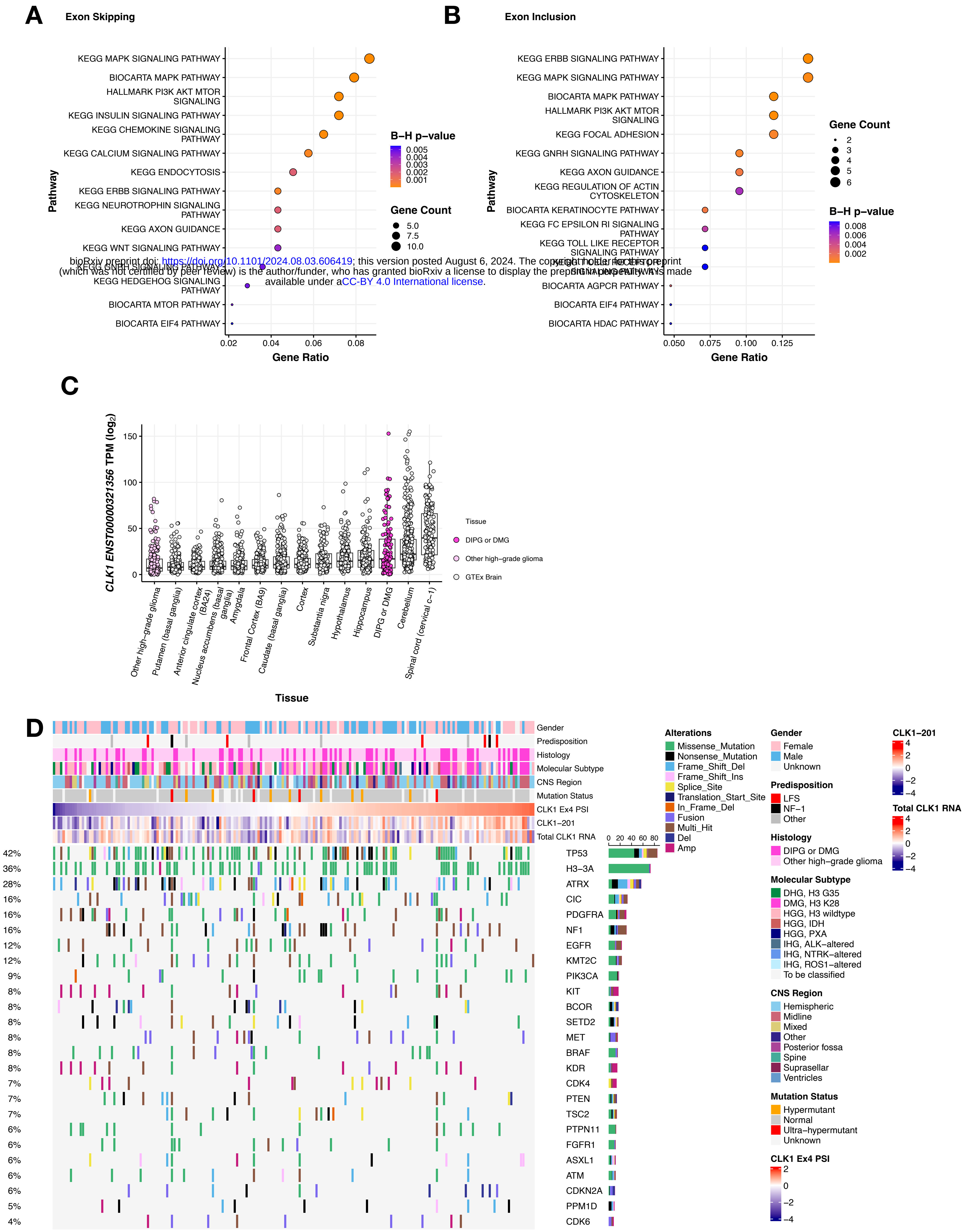


bioRxiv preprint doi: https://doi.org/10.1101/2024.08.03.606419; this version posted August 6, 2024. The copyright holder for this preprint (which was not certified by peer review) is the author/funder, who has granted bioRxiv a license to display the preprint in perpetuity. It is made available under aCC-BY 4.0 International license.

# Figure S4

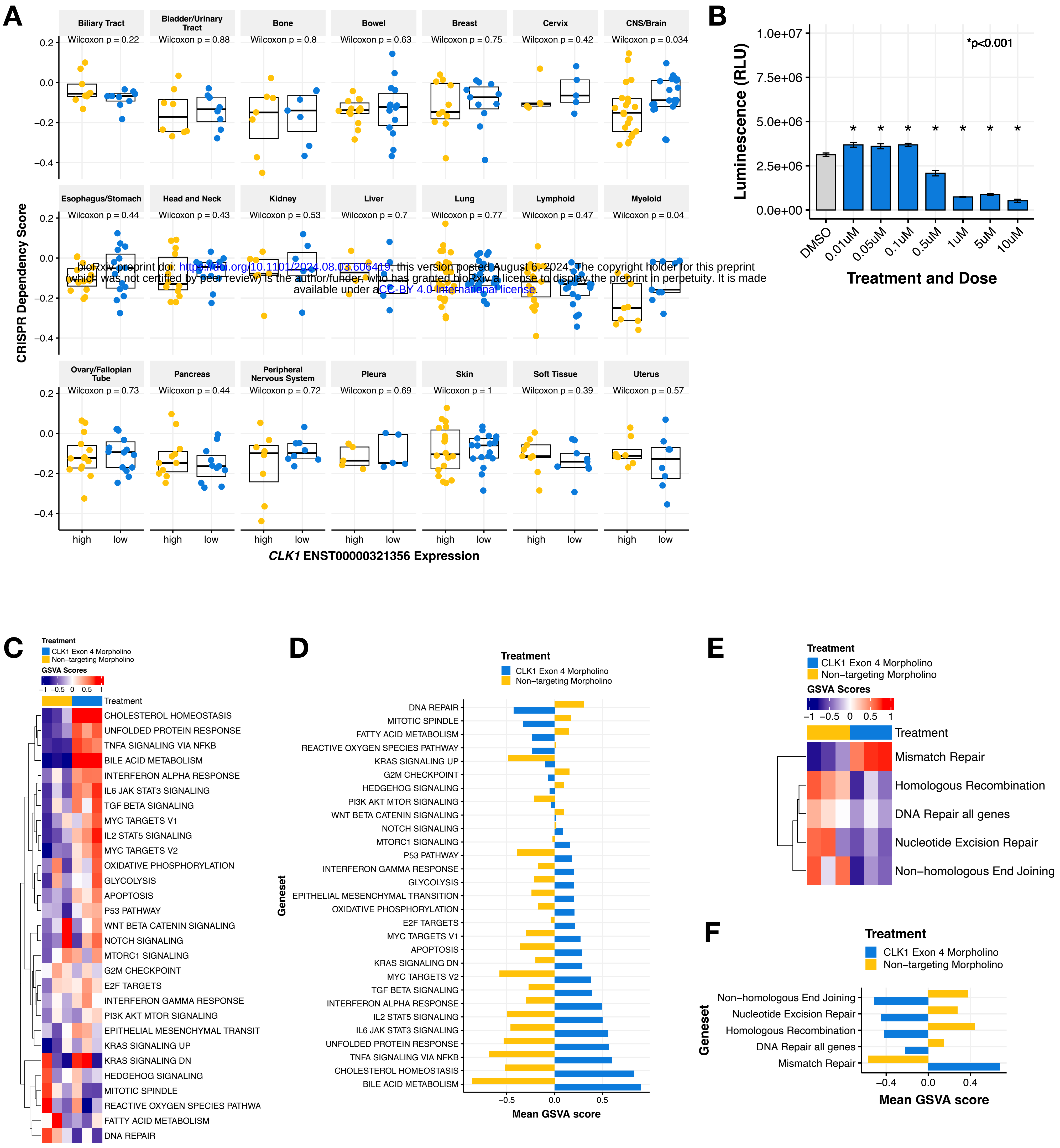


bioRxiv preprint doi: https://doi.org/10.1101/2024.08.03.606419; this version posted August 6, 2024. The copyright holder for this preprint (which was not certified by peer review) is the author/funder, who has granted bioRxiv a license to display the preprint in perpetuity. It is made available under aCC-BY 4.0 International license.

# Figure S5

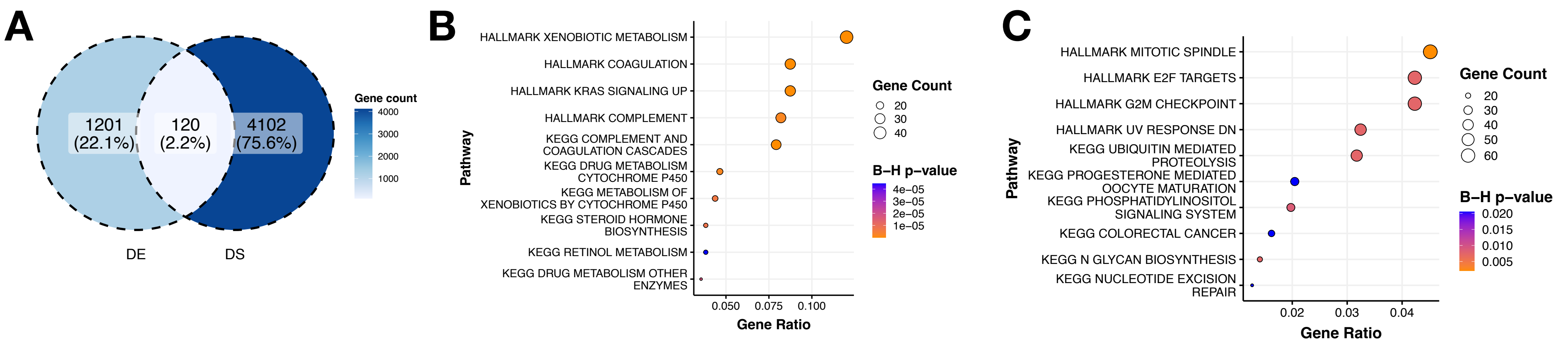


bioRxiv preprint doi: https://doi.org/10.1101/2024.08.03.606419; this version posted August 6, 2024. The copyright holder for this preprint (which was not certified by peer review) is the author/funder, who has granted bioRxiv a license to display the preprint in perpetuity. It is made available under aCC-BY 4.0 International license.

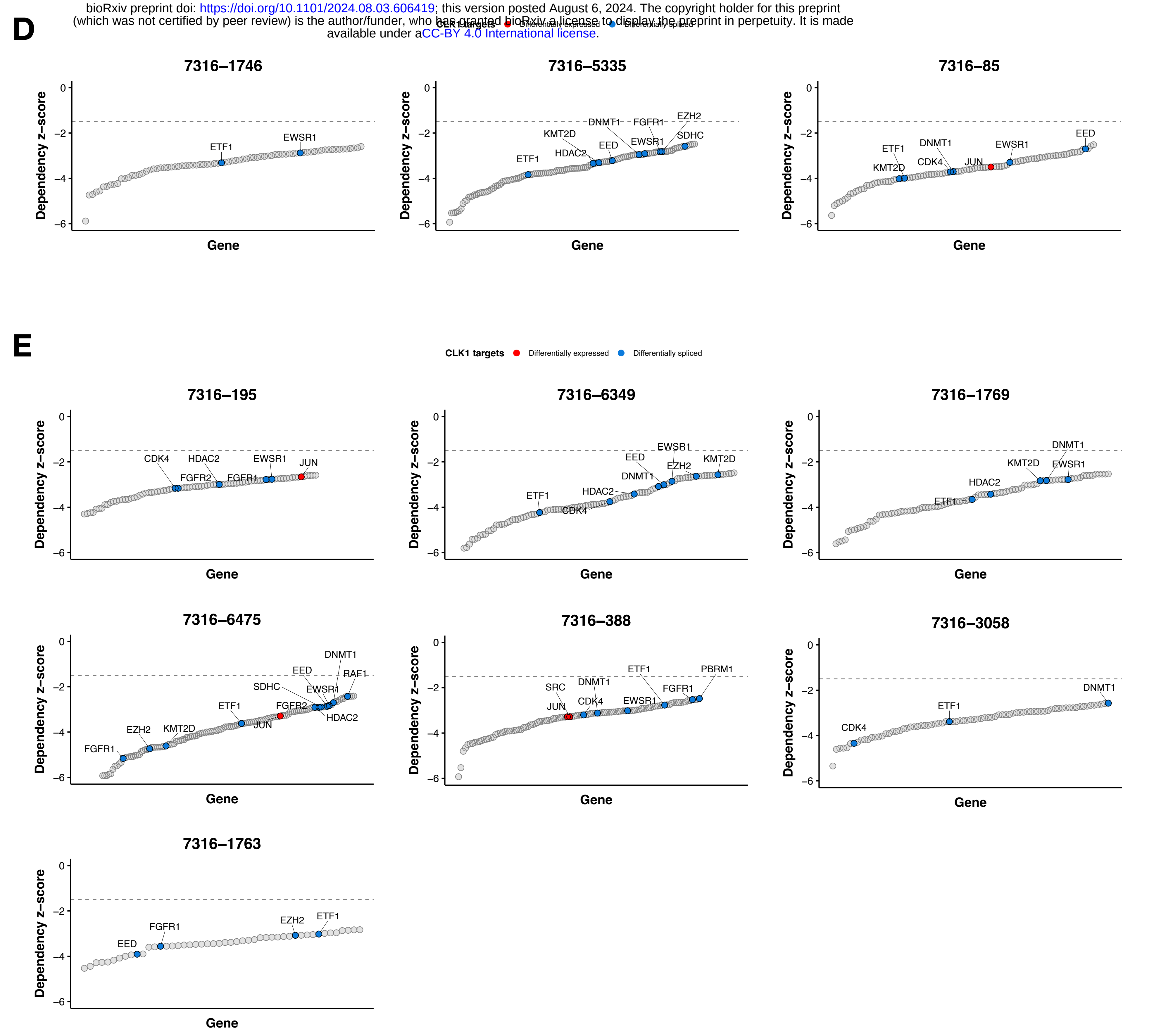

Supplement: Supplementary file 1 [file NIHPP2024.08.03.606419v1-supplement-6.pdf]
